# Supplementary material for: Evaluation of microalbuminuria as a prognostic indicator after a TIA or minor stroke in an outpatient setting: the prognostic role of microalbuminuria in TIA evolution (ProMOTE) study
Source: BMJ Open. 2021 Sep 6;11(9):e043253. doi: 10.1136/bmjopen-2020-043253 (PMC8422314; doi:10.1136/bmjopen-2020-043253)
Supplement: Supplementary data [file bmjopen-2020-043253supp001.pdf]

Supplementary Table 1: APA (Age, Previous Stroke and ACR) Score derivation

	Variable	Value
**Age**	≥80 years	1
**Previous stroke or TIA**	Present	1
**ACR**	≥3.5 mg/mmol for women<br>≥2.5 mg/mol for men	1

**Score**	0-1	2-3
**Risk**	Low Risk	High Risk

ACR: Albumin Creatinine Ratio

Supplementary Table 2: Occurrence of major adverse cardiac events (MACE) by APA risk score group (p for Chi-Squared)

APA score	N	Occurrence of MACE events (%)
**0**	1215	4.9
**1**	770	7.0
**2**	207	11.6
**3**	10	30
		P<0.001

Supplementary Table 3
The predictive values of the elements of the APA score compared to the total score in the study and the validation population.

Table 3a The predictive values in the original study population

	PPV	NPV	Sensitivity	Specificity
Aged >80 years	7.9%	94.2%	32.1%	74.7%
Previous Stroke	11.9%	93.9%	7.1%	96.4%
Age >80 years and Previous Stroke	8.4%	94.5%	37.9%	72.1%
APA score ≥2	12.4%	94.6%	19.3%	91.2%

Table 3b The predictive values in the validation population

	PPV	NPV	Sensitivity	Specificity
Aged >80 years	9.8%	91.3%	38.5%	64.6%
Previous Stroke	18.2%	93.6%	46.2%	79.2%
Age >80 years and Previous Stroke	13.3%	91.4%	15.4%	90.0%
APA score ≥2	24.4%	96.9%	76.9%	75.4%

PPV Positive Predictive Value
NPV Negative Predictive Value
APA Composite of Age >80 years, Previous stroke, elevated ACR. A score of 2 or 3 are regarded as high risk
