# Supplementary material for: Evaluation of microalbuminuria as a prognostic indicator after a TIA or minor stroke in an outpatient setting: the prognostic role of microalbuminuria in TIA evolution (ProMOTE) study
Source: BMJ Open. 2021 Sep 6;11(9):e043253. doi: 10.1136/bmjopen-2020-043253 (PMC8422314; doi:10.1136/bmjopen-2020-043253)
Supplement: Author's manuscript [file bmjopen-2020-043253.draft_revisions.pdf]

BMJ Open

BMJ Open is committed to open peer review. As part of this commitment we make the peer review history of every article we publish publicly available.

When an article is published we post the peer reviewers' comments and the authors' responses online. We also post the versions of the paper that were used during peer review. These are the versions that the peer review comments apply to.

The versions of the paper that follow are the versions that were submitted during the peer review process. They are not the versions of record or the final published versions. They should not be cited or distributed as the published version of this manuscript.

BMJ Open is an open access journal and the full, final, typeset and author-corrected version of record of the manuscript is available on our site with no access controls, subscription charges or pay-per-view fees (http://bmjopen.bmj.com).

If you have any questions on BMJ Open's open peer review process please email info.bmjopen@bmj.com

BMJ Open

# Microalbuminuria Improves Risk Prediction of Major Adverse Cardiac Events after Transient Ischaemic Attack (TIA) and Minor Stroke: The Prognostic Role of Microalbuminuria in TIA Evolution (ProMOTE) Study



| | |
|---|---|
| Journal: | *BMJ Open* |
| Manuscript ID | bmjopen-2020-043253 |
| Article Type: | Original research |
| Date Submitted by the Author: | 14-Sep-2020 |





| | |
|---|---|
| Complete List of Authors: | Strain, W; University of Exeter, Diabetes and Vascular Research Centre; Royal Devon and Exeter NHS Foundation Trust, Academic Department of Healthcare for Older People<br>Elyas, Salim; University of Exeter, Diabetes and Vascular Research Centre; Royal Devon and Exeter NHS Foundation Trust, Academic Department of Healthcare for Older People<br>Wedge, Nicola; Royal Devon and Exeter NHS Foundation Trust, Academic Department of Healthcare for Older People<br>Mounce, Luke; University of Exeter, Institute of Health Research<br>Henley, W; University of Exeter Medical School, Institute of Health Research<br>James, Martin; Royal Devon and Exeter NHS Foundation Trust, Academic Department of Healthcare for Older People<br>Shore, Angela; University of Exeter Medical School, NIHR Exeter Clinical Research Facility |





| | |
|---|---|
| Keywords: | Stroke medicine < INTERNAL MEDICINE, STROKE MEDICINE, Risk management < HEALTH SERVICES ADMINISTRATION & MANAGEMENT |
| | |



SCHOLARONE™
Manuscripts

*I, the Submitting Author has the right to grant and does grant on behalf of all authors of the Work (as defined in the below author licence), an exclusive licence and/or a non-exclusive licence for contributions from authors who are: i) UK Crown employees; ii) where BMJ has agreed a CC-BY licence shall apply, and/or iii) in accordance with the terms applicable for US Federal Government officers or employees acting as part of their official duties; on a worldwide, perpetual, irrevocable, royalty-free basis to BMJ Publishing Group Ltd ("BMJ") its licensees and where the relevant Journal is co-owned by BMJ to the co-owners of the Journal, to publish the Work in this journal and any other BMJ products and to exploit all rights, as set out in our licence.*

*The Submitting Author accepts and understands that any supply made under these terms is made by BMJ to the Submitting Author unless you are acting as an employee on behalf of your employer or a postgraduate student of an affiliated institution which is paying any applicable article publishing charge ("APC") for Open Access articles. Where the Submitting Author wishes to make the Work available on an Open Access basis (and intends to pay the relevant APC), the terms of reuse of such Open Access shall be governed by a Creative Commons licence – details of these licences and which Creative Commons licence will apply to this Work are set out in our licence referred to above.*

*Other than as permitted in any relevant BMJ Author's Self Archiving Policies, I confirm this Work has not been accepted for publication elsewhere, is not being considered for publication elsewhere and does not duplicate material already published. I confirm all authors consent to publication of this Work and authorise the granting of this licence.*

For peer review only

# Microalbuminuria Improves Risk Prediction of Major Adverse Cardiac Events after Transient Ischaemic Attack (TIA) and Minor Stroke: The Prognostic Role of Microalbuminuria in TIA Evolution (ProMOTE) Study

Running Title: Predictive role of microalbuminuria after TIA and minor strokes

**W David Strain[1,2,4],**

**Salim Elyas[1,2],**

**Nicola Wedge[2,4],**

**Luke Mounce[3],**

**William Henley[3],**

**Martin A James[2],**

**Angela C Shore[1,4]**

1 Diabetes and Vascular Research Centre, University of Exeter Medical School. Barrack Road, Exeter EX2 5AX

2 Academic Department of Healthcare for Older People, Royal Devon & Exeter Hospital Barrack Road, Exeter EX2 5DW

3 University of Exeter, College of Medicine and Health, Exeter, UK

4 NIHR Exeter Clinical Research Facility

**Address for Correspondence:**

Dr David Strain

Diabetes and Vascular Research Centre

University of Exeter Medical School, Royal Devon & Exeter Hospital

Barrack Road, Exeter. EX2 5AX UK

**Email. d.strain@exeter.ac.uk**

**Tel: +441392 403058**

**Fax: +441392 403027**

For peer review only

| | | |
|---|---|---|
| **Word count** | Manuscript | 3168 |
| | Abstract | 334 |

**Disclosures**

All authors confirm that there are no conflicts of interest

For peer review only



For peer review only - http://bmjopen.bmj.com/site/about/guidelines.xhtml

**Abstract**:

**Objective**: Transient ischaemic attacks (TIA) and minor strokes are important risk factors for further vascular events. We explored the role of albumin-creatinine ratio (ACR) in improving risk prediction after a first event.

**Setting**: Rapid access stroke clinics in the UK

**Participants**. 2,202 patients attending with TIA or minor stroke diagnosed by the attending stroke physician, able to provide a urine sample to evaluate albumin creatinine ratio (ACR) using a near patient testing device.

**Primary and secondary outcomes**. Primary outcome was Major Adverse Cardiac Events (MACE; recurrent stroke, myocardial infarction, or cardiovascular death) at 90 days. The key secondary outcome was to determine whether urinary ACR could contribute to a risk prediction tool for use in a clinic setting.

**Results**: 151 MACE occurred in 144 participants within 90 days. Participants with a MACE had higher ACR than those without. A composite score awarding a point each for Age >80, Previous stroke/TIA and presence of microalbuminuria identified those at low and high risk. 90% of patients were at low risk (scoring 0 or 1). Their 90 day risk of a MACE was 5.7%. Of the remaining "high risk" population (scoring 2 or 3) 12.4% experienced MACE over 90 days ($p<0.001$ compared to low risk population). The need for acute admission in the first 7 days was two-fold elevated in the high-risk group compared to the low risk (3.23 vs. 1.43%; $p=0.05$). These findings were validated in an independent historic sample.

**Conclusion**: A risk score comprising age, previous stroke/TIA and microalbuminuria predicts future MACE events whilst identifying those at low risk of a recurrent event. This tool shows promise in the risk stratification of patients to avoid the admission of

For peer review only

low risk patients whilst assigning intensive interventions such as dual anti-platelet therapy to those at highest risk.

**Keywords**:

Stroke,

TIA,

Microalbuminuria,

ACR,

Risk prediction.

For peer review only

## Article Summary

*Strengths and limitations of this study*

- The pragmatic design of this study, provides good generalisability to clinical practice
- The predictive role of the combination of urinary ACR in combination with very basic demographics would be able to reassure the 90% of the population that there was only approximately a 1 in 20 risk of an event recurring, whereas the higher risk population had a 1 in 8 chance of a MACE outcome
- The study is limited by the availability of data to that which was available to the physicians at the time of the stroke appointment. This limits the ability to determine mechanistic association between ACR and MACE outcome
- Further work is required to determine whether therapies that reduce ACR can modify the risk of subsequent MACE

For peer review only

**Introduction:**

Stroke is the second most common cause of death and a leading cause of disability worldwide[1]. Despite, or possibly because of, recent trends in reducing stroke mortality, the health and social disability burden of stroke is increasing[2]. Transient ischaemic attacks (TIA) and minor strokes are the most important risk factors for recurrent stroke and predict long term mortality.[3,4] 23% of patients presenting with stroke have a history of TIA in the 3 months prior to the index event.[5] This is a key population to target for secondary prevention, but these patients represent less than 10% of all those who present to rapid access TIA clinics.[6,7] Notably, half of all completed strokes occur within the first week after TIA or minor stroke.[5]

Accurate identification of those patients presenting to TIA clinics with TIA or minor stroke who are most at risk for future events is important in order to i) intensify treatments such as giving dual anti-platelet therapy with aspirin and clopidogrel in combination where it is apparent that benefits outweigh risks[8,9] ii) guide the need for urgent admission to facilitate the detection and urgent surgical correction of severe internal carotid artery (ICA) stenosis[10] iii) reassure patients with lowest risk.

Several risk stratification tools are already utilised in such clinics such as ABCD2[11,12] (awarding points for Age, presenting Blood pressure, Clinical features of unilateral weakness or aphasia, Duration of symptoms and Diabetes), California score[6] and imaging based scoring systems.[10,13] These tools lack optimal sensitivity and specificity.[12,14] Indeed some studies suggest patients with a 'low risk' ABCD2 score (less than 4/7) have similar 90 day stroke risk as patients deemed high risk with an ABCD2 score



For peer review only - http://bmjopen.bmj.com/site/about/guidelines.xhtml

above 4/7,[15] whilst missing up to 40% of patients with severe ICA stenosis.[16] As such, recent consensus guidelines have advised against their use.[17-19]

Increased urinary albumin excretion rate (AER) has been shown to predict incident stroke and heart failure in people with diabetes. In the general population, AER predicts cardiovascular disease and mortality post-stroke independent of conventional cardiovascular risk factors such as hypertension, diabetes and smoking [20-23]. Urinary albumin creatinine ratio (ACR) is a well recognised proxy for urinary AER, and can now be assessed using simple and inexpensive point of care equipment. In an earlier pilot study of 142 patients with minor stroke/TIA,[24] we identified the potential role of urinary ACR in identifying those at highest risk of a recurrent event. Although statistically significant, this study was not large enough to explore potential confounding. We therefore performed a definitive study to assess whether urinary ACR improved the risk stratification of patients presenting with TIA or minor stroke to UK acute stroke clinics.

**Methods:**

Patients presenting with suspected minor stroke/TIA to rapid access clinics in one of 12 UK hospitals were recruited at the end of their clinic consultation. Routine clinical care would include initiation of single antiplatelet, cholesterol-lowering and antihypertensive treatment as soon as possible, rapid access carotid and cerebral imaging (where appropriate), and, for those patients with a relevant severe ICA stenosis (> 50% diameter stenosis by the North American Symptomatic Carotid Endarterectomy Trial (NASCET) method,[25] urgent carotid revascularization. If a cardioembolic source was identified, anticoagulants were initiated according to local protocols. After written,



For peer review only - http://bmjopen.bmj.com/site/about/guidelines.xhtml

informed consent was obtained in the clinic, demographics, including age, sex, height, weight, past medical history and ABCD2 score were recorded. Times from onset of symptoms to assessment and enrollment in the study were documented. A clean specimen of urine was collected from participants in the clinic and tested with a Unistix dipstick and, if there was no indication of urinary tract infection (presence of any two of leucocytes, nitrites and/or protein), ACR was measured using a point of care analyzer (The Afinion™ AS100 Analyzer, Axis Shield, Dundee, UK). This system uses an immunometric membrane flow-through principle for albumin measurement and an enzymatic colorimetric test for creatinine quantification[26] and reports an ACR in approximately 5 minutes within the range 0.1 to 140 mg/mmol with a coefficient of variation of 4.6-6%.[26] Participants with values of less than 0.1 mg/mmol were recorded as having the lowest recordable value of ACR (0.1 mg/mmol).

Participants were followed up by telephone on day 7, 30 and 90, and any history of any further vascular events, hospitalisation or death was obtained. If contact with participants was not possible initially, the presence or absence of further events was verified during additional attempts at contact or during future follow up. If no further contact was possible with the participant (e.g. in the case of significant stroke or death), medical history was collected from the next of kin and verified from the primary care physician and hospital records. All reported clinical events were adjudicated according to standardised diagnostic criteria by a data monitoring committee including two independent stroke physicians, blinded to the ACR, by examination of clinical records if they attended hospital, or their primary care physician, or by verifying their clinical symptoms from the research records if patients did not seek further medical advice. In the event of disagreement, a third independent stroke physician acted as adjudicator.

For peer review only



For peer review only - http://bmjopen.bmj.com/site/about/guidelines.xhtml

The primary outcome was a time to first major adverse cardiovascular event (MACE; recurrent stroke, myocardial infarction or death) within 90 days. Secondary outcomes were time to recurrent stroke, total mortality (even if these were not the first MACE event), the presence of first MACE within 7 days, and the need for hospitalisation within the first 7 days (including all cause mortality and need for urgent endarterectomy). The study protocol was approved by the national research ethics committee (approval 14/EE/1106).

*Patient involvement*

Patients were involved in the design and conduct of this research. During the feasibility stage, priority of the research question, and methods of recruitment were informed by discussions with patients through a focus group session and three structured interviews. During the trial, two patients joined the independent trial steering committee. Once the trial has been published, participants will be informed of the results through a dedicated newsletter suitable for a non-specialist audience, led by one of the patients on the steering committee.

*Statistical analysis:*

Data were treated as continuous variables wherever possible to maximize power. All normally distributed data are presented as mean ± standard deviation (SD). Skewed data were appropriately transformed and presented as geometric means (with 95% confidence intervals [CI]). Statistical significance for categorical variables was calculated using the chi-squared test and the Student's t-test for continuous variables. Independence of ACR (as a risk predictor) from diabetes and other measures of the ABCD2 score was assessed using logistic regression. Microalbuminuria was defined


For peer review only - http://bmjopen.bmj.com/site/about/guidelines.xhtml

using the currently accepted clinical thresholds for microalbuminuria used in people with diabetes 3.5mg/mol for women and 2.5mg/mol for men. Prediction of events were calculated using a Cox proportional hazard ratio with time to first MACE as the primary outcome. In keeping with the recommendations of Cupples[27] and Rothman[28] the measured significance of the variables of interest is reported without adjustment for multiple testing. Statistical significance was considered at $p<0.05$. Statistical analysis was performed using Stata SE 14.2 (Mac version: Statacorp ltd Texas).

**Results:**

Two thousand, four hundred and eight patients with a diagnosis of definite or probable minor stroke/TIA were recruited; 149 participants were subsequently excluded after the diagnosis was revised to that of a stroke mimic, 8 withdrew consent and a further 49 (2%) were excluded due to intercurrent urinary tract infection rendering the assessment of ACR invalid. No patient was lost to follow-up. Baseline characteristics are presented in table 1. Of the 2,202 included in the final analysis, most were male (64.9%). All patients were commenced on secondary prevention with appropriate antiplatelet or anticoagulant therapy, and an appropriately dosed statin in accordance with contemporaneous national guidelines.

Over 90 days, 151 primary outcome events (MACE) occurred in 144 participants (6.7% of patients) including 8 cardiovascular deaths. There were also 8 non-cardiovascular deaths. Within 7 days there were 38 MACE in 36 participants and a further 76 events that required hopitalisation, predominantly carotid endarterectomy. Those with MACE were more likely to have had previous stroke/TIA but were otherwise similar to those without recurrence (Table 1). There was no difference in mean ABCD2 score or the

For peer review only

proportion of participants with a 'high risk' score (4/7 or more) between those who did and did not have events by 90 days. When looking exclusively at the 1374 participants identified with a "high risk" ABCD2 score (63% of the study sample; 93% male), a high score did not distinguish those at high risk of MACE event compared to those with an ABCD2 <4 (HR 1.15 (0.82-1.63); p=0.4), nor did a low risk score exclude an event, with only 37.7% of people who did not have experience a MACE outcome scoring below 4. Point-of-care ACR was higher in those participants with a primary outcome event at 90 days compared to those without (2.27 (1.92-2.68) vs. 1.75 (1.68–1.83) mg/mmol respectively; p=0.002). This distinction remained after adjustment for age and sex (adjusted ACR 2.40 (2.04-2.83) vs. 1.88 (1.80 – 1.96), p=0.004. Figure 1).

*Evaluation of the prognostic role of microalbuminuria alone*

Fractional polynomial regression modelling identified the sex-specific threshold of risk for ACR aligned with the currently accepted thresholds for microalbuminuria used in people with diabetes of 2.5mg/mmol for men and 3.5mg/mol for women. These thresholds identified 562 participants (25.4% of the study sample; 75% male) with microalbuminuria. These participants were more likely than those with a low ACR to experience a primary outcome event (Hazard Ratio 1.66 (95% CI 1.19 - 2.34); p=0.003) and had a greater than 5-fold increase in 90-day mortality (HR 5.44 (1.86 – 15.90); p=0.0003). Alone, however, microalbuminuria did not have sufficient positive or negative predictive value to justify its clinical utility (Positive predictive value – 38.2%, negative predictive value 73.8%).

*Generating a composite risk score for 90-day risk stratification*



For peer review only - http://bmjopen.bmj.com/site/about/guidelines.xhtml

In a stepwise multivariate model, the only independent predictors of MACE were elevated ACR, age and history of previous stroke or TIA. Similar variance within the model was explained microalbuminuria (i.e. an ACR >2.5 and 3.5mg/mol respectively in men and women) as being older than 80 years and previous history of stroke/TIA, so they were afforded equal weighting. This generated a 4-point scale between 0 and 3 (termed the 'APA' score representing Age, Prior stroke or TIA and elevated ACR), with the lowest risk participants having a score of 0 (Table 2). There was a sequential increase in risk with increasing score such that those with a score of 0 having a 90-day risk of 4.86% rising to a 30% risk for those with the highest score (supplementary table 1). The population was then divided into a low risk group scoring 0 or 1 or high risk scoring 2 or 3. 1,985 (90.1%) of participants were identified as low risk compared to 217 (9.9%) in the high-risk group. The high-risk group was older, with higher systolic blood pressure and pulse pressure compared to the low-risk group (table 3).

The 90-day risk of a MACE outcome was 5.7% in the low-risk group, versus 12.4% in the high-risk group (p<0.001), translating to a hazard ratio of 2.12 (95% CI 1.38 – 3.25) for the high risk score compared to low-risk group (p<0.001; Figure 2). This was predominantly driven by an increase in recurrent stroke/TIA (3.3% vs. 8.8%; HR 2.67(1.58-4.51); p<0.001). This difference was apparent within 7 days of the clinic, such that those with a high APA score had a 2.2-fold increased risk of a recurrent event.

*Validation of APA score on a historic sample*

In order to validate the APA score, we applied the tool to the individuals who participated in the pilot study but did not contribute their data to this dataset. Details of this pilot study have been published elsewhere [24]. In brief, the pilot study recruited 139



For peer review only - http://bmjopen.bmj.com/site/about/guidelines.xhtml

participants over 9 months from one of the centres involved in the definitive study. In this population, there were 13 recurrent events (9.35%) within the 90 days, 9 of which occurred in the first 7 days after attendance to the stroke clinic (Table 4). The APA risk score replicated a similar distribution of number of participants at low risk (73%) and high risk (27%) as in the larger study. At 90 days, 5.9% of the low APA group had recurrent events compared to 18.4% of the in the high-APA group (p=0.02). Again, within the first 7 days this was apparent with only 1.9% of the low APA group experiencing the primary outcome, compared to 13.2% in the high APA group (p=0.04).

**Discussion:**

In this large UK multicentre prospective study with blinded outcome adjudication, we have demonstrated the utility of an elevated ACR, measured using a simple point-of-care analyser, in combination with simple clinical data (age and previous history of stroke or TIA) in the prediction of major adverse cardiovascular events and death. This differentiates between low and high-risk individuals. Specifically, over 90% of individuals were identified as low risk with less than a 1.5% risk of a major adverse cardiovascular event or death over the next 7 days, increasing to 5.7% by 90 days, whereas 9.9 % of the population were identified at high risk with a 12.4% event rate over 90 days. Although only a small number, our findings were validated in a separate historic population who participated in the pilot study.

The utility of APA scoring may go beyond simply guiding conversations regarding prognosis in a clinic setting. The POINT[8] and CHANCE[9] studies demonstrated the benefit of dual anti-platelet therapy in the first 21 days after high risk TIA. There was, however, a 0.5% absolute increase in major intracranial haemorrhage. We would


For peer review only - http://bmjopen.bmj.com/site/about/guidelines.xhtml

propose that an assessment of the APA score would be appropriate prior to initiating therapy. In those with a low risk APA score, even the 25% relative risk reduction in the POINT trial would result in only a marginally beneficial risk:benefit ratio. Those with a high APA score, however, would likely benefit from dual-antiplatelet therapy with a number need to treat of less than 30 in order to prevent an event. The APA score also gives additional information that is of use in the more acute setting. Over the first 7 days, a low-risk APA score had a negative predictive value of >98.5% for the risk of a MACE - a number very similar to the predictive effect of troponin when evaluating suspected cardiac chest pain.[29]

When considering the populations identified by the APA score, there are similarities to the ABCD2 score. Indeed, the demographics of the high-risk group were older, with higher blood pressure and a trend towards more diabetes. We would suggest, that microalbuminuria represents vascular susceptibility to the adverse consequences of hypertension and diabetes rather than simply an indication of the presence of hypertension and /or diabetes in general. This would explain why the APA score predicted further MACE and total mortality at 7 and 90 days, whereas in our large sample the ABCD2 score did not.

Elevated ACR is a recognised marker of generalised endothelial and microvascular dysfunction. The increased filtration of albumin through the renal glomerular filtration barrier is thought to be due to changes in the chemical and physical properties of this endothelial barrier and its glycocalyx.[30-32] The mechanism explaining the association between microalbuminuria and incident cardiovascular events is thought to depend on its role as a marker of systemically increased vascular permeability and altered


For peer review only - http://bmjopen.bmj.com/site/about/guidelines.xhtml

homeostasis, coagulation and endothelial function.[33-38] However, after acute ischaemic events such as myocardial infarction, or as demonstrated here TIA or minor stroke, it is likely that elevated urinary ACR is, at least in part, dependent on the systemic inflammatory response to the original insult.

Whilst unlikely to be mechanistically involved in the progression of disease, the question as to whether it is a good surrogate of therapeutic effect remains. Improving systemic inflammation, such as through the pleiotropic effect of statins, has been associated with simultaneous improvements in urinary albumin excretion and CV event rates in patients with elevated high sensitivity C-reactive protein (hsCRP).[39-41] Further studies are needed to determine whether microalbuminuria after acute stroke represents a therapeutic target amenable to treatment, and if reductions in microalbuminuria are associated with reductions in the vascular events they predict.

*Limitations and strengths of study*

Recurrent event rate was lower than anticipated from our pilot study and contemporaneous clinical trials such as CHANCE (11.7% in the control arm),[9] although are comparable with the recently-reported randomised controlled POINT trial (6%).[8] In usual practice it is usually 24-48 hours between the index event and clinic attendance. Given that data from previous studies has demonstrated the highest risk occurs in the first 48 hours, it is likely that some events will have occurred between the index event and attendance in the clinic in our study.[42] The pragmatic design of the study, however, is also a strength given that the predictive role highlights those that may benefit from dual anti-platelet therapy or hospitalisation when the patient is seen in the acute stroke clinic.


For peer review only - http://bmjopen.bmj.com/site/about/guidelines.xhtml

There is no consensus on the gold standard for diagnosing TIA in a rapid access stroke clinic.[43] In order to maintain the generalisability of the study, all attendees of the stroke clinic that were diagnosed with a probable or definite TIA were invited to participate. Only a small number of patients were subsequently withdrawn as being stroke mimics. Outcome events were rigorously adjudicated against published diagnostic criteria to ensure consistency and reliability.

Finally, the use of a single urinary ACR sample obtained during the clinic assessment using a near patient testing kit is not as robust as a sample tested in a central laboratory. Whilst there is significant diurnal variability in urinary AER within individuals, potentially limiting the scientific or mechanistic merit of the study compared to studies using overnight or 24-hour urine collection, the pragmatic design makes this study more applicable to general clinic populations. Further, the relatively low cost of the near patient test kit makes it accessible in most emergency settings, in primary and secondary care.

**Conclusion:**

We have demonstrated for the first time the potential utility of a single point of care test of urinary ACR in patients presenting with TIA and minor stroke as a prediction tool to assist triaging patients. When used in combination with patient age and previous history of stroke or TIA, this generated a risk stratification score (the APA score: Age>80, Past history of stroke/TIA, and elevated ACR) that could reliably identify the large proportion of patients with a low risk of recurrent vascular event over the next 90 days. The value of ACR that is most predictive is aligned with the conventional


For peer review only - http://bmjopen.bmj.com/site/about/guidelines.xhtml

definition of microalbuminuria used in diabetes, suggesting that in clinical practice the existing technology for evaluating microalbuminuria including urine dipsticks may be used. A high APA score was associated with a doubling of risk of MACE outcome and a five fold increase in mortality.  This APA score was validated in an independent population. We believe the APA score may represent a practical tool for clinicians engaged in the acute ambulatory assessment of TIA and minor stroke, to assist in the assessment of risk:benefit ratio for the use dual anti-platelet therapy or to admit for more urgent investigation. Further work is required to determine whether the increase in ACR represents a therapeutic target or solely a prognostic indicator.

For peer review only

**Perspectives:**

***Competency in Medical Knowledge:*** Stroke is a leading cause of morbidity and mortality worldwide. TIAs and minor strokes are heralds for a major stroke in up to 10% of those patients. The appropriate detection and risk stratification of high-risk patients who are at risk of progression to a completed stroke is an important step in avoiding this catastrophic outcome with its subsequent implications on the healthcare and social services.

Currently available TIA risk stratification lack sufficient positive and negative predictive value for clinical utility.

Dual anti-platelet therapy has been demonstrated to be superior to a single agent after high risk TIAs, however the risk of haemorrhage is significant and identifying the population with most to gain remains a goal

***Translational outlook:*** Microalbuminuria is a marker of cardiovascular disease and predicts cardiovascular events and mortality in the general population and in patients with cardiovascular risk factors. Urinary ACR is a reliable marker of microalbuminuria. Compared to low risk patients we demonstrated an elevated ACR in TIA and minor stroke patients who proceeded to have further events within 90 days.

***Translational Outlook:*** Incorporating microalbuminuria with some basic demographic data can identify 90% of the population with a low risk of recurrent event (98.5% negative predictive value at 7 days), compared to a subgroup of 10% who had 12.4% risk of a MACE event.



For peer review only - http://bmjopen.bmj.com/site/about/guidelines.xhtml

For peer review only

**Acknowledgements:**

This study was funded by a NIHR Research for Patient Benefit grant (PB-PG-1112-29069) and supported by the NIHR Exeter Clinical Research Facility. MAJ is supported by the NIHR SW Peninsula Applied Research Collaboration. This manuscript does not necessarily reflect the views of the NIHR, the Exeter Clinical Research Facility, the NHS or the UK Department of Health.

For peer review only

**References:**


1. Feigin VL, Norrving B, Mensah GA. Global Burden of Stroke. *Circ Res.* 2017;120(3):439-448.
2. Henssge U. National Sentinel Stroke Clinical Audit 2010. In: Physicians RCo, ed2011.
3. Burn J, Dennis M, Bamford J, Sandercock P, Wade D, Warlow C. Long-term risk of recurrent stroke after a first-ever stroke. The Oxfordshire Community Stroke Project. *Stroke; a journal of cerebral circulation.* 1994;25(2):333-337.
4. Hankey GJ. Long-term outcome after ischaemic stroke/transient ischaemic attack. *Cerebrovascular diseases (Basel, Switzerland).* 2003;16 Suppl 1:14-19.
5. Rothwell PM, Warlow CP. Timing of TIAs preceding stroke: time window for prevention is very short. *Neurology.* 2005;64(5):817-820.
6. Johnston SC, Gress DR, Browner WS, Sidney S. Short-term prognosis after emergency department diagnosis of TIA. *JAMA.* 2000;284(22):2901-2906.
7. Hill MD, Yiannakoulias N, Jeerakathil T, Tu JV, Svenson LW, Schopflocher DP. The high risk of stroke immediately after transient ischemic attack: a population-based study. *Neurology.* 2004;62(11):2015-2020.
8. Johnston SC, Easton JD, Farrant M, et al. Clopidogrel and Aspirin in Acute Ischemic Stroke and High-Risk TIA. *N Engl J Med.* 2018;379(3):215-225.
9. Wang Y, Wang Y, Zhao X, et al. Clopidogrel with aspirin in acute minor stroke or transient ischemic attack. *N Engl J Med.* 2013;369(1):11-19.
10. Kiyohara T, Kamouchi M, Kumai Y, et al. ABCD3 and ABCD3-I scores are superior to ABCD2 score in the prediction of short- and long-term risks of stroke after transient ischemic attack. *Stroke; a journal of cerebral circulation.* 2014;45(2):418-425.


For peer review only



For peer review only - http://bmjopen.bmj.com/site/about/guidelines.xhtml

11. Rothwell PM, Giles MF, Flossmann E, et al. A simple score (ABCD) to identify individuals at high early risk of stroke after transient ischaemic attack. *Lancet.* 2005;366(9479):29-36.

12. Bhatt A, Jani V. The ABCD and ABCD2 Scores and the Risk of Stroke following a TIA: A Narrative Review. *ISRN Neurol.* 2011;2011:518621.

13. Ay H, Arsava EM, Johnston SC, et al. Clinical- and imaging-based prediction of stroke risk after transient ischemic attack: the CIP model. *Stroke; a journal of cerebral circulation.* 2009;40(1):181-186.

14. Giles MF, Rothwell PM. Systematic review and pooled analysis of published and unpublished validations of the ABCD and ABCD2 transient ischemic attack risk scores. *Stroke; a journal of cerebral circulation.* 2010;41(4):667-673.

15. Amarenco P, Labreuche J, Lavallée PC. Patients With Transient Ischemic Attack With ABCD2 <4 Can Have Similar 90-Day Stroke Risk as Patients With Transient Ischemic Attack With ABCD2 ≥4. *Stroke; a journal of cerebral circulation.* 2012;43(3):863-865.

16. Amarenco P, Labreuche J, Lavallee PC. Patients with transient ischemic attack with ABCD2 <4 can have similar 90-day stroke risk as patients with transient ischemic attack with ABCD2 >/=4. *Stroke; a journal of cerebral circulation.* 2012;43(3):863-865.

17. National Institute of Healthcare Excellence. Stroke and transient ischaemic attack in over 16s: diagnosis and initial management; NICE guideline [NG128]. National Institute of Health and Care Excellence. Published 2019. Updated May 2019. Accessed 10/11/19, 2019.

18. Powers WJ, Rabinstein AA, Ackerson T, et al. 2018 Guidelines for the Early Management of Patients With Acute Ischemic Stroke: A Guideline for

For peer review only



For peer review only - http://bmjopen.bmj.com/site/about/guidelines.xhtml

Healthcare Professionals From the American Heart Association/American Stroke Association. *Stroke.* 2018;49(3):e46-e110.

19. Ahmed N, Audebert H, Turc G, et al. Consensus statements and recommendations from the ESO-Karolinska Stroke Update Conference, Stockholm 11–13 November 2018. *European Stroke Journal.*0(0):2396987319863606.
20. Gerstein HC, Mann JF, Yi Q, et al. Albuminuria and risk of cardiovascular events, death, and heart failure in diabetic and nondiabetic individuals. *JAMA.* 2001;286(4):421-426.
21. Hillege HL, Fidler V, Diercks GF, et al. Urinary albumin excretion predicts cardiovascular and noncardiovascular mortality in general population. *Circulation.* 2002;106(14):1777-1782.
22. Yuyun MF, Khaw KT, Luben R, et al. Microalbuminuria, cardiovascular risk factors and cardiovascular morbidity in a British population: the EPIC-Norfolk population-based study. *Eur J Cardiovasc Prev Rehabil.* 2004;11(3):207-213.
23. Strain WD, Shore AC, Melzer D. Albumin:creatinine ratio predicts mortality after stroke: analysis of the Third National Health and Nutrition Examination Survey. *J Am Geriatr Soc.* 2010;58(12):2434-2435.
24. Elyas S, Shore AC, Kingwell H, et al. Microalbuminuria could improve risk stratification in patients with TIA and minor stroke. *Ann Clin Transl Neurol.* 2016;3(9):678-683.
25. Morgenstern LB, Fox AJ, Sharpe BL, Eliasziw M, Barnett HJ, Grotta JC. The risks and benefits of carotid endarterectomy in patients with near occlusion of the carotid artery. North American Symptomatic Carotid Endarterectomy Trial (NASCET) Group. *Neurology.* 1997;48(4):911-915.



For peer review only - http://bmjopen.bmj.com/site/about/guidelines.xhtml

26. Kvam C. Afinion ACR Scientific Poster AACC 2007. Axis-Shield PoC AS. http://www.afinion.net/publications. Published 2007. Accessed.

27. Cupples LA, Heeren T, Schatzkin A, Colton T. Multiple testing of hypotheses in comparing two groups. *Ann Intern Med.* 1984;100(1):122-129.

28. Rothman KJ. No adjustments are needed for multiple comparisons. *Epidemiology.* 1990;1(1):43-46.

29. Gualandro DM, Puelacher C, LuratiBuse G, et al. Comparison of high-sensitivity cardiac troponin I and T for the prediction of cardiac complications after non-cardiac surgery. *Am Heart J.* 2018;203:67-73.

30. Haraldsson B, Jeansson M. Glomerular filtration barrier. *Curr Opin Nephrol Hypertens.* 2009;18(4):331-335.

31. Singh A, Satchell SC. Microalbuminuria: causes and implications. *Pediatr Nephrol.* 2011;26(11):1957-1965.

32. Gansevoort RT, Lambers H, Witte EC. Methodology of screening for albuminuria. *Nephrology Dialysis Transplantation.* 2007;22(8):2109-2111.

33. Barzilay JI, Peterson D, Cushman M, et al. The relationship of cardiovascular risk factors to microalbuminuria in older adults with or without diabetes mellitus or hypertension: the cardiovascular health study. *Am J Kidney Dis.* 2004;44(1):25-34.

34. Valmadrid CT, Klein R, Moss SE, Klein BE. The risk of cardiovascular disease mortality associated with microalbuminuria and gross proteinuria in persons with older-onset diabetes mellitus. *Arch Intern Med.* 2000;160(8):1093-1100.

35. Dinneen SF, Gerstein HC. The association of microalbuminuria and mortality in non-insulin-dependent diabetes mellitus. A systematic overview of the literature. *Arch Intern Med.* 1997;157(13):1413-1418.



For peer review only - http://bmjopen.bmj.com/site/about/guidelines.xhtml

36. Festa A, D'Agostino R, Howard G, Mykkanen L, Tracy RP, Haffner SM. Inflammation and microalbuminuria in nondiabetic and type 2 diabetic subjects: The Insulin Resistance Atherosclerosis Study. *Kidney Int.* 2000;58(4):1703-1710.

37. Asselbergs FW, de Boer RA, Diercks GF, et al. Vascular endothelial growth factor: the link between cardiovascular risk factors and microalbuminuria? *Int J Cardiol.* 2004;93(2-3):211-215.

38. Stehouwer CD, Gall MA, Twisk JW, Knudsen E, Emeis JJ, Parving HH. Increased urinary albumin excretion, endothelial dysfunction, and chronic low-grade inflammation in type 2 diabetes: progressive, interrelated, and independently associated with risk of death. *Diabetes.* 2002;51(4):1157-1165.

39. Ridker PM, Danielson E, Fonseca FA, et al. Rosuvastatin to prevent vascular events in men and women with elevated C-reactive protein. *N Engl J Med.* 2008;359(21):2195-2207.

40. Abe M, Maruyama N, Okada K, Matsumoto S, Matsumoto K, Soma M. Effects of lipid-lowering therapy with rosuvastatin on kidney function and oxidative stress in patients with diabetic nephropathy. *J Atheroscler Thromb.* 2011;18(11):1018-1028.

41. Girardi JM, Farias RE, Ferreira AP, Raposo NR. Rosuvastatin prevents proteinuria and renal inflammation in nitric oxide-deficient rats. *Clinics (Sao Paulo).* 2011;66(8):1457-1462.

42. Coull AJ, Rothwell PM. Underestimation of the early risk of recurrent stroke: evidence of the need for a standard definition. *Stroke; a journal of cerebral circulation.* 2004;35(8):1925-1929.



For peer review only - http://bmjopen.bmj.com/site/about/guidelines.xhtml

43. Graham C, Bailey D, Hart S, et al. Clinical diagnosis of TIA or minor stroke and prognosis in patients with neurological symptoms: A rapid access clinic cohort. *PLoS One*. 2019;14(3):e0210452.

For peer review only

**Table Legends (ProMOTE Study):**

**Table 1**: Sample characteristics of total population and population stratified by occurrence of recurrent major adverse cardiovascular events (MACE) at 90 days

Figures presented as mean (SD) for continuous variable except ACR where geometric mean (Inter-quartile range) presented; % for categorical data

P for continuous data by Student's t-test

P for categorical data by Chi Squared test

BMI = Body Mass Index,

ABCD2 (A=Age, B=BP, C=Clinical features, D= Duration of symptoms + Diabetes),

ACR Albumin Excretion Rate

*Note*: Total Cholesterol was collected from routine clinical practice records therefore is not available for all participants

**Table 2**: APA (Age, Previous Stroke and ACR) Score derivation

**Table 3**: Occurrence of events by APA risk score group (mean ± SD for continuous variable, p for ANOVA; % for categorical data, p for Chi-Squared)

**Table 4:** Occurrence of events by APA risk score group in independent validation population (mean ± SD for continuous variable, p for ANOVA; % for categorical data, p for Chi-Squared)



For peer review only - http://bmjopen.bmj.com/site/about/guidelines.xhtml

For peer review only

Table 1: Sample characteristics of total population and population stratified by occurrence of recurrent vascular events or death (MACE) at 90 days.

| Characteristic | Total population | No event by 90 days | Event at 90 days |
|---|---|---|---|
| N | 2202 | 2058 | 144 |
| Age (years) | 70.9 (11.7) | 70.9 (11.6) | 72.2 (12.2) |
| Sex (%male) | 64.8 | 64.7 | 67.9 |
| Height (m) | 1.70 (0.10) | 1.70 (0.10) | 1.71 (0.9) |
| Weight (kg) | 79.8 (17.1) | 79.9 (17.0) | 79.7 (17.7) |
| BMI (kg/$m^2$) | 27.6 (5.2) | 27.6 (5.2) | 27.2 (4.9) |
| Blood pressure | | | |
| Systolic (mmHg) | 144.1 (20.8) | 144.2 (20.9) | 145.7 (21.7) |
| Diastolic (mmHg) | 81.8 (12.6) | 81.7 (12.6) | 83.8 (13.1) |
| Total Cholesterol§ | (n = 1,466) | (n=1,374) | (n=92) |
| (mmol/L) | 5.02 (2.57) 194 | 5.03 (2.63) | 4.75 (1.24) |
| (mg/dl) | (99) | 194 (102) | 183 (48) |
| Diabetes (%) | 15.7 | 15.9 | 12.1 |
| Previous Stroke (%) | 4.0 | 3.5 | 10.7* |
| ABCD2 | | | |
| Mean score out of 7 | 4.0 (1.4) | 4.0 (1.4) | 4.1 (1.4) |
| Proportion >4 (%) | 62.5 | 62.3 | 65.0 |
| "High Risk" | | | |
| ACR (mg/mmol)† | 1.79(0.9-2.9) | 1.75(1.69-1.83) | 2.27(1.92-2.68)** |
| ACR (mg/g)† | 15.8(8.0-25.6) | 15.5 (14.9-16.2) | 20.1 (17.0-23.7)** |

§ These were collected from patient records where clinician had requested the test and therefore only available in a subset of the population

† Geometric mean (95% CI)

*P<0.0001 compared to those with no events

** p=0.002 compared to those with no events

For peer review only

**Table 2: APA (Age, Previous Stroke and ACR) Score derivation**

| | Variable | Value |
|---|---|---|
| **Age** | ≥80 years | 1 |
| **Previous stroke or TIA** | Present | 1 |
| **ACR** | ≥3.5 mg/mmol for women<br>≥2.5 mg/mol for men | 1 |

| | | |
|---|---|---|
| **Score** | 0-1 | 2-3 |
| **Risk** | Low Risk | High Risk |

ACR: Albumin Creatinine Ratio

For peer review only

Table 3: Occurrence of events by APA risk score group (mean ± SD for continuous variable, p for ANOVA; % for categorical data, p for Chi-Squared)

| | Low Risk | High Risk | p |
|---|---|---|---|
| n | 1,985 | 217 | |
| Sex (% Male) | 64.5 | 68.2 | 0.2 |
| Age (years) | 69.6 (11.3) | 84.0 (5.0) | <0.0001 |
| Blood pressure (mmHg) | | | |
| Systolic | 143 (20.4) | 150.5 (23.3) | <0.0001 |
| Diastolic | 82 (12.3) | 79 (11.6) | 0.0007 |
| Weight (kg) | 80.3 (17.1) | 75.1 (16.2) | <0.0001 |
| Diabetes | 15.3 | 18.9 | 0.2 |
| MACE at 90 days (%) | | | |
| All events | 5.7 | 12.4 | <0.0001 |
| Cerebrovascular event | 3.2 | 8.8 | <0.0001 |
| Cardiovascular Mortality | 0.1 | 1.4 | <0.001 |
| All Cause Mortality | 0.4 | 2.3 | 0.001 |
| MACE at 7 days (%) | 1.46 | 3.23 | 0.05 |

For peer review only

**Table 4**: Occurrence of events by APA risk score group in independent validation population (mean ± SD for continuous variable, p for ANOVA; % for categorical data, p for Chi-Squared)

| | Low Risk | High Risk | p |
|---|---|---|---|
| n | 101 | 38 | |
| Sex (% Male) | 51.5 | 71.5 | 0.04 |
| Age (years) | 72.6 (10.7) | 81.6 (6.1) | 0.001 |
| Blood pressure (mmHg) | | | |
| Systolic | 142.6 (20.2) | 147.3 (26.6) | 0.3 |
| Diastolic | 77.7 (9.8) | 77.4 (10.8) | 0.9 |
| Weight (kg) | 76.2 (16.4) | 75.0 (15.4) | 0.7 |
| Recurrent event at 90 days (%) | | | |
| MACE event | 5.9 | 18.4 | 0.02 |
| Recurrent event at 7 days (%) | | | |
| MACE events* | 1.9 | 13.2 | 0.04 |

* MACE = Major Adverse Cardiovascular Event – Recurrent stroke or TIA, Myocardial infarction or cardiovascular death.

For peer review only

**Figure 1:** Age and sex adjusted Albumin Creatinine Ratio (Mean +/-95% CI) stratified by the occurrence of a Major Adverse Cardiac Event (MACE) in the first 90 days post initial event

MACE – Major Adverse Cardiovascular event = recurrent cerebrovascular event, myocardial infarction or cardiovascular death

**Figure 2**: Time to first Major Adverse Cardiac Event (MACE) stratified by APA (Age, Previous cerebrovascular event, High ACR) risk score. Event rate in high risk (n=217) 12.44% vs. low risk (n=1985) 5.69%, HR 2.12 (1.38 – 3.25) p<0.001

For peer review only

For peer review only



For peer review only - http://bmjopen.bmj.com/site/about/guidelines.xhtml

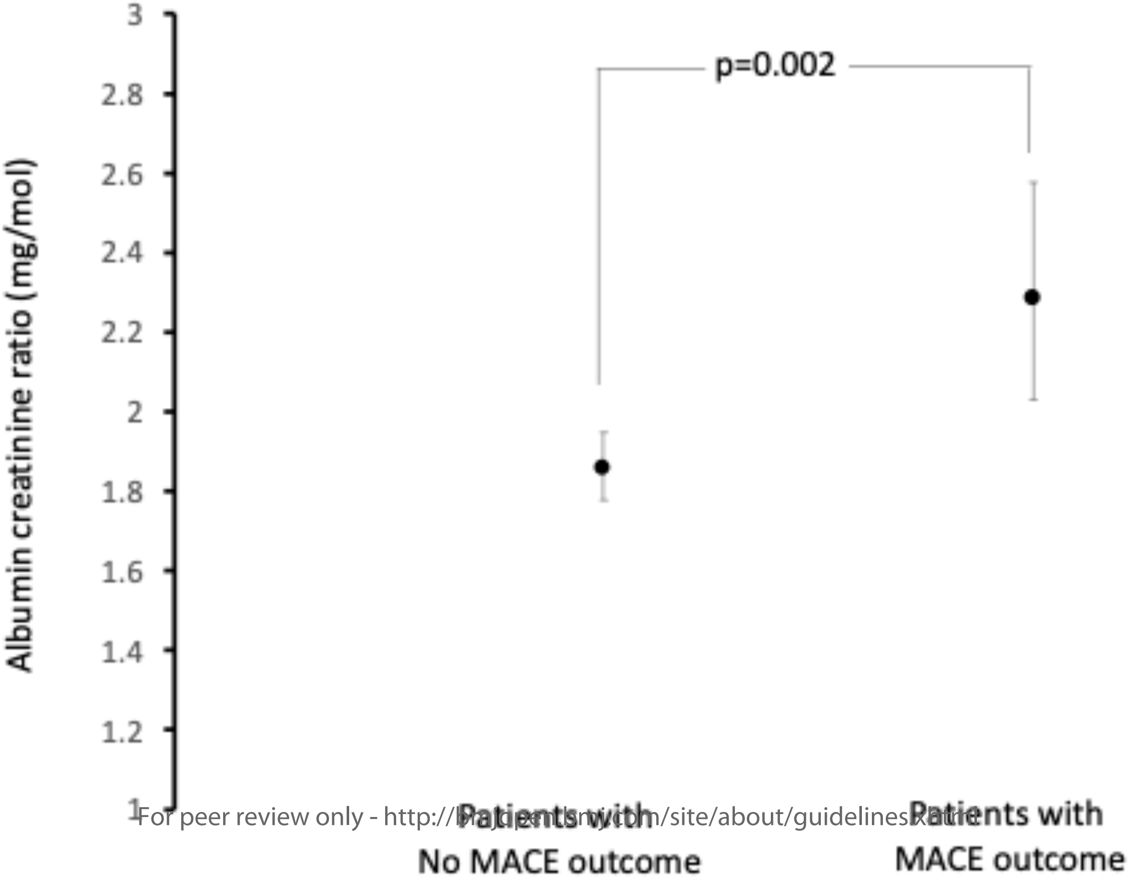
Albumin creatinine ratio (mg/mol)
3
2.8
2.6
2.4
2.2
2
1.8
1.6
1.4
1.2
1
p=0.002
Patients with
No MACE outcome
Patients with
MACE outcome

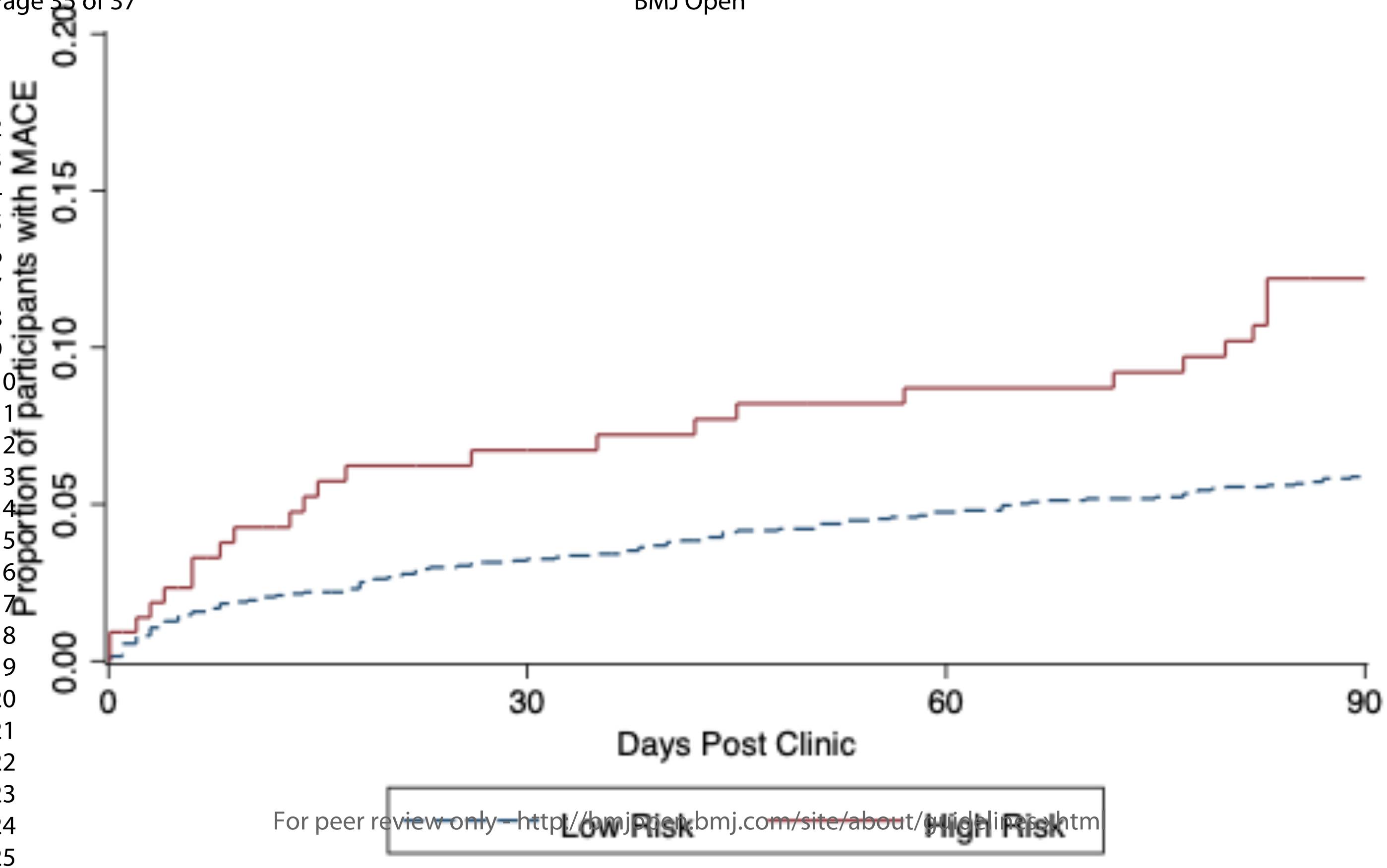

Proportion of participants with MACE
0.00
0.05
0.10
0.15
0.20
0
30
60
90
Days Post Clinic
Low Risk
High Risk

Supplementary Table 1: Occurrence of major adverse cardiac events (MACE) by APA risk score group (p for Chi-Squared)

| APA score | N | Occurrence of MACE events (%) |
|---|---|---|
| **0** | 1215 | 4.9 |
| **1** | 770 | 7.0 |
| **2** | 207 | 11.6 |
| **3** | 10 | 30 |
| | | P<0.001 |

For peer review only

STROBE Statement—checklist of items that should be included in reports of observational studies

| | Item No | Recommendation | Page number |
|---|---|---|---|
| **Title and abstract** | 1 | (*a*) Indicate the study's design with a commonly used term in the title or the abstract | 1 |
| | | (*b*) Provide in the abstract an informative and balanced summary of what was done and what was found | 2 |
| **Introduction** | | | |
| Background/rationale | 2 | Explain the scientific background and rationale for the investigation being reported | 4 |
| Objectives | 3 | State specific objectives, including any prespecified hypotheses | 5 |
| **Methods** | | | |
| Study design | 4 | Present key elements of study design early in the paper | 6 |
| Setting | 5 | Describe the setting, locations, and relevant dates, including periods of recruitment, exposure, follow-up, and data collection | 5 |
| Participants | 6 | (*a*) *Cohort study*—Give the eligibility criteria, and the sources and methods of selection of participants. Describe methods of follow-up<br>*Case-control study*—Give the eligibility criteria, and the sources and methods of case ascertainment and control selection. Give the rationale for the choice of cases and controls<br>*Cross-sectional study*—Give the eligibility criteria, and the sources and methods of selection of participants | 6 |
| | | (*b*) *Cohort study*—For matched studies, give matching criteria and number of exposed and unexposed<br>*Case-control study*—For matched studies, give matching criteria and the number of controls per case | |
| Variables | 7 | Clearly define all outcomes, exposures, predictors, potential confounders, and effect modifiers. Give diagnostic criteria, if applicable | 6-7 |
| Data sources/ measurement | 8* | For each variable of interest, give sources of data and details of methods of assessment (measurement). Describe comparability of assessment methods if there is more than one group | *8* |
| Bias | 9 | Describe any efforts to address potential sources of bias | 8 |
| Study size | 10 | Explain how the study size was arrived at | 8 |
| Quantitative variables | 11 | Explain how quantitative variables were handled in the analyses. If applicable, describe which groupings were chosen and why | 8 |
| Statistical methods | 12 | (*a*) Describe all statistical methods, including those used to control for confounding | 8 |
| | | (*b*) Describe any methods used to examine subgroups and interactions | 8 |
| | | (*c*) Explain how missing data were addressed | N/A |
| | | (*d*) *Cohort study*—If applicable, explain how loss to follow-up was addressed<br>*Case-control study*—If applicable, explain how matching of cases and controls was addressed<br>*Cross-sectional study*—If applicable, describe analytical methods taking account of sampling strategy | |
| | | (*e*) Describe any sensitivity analyses | 8 |

Continued on next page



For peer review only - http://bmjopen.bmj.com/site/about/guidelines.xhtml

For peer review only

| | | | |
|---|---|---|---|
| **Results** | | | |
| Participants | 13* | (a) Report numbers of individuals at each stage of study—eg numbers potentially eligible, examined for eligibility, confirmed eligible, included in the study, completing follow-up, and analysed | 9 |
| | | (b) Give reasons for non-participation at each stage | N/A |
| | | (c) Consider use of a flow diagram | N/A |
| Descriptive data | 14* | (a) Give characteristics of study participants (eg demographic, clinical, social) and information on exposures and potential confounders | 8 |
| | | (b) Indicate number of participants with missing data for each variable of interest | 8 |
| | | (c) *Cohort study*—Summarise follow-up time (eg, average and total amount) | 8 |
| Outcome data | 15* | *Cohort study*—Report numbers of outcome events or summary measures over time | 9 |
| | | *Case-control study*—Report numbers in each exposure category, or summary measures of exposure | |
| | | *Cross-sectional study*—Report numbers of outcome events or summary measures | |
| Main results | 16 | (*a*) Give unadjusted estimates and, if applicable, confounder-adjusted estimates and their precision (eg, 95% confidence interval). Make clear which confounders were adjusted for and why they were included | 9, 26 |
| | | (*b*) Report category boundaries when continuous variables were categorized | |
| | | (*c*) If relevant, consider translating estimates of relative risk into absolute risk for a meaningful time period | |
| Other analyses | 17 | Report other analyses done—eg analyses of subgroups and interactions, and sensitivity analyses | 10,11 |
| **Discussion** | | | |
| Key results | 18 | Summarise key results with reference to study objectives | 11 |
| Limitations | 19 | Discuss limitations of the study, taking into account sources of potential bias or imprecision. Discuss both direction and magnitude of any potential bias | 14 |
| Interpretation | 20 | Give a cautious overall interpretation of results considering objectives, limitations, multiplicity of analyses, results from similar studies, and other relevant evidence | 13 |
| Generalisability | 21 | Discuss the generalisability (external validity) of the study results | 14 |
| **Other information** | | | |
| Funding | 22 | Give the source of funding and the role of the funders for the present study and, if applicable, for the original study on which the present article is based | 17 |

*Give information separately for cases and controls in case-control studies and, if applicable, for exposed and unexposed groups in cohort and cross-sectional studies.

**Note:** An Explanation and Elaboration article discusses each checklist item and gives methodological background and published examples of transparent reporting. The STROBE checklist is best used in conjunction with this article (freely available on the Web sites of PLoS Medicine at http://www.plosmedicine.org/, Annals of Internal Medicine at http://www.annals.org/, and Epidemiology at http://www.epidem.com/). Information on the STROBE Initiative is available at www.strobe-statement.org.

For peer review only

BMJ Open

# The evaluation of microalbuminuria as a prognostic indicator after a TIA or minor stroke in an outpatient setting: The Prognostic Role of Microalbuminuria in TIA Evolution (ProMOTE) Study

| | |
|---|---|
| Journal: | *BMJ Open* |
| Manuscript ID | bmjopen-2020-043253.R1 |
| Article Type: | Original research |
| Date Submitted by the Author: | 07-Apr-2021 |
| Complete List of Authors: | Strain, W; University of Exeter, Diabetes and Vascular Research Centre; Royal Devon and Exeter NHS Foundation Trust, Academic Department of Healthcare for Older People<br>Elyas, Salim; University of Exeter, Diabetes and Vascular Research Centre; Royal Devon and Exeter NHS Foundation Trust, Academic Department of Healthcare for Older People<br>Wedge, Nicola; Royal Devon and Exeter NHS Foundation Trust, Academic Department of Healthcare for Older People<br>Mounce, Luke; University of Exeter, Institute of Health Research<br>Henley, W; University of Exeter Medical School, Institute of Health Research<br>James, Martin; Royal Devon and Exeter NHS Foundation Trust, Academic Department of Healthcare for Older People<br>Shore, Angela; University of Exeter Medical School, NIHR Exeter Clinical Research Facility |
| <b>Primary Subject Heading</b>: | Medical management |
| Secondary Subject Heading: | Diagnostics |
| Keywords: | Stroke medicine < INTERNAL MEDICINE, STROKE MEDICINE, Risk management < HEALTH SERVICES ADMINISTRATION & MANAGEMENT |
| | |

SCHOLARONE™ Manuscripts

*I, the Submitting Author has the right to grant and does grant on behalf of all authors of the Work (as defined in the below author licence), an exclusive licence and/or a non-exclusive licence for contributions from authors who are: i) UK Crown employees; ii) where BMJ has agreed a CC-BY licence shall apply, and/or iii) in accordance with the terms applicable for US Federal Government officers or employees acting as part of their official duties; on a worldwide, perpetual, irrevocable, royalty-free basis to BMJ Publishing Group Ltd ("BMJ") its licensees and where the relevant Journal is co-owned by BMJ to the co-owners of the Journal, to publish the Work in this journal and any other BMJ products and to exploit all rights, as set out in our licence.*

*The Submitting Author accepts and understands that any supply made under these terms is made by BMJ to the Submitting Author unless you are acting as an employee on behalf of your employer or a postgraduate student of an affiliated institution which is paying any applicable article publishing charge ("APC") for Open Access articles. Where the Submitting Author wishes to make the Work available on an Open Access basis (and intends to pay the relevant APC), the terms of reuse of such Open Access shall be governed by a Creative Commons licence – details of these licences and which Creative Commons licence will apply to this Work are set out in our licence referred to above.*

*Other than as permitted in any relevant BMJ Author's Self Archiving Policies, I confirm this Work has not been accepted for publication elsewhere, is not being considered for publication elsewhere and does not duplicate material already published. I confirm all authors consent to publication of this Work and authorise the granting of this licence.*

For peer review only

# The evaluation of microalbuminuria as a prognostic indicator after a TIA or minor stroke in an outpatient setting: The Prognostic Role of Microalbuminuria in TIA Evolution (ProMOTE) Study

Running Title: Predictive role of microalbuminuria after TIA and minor strokes

**W David Strain[1,2,4],**

**Salim Elyas[1,2],**

**Nicola Wedge[2,4],**

**Luke Mounce[3],**

**William Henley[3],**

**Martin A James[2],**

**Angela C Shore[1,4]**

1 Diabetes and Vascular Research Centre, University of Exeter Medical School. Barrack Road, Exeter EX2 5AX

2 Academic Department of Healthcare for Older People, Royal Devon & Exeter Hospital Barrack Road, Exeter EX2 5DW

3 University of Exeter, College of Medicine and Health, Exeter, UK

4 NIHR Exeter Clinical Research Facility

**Address for Correspondence:**

Dr David Strain

Diabetes and Vascular Research Centre

University of Exeter Medical School, Royal Devon & Exeter Hospital

Barrack Road, Exeter. EX2 5AX UK

**Email. d.strain@exeter.ac.uk**

**Tel: +441392 403058**

**Fax: +441392 403027**

For peer review only

| | | |
|---|---|---|
| **Word count** | Manuscript | 3168 |
| | Abstract | 334 |

**Disclosures**

All authors confirm that there are no conflicts of interest

For peer review only



For peer review only - http://bmjopen.bmj.com/site/about/guidelines.xhtml

**Abstract**:

**Objective**: Transient ischaemic attacks (TIA) and minor strokes are important risk factors for further vascular events. We explored the role of albumin-creatinine ratio (ACR) in improving risk prediction after a first event.

**Setting**: Rapid access stroke clinics in the UK

**Participants**. 2,202 patients attending with TIA or minor stroke diagnosed by the attending stroke physician, able to provide a urine sample to evaluate albumin creatinine ratio (ACR) using a near patient testing device.

**Primary and secondary outcomes**. Primary outcome was Major Adverse Cardiac Events (MACE; recurrent stroke, myocardial infarction, or cardiovascular death) at 90 days. The key secondary outcome was to determine whether urinary ACR could contribute to a risk prediction tool for use in a clinic setting.

**Results**: 151 MACE occurred in 144 participants within 90 days. Participants with a MACE had higher ACR than those without. A composite score awarding a point each for Age >80, Previous stroke/TIA and presence of microalbuminuria identified those at low and high risk. 90% of patients were at low risk (scoring 0 or 1). Their 90-day risk of a MACE was 5.7%. Of the remaining "high risk" population (scoring 2 or 3) 12.4% experienced MACE over 90 days (p<0.001 compared to low risk population). The need for acute admission in the first 7 days was two-fold elevated in the high-risk group compared to the low risk (3.23 vs. 1.43%; p=0.05). These findings were validated in an independent historic sample.

**Conclusion**: A risk score comprising age, previous stroke/TIA and microalbuminuria predicts future MACE events whilst identifying those at low risk of a recurrent event. This tool shows promise in the risk stratification of patients to avoid the admission of

For peer review only

low risk patients whilst assigning intensive interventions such as dual anti-platelet therapy to those at highest risk.

**Keywords**:

Stroke,

TIA,

Microalbuminuria,

ACR,

Risk prediction.

For peer review only

## Article Summary

*Strengths and limitations of this study*

- The pragmatic design of this study, provides good generalisability to clinical practice
- The predictive role of the combination of urinary ACR in combination with very basic demographics would be able to reassure the 90% of the population that there was only approximately a 1 in 20 risk of an event recurring, whereas the higher risk population had a 1 in 8 chance of a MACE outcome
- The study is limited by the availability of data to that which was available to the physicians at the time of the stroke appointment. This limits the ability to determine mechanistic association between ACR and MACE outcome
- Further work is required to determine whether therapies that reduce ACR can modify the risk of subsequent MACE

For peer review only

## Introduction:

Stroke is the second most common cause of death and a leading cause of disability worldwide[1]. Despite, or possibly because of, recent trends in reducing stroke mortality, the health and social disability burden of stroke is increasing[2]. After advancing age, transient ischaemic attacks (TIA) and minor strokes are the most important risk factors for recurrent stroke and predict long term mortality.[3,4] 23% of patients presenting with stroke have a history of TIA in the 3 months prior to the index event.[5] This is a key population to target for secondary prevention, but these patients represent less than 10% of all those who present to rapid access TIA clinics.[6,7] Notably, half of all completed strokes occur within the first week after TIA or minor stroke.[5]

Accurate identification of those patients presenting to TIA clinics with TIA or minor stroke who are most at risk for future events is important in order to i) intensify treatments such as giving dual anti-platelet therapy[8,9] ii) guide the need for urgent admission to facilitate the detection and urgent surgical correction of severe internal carotid artery (ICA) stenosis[10] iii) reassure patients with lowest risk.

Several risk stratification tools are already utilised in such clinics such as ABCD2[11,12] (awarding points for Age, presenting Blood pressure, Clinical features of unilateral weakness or aphasia, Duration of symptoms and Diabetes), California score[6] and imaging based scoring systems.[10,13] These tools lack optimal sensitivity and specificity.[12,14] Indeed some studies suggest patients with a 'low risk' ABCD2 score (less than 4/7) have similar 90 day stroke risk as patients deemed high risk with an ABCD2 score above 4/7,[15] whilst missing up to 40% of patients with severe ICA stenosis.[16] As such, recent consensus guidelines have advised against their use.[17-19]


For peer review only - http://bmjopen.bmj.com/site/about/guidelines.xhtml

Increased urinary albumin excretion rate (AER) has been shown to predict incident stroke and heart failure in people with diabetes. In the general population, AER predicts cardiovascular disease and mortality post-stroke independent of conventional cardiovascular risk factors such as hypertension, diabetes and smoking [20-23]. Urinary albumin creatinine ratio (ACR) is a well recognised proxy for urinary AER, and can now be assessed using simple and inexpensive point of care equipment. In an earlier pilot study of 142 patients with minor stroke/TIA,[24] we identified the potential role of urinary ACR in identifying those at highest risk of a recurrent event. Although statistically significant, this study was not large enough to explore potential confounding. We therefore performed a definitive study to assess whether urinary ACR improved the risk stratification of patients presenting with TIA or minor stroke to UK acute stroke clinics.

**Methods:**

Patients diagnosed with suspected minor stroke/TIA by a consultant stroke physician, attending a rapid access clinics in one of 12 UK hospitals were recruited at the end of their clinic consultation. As these clinics are rapid access, often within the first 24 hours of symptoms, it was impossible to accurately differentiate between TIA (with symptoms less than 24 hours) and minor stroke in all cases. Only individuals with events that had occurred within the previous month were included in the study. Routine clinical care, including urgent revascularisation in those with severe ICA stenosis (> 50% diameter stenosis by the North American Symptomatic Carotid Endarterectomy Trial (NASCET) method,[25] was initiated prior to enrollment. If a cardioembolic source was identified, anticoagulants were initiated according to local protocols. After written,



For peer review only - http://bmjopen.bmj.com/site/about/guidelines.xhtml

informed consent was obtained in the clinic, demographics, including age, sex, height, weight, past medical history and ABCD2 score were recorded. Times from onset of symptoms to assessment and enrollment in the study were documented. A clean specimen of urine was collected from participants in the clinic and tested with a Unistix dipstick and, if there was no indication of urinary tract infection (presence of any two of leucocytes, nitrites and/or protein), ACR was measured using a point of care analyzer (The Afinion™ AS100 Analyzer, Axis Shield, Dundee, UK). This system uses an immunometric membrane flow-through principle for albumin measurement and an enzymatic colorimetric test for creatinine quantification[26] and reports an ACR in approximately 5 minutes within the range 0.1 to 140 mg/mmol with a coefficient of variation of 4.6-6%.[26] Participants with values of less than 0.1 mg/mmol were recorded as having the lowest recordable value of ACR (0.1 mg/mmol).

Participants were followed up by telephone on day 7, 30 and 90, and any history of any further vascular events, hospitalisation or death was obtained. If contact with participants was not possible initially, the presence or absence of further events was verified during additional attempts at contact or during future follow up. If no further contact was possible with the participant (e.g. in the case of significant stroke or death), medical history was collected from the next of kin and verified from the primary care physician and hospital records. All reported clinical events were adjudicated according to standardised diagnostic criteria by a data monitoring committee including two independent stroke physicians, blinded to the ACR, by examination of clinical records if they attended hospital, or their primary care physician, or by verifying their clinical symptoms from the research records if patients did not seek further medical advice. In the event of disagreement, a third independent stroke physician acted as adjudicator.



For peer review only - http://bmjopen.bmj.com/site/about/guidelines.xhtml

The primary outcome was to determine the utility of microalbuminuria in predicting the time to first major adverse cardiovascular event (MACE; recurrent stroke, myocardial infarction or death) within 90 days. Secondary outcomes were time to explore the predictive role of microalbuminuria on recurrent stroke, total mortality (even if these were not the first MACE event), the presence of first MACE within 7 days, and the need for hospitalisation within the first 7 days. All events were adjudicated blind to urinary albumin creatinine ratio by an independent data monitoring committee.

*Ethics Statement*

The study protocol was approved by the national research ethics committee (approval 14/EE/1106). All participants provided written informed consent prior to enrolment and confirmed their willingness to continue participation at each telephone consultation.

*Patient and public involvement*

Patients were involved in the design and conduct of this research. During the feasibility stage, priority of the research question, and methods of recruitment were informed by discussions with patients through a focus group session and three structured interviews. During the trial, two patients joined the independent trial steering committee. Once the trial has been published, participants will be informed of the results through a dedicated newsletter suitable for a non-specialist audience, led by one of the patients on the steering committee.

*Statistical analysis:*



For peer review only - http://bmjopen.bmj.com/site/about/guidelines.xhtml

Data were treated as continuous variables wherever possible to maximize power. All normally distributed data are presented as mean ± standard deviation (SD). Skewed data were appropriately transformed and presented as geometric means (with 95% confidence intervals [CI]). Statistical significance for categorical variables was calculated using the chi-squared test and the Student's t-test for continuous variables. Independence of ACR (as a risk predictor) from diabetes and other measures of the ABCD2 score was assessed using logistic regression. Microalbuminuria was defined using the currently accepted clinical thresholds for microalbuminuria used in people with diabetes 3.5mg/mol for women and 2.5mg/mol for men. Identification of independent predictors of recurrent MACE events was performed by backwards stepwise logistic regression, commencing with all information available for the complete dataset, representing all information that would routinely be available to the clinician in the clinic setting. Where multiple related measures were available (e.g. systolic, diastolic and mean arterial blood pressure) the variable that accounted for the greatest degree of variance in a univariate analysis was included. Fractional polynomial regression modeling was used to identify sex-specific thresholds of ACR to define "microalbuminuria" in the context of stroke risk stratification. Prediction of events were calculated using a Cox proportional hazard ratio with time to first MACE as the primary outcome. In keeping with the recommendations of Cupples[27] and Rothman[28] the measured significance of the variables of interest is reported without adjustment for multiple testing. Statistical significance was considered at $p<0.05$. Statistical analysis was performed using Stata SE 14.2 (Mac version: Statacorp ltd Texas).

**Results:**


For peer review only - http://bmjopen.bmj.com/site/about/guidelines.xhtml

Two thousand, four hundred and eight patients with a diagnosis of definite or probable minor stroke/TIA were recruited; 149 participants were subsequently excluded after the diagnosis was revised to that of a stroke mimic, 8 withdrew consent and a further 49 (2%) were excluded due to intercurrent urinary tract infection rendering the assessment of ACR invalid. No patient was lost to follow-up. Baseline characteristics are presented in table 1. Of the 2,202 included in the final analysis, most were male (64.9%). All patients were commenced on secondary prevention with appropriate antiplatelet or anticoagulant therapy, and an appropriately dosed statin in accordance with contemporaneous national guidelines.

Over 90 days, 151 primary outcome events (MACE) occurred in 144 participants (6.7% of patients) including 8 cardiovascular deaths. There were also 8 non-cardiovascular deaths. Within 7 days there were 38 MACE in 36 participants. Those with MACE were more likely to have had previous stroke/TIA, however, with this exception, there were no significant differences in routinely collected clinic data (Table 1). There was no difference in mean ABCD2 score or the proportion of participants with a 'high risk' score (4/7 or more) between those who did and did not have events by 90 days. When looking exclusively at the 1374 participants identified with a "high risk" ABCD2 score (63% of the study sample; 93% male), a high score did not distinguish those at high risk of MACE event compared to those with an ABCD2 <4 (HR 1.15 (0.82-1.63); p=0.4), nor did a low risk score exclude an event, with only 37.7% of people who did not have experience a MACE outcome scoring below 4. Point-of-care ACR was higher in those participants with a primary outcome event at 90 days compared to those without (2.27 (1.92-2.68) vs. 1.75 (1.68–1.83) mg/mmol respectively; p=0.002). This



For peer review only - http://bmjopen.bmj.com/site/about/guidelines.xhtml

distinction remained after adjustment for age and sex (adjusted ACR 2.40 (2.04-2.83) vs. 1.88 (1.80 – 1.96), p=0.004. Figure 1).

*Evaluation of the prognostic role of microalbuminuria alone*

Fractional polynomial regression modelling identified the sex-specific threshold of risk for ACR aligned with the currently accepted thresholds for microalbuminuria used in people with diabetes of 2.5mg/mmol for men and 3.5mg/mol for women. These thresholds identified 562 participants (25.4% of the study sample; 75% male) with microalbuminuria. These participants were more likely than those with a low ACR to experience a primary outcome event (Hazard Ratio 1.66 (95% CI 1.19 - 2.34); p=0.003) and had a greater than 5-fold increase in 90-day mortality (HR 5.44 (1.86 – 15.90); p=0.0003). Alone, however, microalbuminuria did not have sufficient positive or negative predictive value to justify its clinical utility (Positive predictive value – 9.1%, Specificity 75.4%).

*Generating a composite risk score for 90-day risk stratification*

In a stepwise multivariate model, the only independent predictors of MACE were elevated ACR, age greater than 80 and history of previous stroke or TIA. Similar variance within the model was explained microalbuminuria (i.e. an ACR >2.5 and 3.5mg/mol respectively in men and women) as being older than 80 years and previous history of stroke/TIA, so they were afforded equal weighting. This generated a 4-point scale between 0 and 3 (termed the ‘APA’ score representing Age, Prior stroke or TIA and elevated ACR), with the lowest risk participants having a score of 0 (Table 2). There was a sequential increase in risk with increasing score such that those with a score of 0 having a 90-day risk of 4.86% rising to a 30% risk for those with the highest


For peer review only - http://bmjopen.bmj.com/site/about/guidelines.xhtml

score (supplementary table 1). The population was then divided into a low risk group scoring 0 or 1 or high risk scoring 2 or 3. 1,985 (90.1%) of participants were identified as low risk compared to 217 (9.9%) in the high-risk group. When comparing the positive predictive value (PPV) sensitivity and specificity of the APA score compared to the previously available parameters of age and previous stroke demonstrated a clinically meaningful improvement over each measure on its own (Supplementary table 2a). Compared to using previous stroke alone, APA score had a similar positive predictive value (12.4% for APA score vs 11.9% for previous stroke) and specificity (91.2% vs 96.4%, respectively), however superior sensitivity (19.3% vs 7.1%, respectively). Sensitivity was improved by using a composite of previous stroke and being over the age of 80 years, however this was at the expense of a reduction of positive predictive value to 8.4%.

The high-risk group was older, with higher systolic blood pressure and pulse pressure compared to the low-risk group (table 3). The 90-day risk of a MACE outcome was 5.7% in the low-risk group, versus 12.4% in the high-risk group ($p<0.001$), translating to a hazard ratio of 2.12 (95% CI 1.38 – 3.25) for the high-risk score compared to low-risk group ($p<0.001$; Figure 2). This was predominantly driven by an increase in recurrent stroke/TIA (3.3% vs. 8.8%; HR 2.67(1.58-4.51); $p<0.001$). This difference was apparent within 7 days of the clinic, such that those with a high APA score had a 2.2-fold increased risk of a recurrent event.

*Validation of APA score on a historic sample*

In order to validate the APA score, we applied the tool to the individuals who participated in the pilot study but did not contribute their data to this dataset. Details of



For peer review only - http://bmjopen.bmj.com/site/about/guidelines.xhtml

this pilot study have been published elsewhere [24]. In brief, the pilot study recruited 139 participants over 9 months from one of the centres involved in the definitive study. In this population, there were 13 recurrent events (9.35%) within the 90 days, 9 of which occurred in the first 7 days after attendance to the stroke clinic (Table 4). The APA risk score replicated a similar distribution of number of participants at low risk (73%) and high risk (27%) as in the larger study. Compared to the component parts, the APA score had superior positive predictive value (24.4% vs 18.2% for Previous Stroke or 13.3% for previous stroke and being aged >80 years) and superior sensitivity (76.9% vs 46.2% vs 15.4% respectively: supplementary table 2b). At 90 days, 5.9% of the low APA group had recurrent events compared to 18.4% of the in the high-APA group (p=0.02). Again, within the first 7 days this was apparent with only 1.9% of the low APA group experiencing the primary outcome, compared to 13.2% in the high APA group (p=0.04).

**Discussion:**

In this large UK multicentre prospective study with blinded outcome adjudication, we have demonstrated the utility of an elevated ACR, measured using a simple point-of-care analyser, in combination with simple clinical data (age and previous history of stroke or TIA) in the prediction of major adverse cardiovascular events and death. This differentiates between low and high-risk individuals. Specifically, over 90% of individuals were identified as low risk with less than a 1.5% risk of a major adverse cardiovascular event or death over the next 7 days, increasing to 5.7% by 90 days, whereas 9.9 % of the population were identified at high risk with a 12.4% event rate over 90 days. Although only a small number, our findings were validated in a separate historic population who participated in the pilot study.


For peer review only - http://bmjopen.bmj.com/site/about/guidelines.xhtml

The utility of APA scoring may go beyond simply guiding conversations regarding prognosis in a clinic setting. The POINT[8] and CHANCE[9] studies demonstrated the benefit of dual anti-platelet therapy in the first 21 days after high risk TIA. There was, however, a 0.5% absolute increase in major intracranial haemorrhage. We would propose that an assessment of the APA score would be appropriate prior to initiating therapy. In those with a low risk APA score, even the 25% relative risk reduction in the POINT trial would result in only a marginally beneficial risk:benefit ratio. Those with a high APA score, however, would likely benefit from dual-antiplatelet therapy with a number need to treat of less than 30 in order to prevent an event. This suggestion, however, does require confirmation in an independent study. The APA score also gives additional information that is of use in the more acute setting. Over the first 7 days, a low-risk APA score had a negative predictive value of >98.5% for the risk of a MACE - a number very similar to the predictive effect of troponin when evaluating suspected cardiac chest pain.[29]

When considering the populations identified by the APA score, there are similarities to the ABCD2 score. Indeed, the demographics of the high-risk group were older, with higher blood pressure and a trend towards more diabetes. We would suggest, that microalbuminuria represents vascular susceptibility to the adverse consequences of hypertension and diabetes rather than simply an indication of the presence of hypertension and /or diabetes in general. This would explain why the APA score predicted further MACE and total mortality at 7 and 90 days, whereas in our large sample the ABCD2 score did not.

For peer review only

Elevated ACR is a recognised marker of generalised endothelial and microvascular dysfunction. The increased filtration of albumin through the renal glomerular filtration barrier is thought to be due to changes in the chemical and physical properties of this endothelial barrier and its glycocalyx.[30-32] The mechanism explaining the association between microalbuminuria and incident cardiovascular events is thought to depend on its role as a marker of systemically increased vascular permeability and altered homeostasis, coagulation and endothelial function.[33-38] However, after acute ischaemic events such as myocardial infarction, or as demonstrated here TIA or minor stroke, it is likely that elevated urinary ACR is, at least in part, dependent on the systemic inflammatory response to the original insult.

Whilst unlikely to be mechanistically involved in the progression of disease, the question as to whether it is a good surrogate of therapeutic effect remains. Improving systemic inflammation, such as through the pleiotropic effect of statins, has been associated with simultaneous improvements in urinary albumin excretion and CV event rates in patients with elevated high sensitivity C-reactive protein (hsCRP).[39-41] Further studies are needed to determine whether microalbuminuria after acute stroke represents a therapeutic target amenable to treatment, and if reductions in microalbuminuria are associated with reductions in the vascular events they predict.

*Limitations and strengths of study*

Recurrent event rate was lower than anticipated from our pilot study and contemporaneous clinical trials such as CHANCE (11.7% in the control arm),[9] although are comparable with the recently-reported randomised controlled POINT trial (6%).[8] In usual practice it is usually 24-48 hours between the index event and clinic attendance.



For peer review only - http://bmjopen.bmj.com/site/about/guidelines.xhtml

Given that data from previous studies has demonstrated the highest risk occurs in the first 48 hours, it is likely that some events will have occurred between the index event and attendance in the clinic in our study.[42] The pragmatic design of the study, however, is also a strength given that the predictive role highlights those that may benefit from dual anti-platelet therapy or hospitalisation when the patient is seen in the acute stroke clinic.

There is no consensus on the gold standard for diagnosing TIA in a rapid access stroke clinic.[43] In order to maintain the generalisability of the study, all attendees of the stroke clinic that were diagnosed with a probable or definite TIA were invited to participate. Only a small number of patients were subsequently withdrawn as being stroke mimics. Outcome events were rigorously adjudicated against published diagnostic criteria to ensure consistency and reliability.

Finally, the use of a single urinary ACR sample obtained during the clinic assessment using a near patient testing kit is not as robust as a sample tested in a central laboratory. Whilst there is significant diurnal variability in urinary AER within individuals, potentially limiting the scientific or mechanistic merit of the study compared to studies using overnight or 24-hour urine collection, the pragmatic design makes this study more applicable to general clinic populations. Further, the relatively low cost of the near patient test kit makes it accessible in most emergency settings, in primary and secondary care.

**Conclusion:**



For peer review only - http://bmjopen.bmj.com/site/about/guidelines.xhtml

We have demonstrated for the first time the potential utility of a single point of care test of urinary ACR in patients presenting with TIA and minor stroke as a prediction tool to assist triaging patients. When used in combination with patient age and previous history of stroke or TIA, this generated a risk stratification score (the APA score: Age>80, Past history of stroke/TIA, and elevated ACR) that could reliably identify the large proportion of patients with a low risk of recurrent vascular event over the next 90 days. The value of ACR that is most predictive is aligned with the conventional definition of microalbuminuria used in diabetes, suggesting that in clinical practice the existing technology for evaluating microalbuminuria including urine dipsticks may be used. A high APA score was associated with a doubling of risk of MACE outcome and a five fold increase in mortality. This APA score was validated in an independent population. We believe the APA score may represent a practical tool for clinicians engaged in the acute ambulatory assessment of TIA and minor stroke, to assist in the assessment of risk:benefit ratio for the use dual anti-platelet therapy or to admit for more urgent investigation. Further work is required to determine whether the increase in ACR represents a therapeutic target or solely a prognostic indicator.

For peer review only


For peer review only - http://bmjopen.bmj.com/site/about/guidelines.xhtml

**Contributorship statement**

WDS & ACS conceived and designed the study, attained funding, were involved in the management of the trial. WDS produced the first draft of the manuscript. SE, NW & MAJ contributed to the design and management of the trial. LM &WH provided the statistical support for the management of the trial and the analysis of the outcomes. All authors have reviewed the final manuscript prior to submission.

**Competing interests**

No authors report any competing interests relevant to this manuscript.

**Funding**

This study was funded by a NIHR Research for Patient Benefit grant (PB-PG-1112-29069) and supported by the NIHR Exeter Clinical Research Facility.

**Data sharing statement**

Anonymised data is available from the corresponding author on reasonable request.

**Acknowledgements**

WDS is supported by the NIHR Exeter Clinical Research Facility. WDS and MAJ is supported by the NIHR SW Peninsula Applied Research Collaboration. This manuscript does not necessarily reflect the views of the NIHR, the Exeter Clinical Research Facility, the NHS or the UK Department of Health.

For peer review only

**References:**


1. Feigin VL, Norrving B, Mensah GA. Global Burden of Stroke. *Circ Res.* 2017;120(3):439-448.
2. Henssge U. National Sentinel Stroke Clinical Audit 2010. In: Royal College of Physicians, ed2011.
3. Burn J, Dennis M, Bamford J, Sandercock P, Wade D, Warlow C. Long-term risk of recurrent stroke after a first-ever stroke. The Oxfordshire Community Stroke Project. *Stroke; a journal of cerebral circulation.* 1994;25(2):333-337.
4. Hankey GJ. Long-term outcome after ischaemic stroke/transient ischaemic attack. *Cerebrovascular diseases (Basel, Switzerland).* 2003;16 Suppl 1:14-19.
5. Rothwell PM, Warlow CP. Timing of TIAs preceding stroke: time window for prevention is very short. *Neurology.* 2005;64(5):817-820.
6. Johnston SC, Gress DR, Browner WS, Sidney S. Short-term prognosis after emergency department diagnosis of TIA. *Jama.* 2000;284(22):2901-2906.
7. Hill MD, Yiannakoulias N, Jeerakathil T, Tu JV, Svenson LW, Schopflocher DP. The high risk of stroke immediately after transient ischemic attack: a population-based study. *Neurology.* 2004;62(11):2015-2020.
8. Johnston SC, Easton JD, Farrant M, et al. Clopidogrel and Aspirin in Acute Ischemic Stroke and High-Risk TIA. *N Engl J Med.* 2018;379(3):215-225.
9. Wang Y, Wang Y, Zhao X, et al. Clopidogrel with aspirin in acute minor stroke or transient ischemic attack. *N Engl J Med.* 2013;369(1):11-19.
10. Kiyohara T, Kamouchi M, Kumai Y, et al. ABCD3 and ABCD3-I scores are superior to ABCD2 score in the prediction of short- and long-term risks of stroke after transient ischemic attack. *Stroke; a journal of cerebral circulation.* 2014;45(2):418-425.


For peer review only

11. Rothwell PM, Giles MF, Flossmann E, et al. A simple score (ABCD) to identify individuals at high early risk of stroke after transient ischaemic attack. *Lancet.* 2005;366(9479):29-36.

12. Bhatt A, Jani V. The ABCD and ABCD2 Scores and the Risk of Stroke following a TIA: A Narrative Review. *ISRN Neurol.* 2011;2011:518621.

13. Ay H, Arsava EM, Johnston SC, et al. Clinical- and imaging-based prediction of stroke risk after transient ischemic attack: the CIP model. *Stroke; a journal of cerebral circulation.* 2009;40(1):181-186.

14. Giles MF, Rothwell PM. Systematic review and pooled analysis of published and unpublished validations of the ABCD and ABCD2 transient ischemic attack risk scores. *Stroke; a journal of cerebral circulation.* 2010;41(4):667-673.

15. Amarenco P, Labreuche J, Lavallée PC. Patients With Transient Ischemic Attack With ABCD2 <4 Can Have Similar 90-Day Stroke Risk as Patients With Transient Ischemic Attack With ABCD2 ≥4. *Stroke; a journal of cerebral circulation.* 2012;43(3):863-865.

16. Amarenco P, Labreuche J, Lavallee PC. Patients with transient ischemic attack with ABCD2 <4 can have similar 90-day stroke risk as patients with transient ischemic attack with ABCD2 >/=4. *Stroke; a journal of cerebral circulation.* 2012;43(3):863-865.

17. National Institute of Health and Care Excellence. Stroke and transient ischaemic attack in over 16s: diagnosis and initial management; NICE guideline [NG128]. National Institute of Health and Care Excellence. Published 2019. Updated May 2019. Accessed 10/11/20, 2019.

18. Powers WJ, Rabinstein AA, Ackerson T, et al. 2018 Guidelines for the Early Management of Patients With Acute Ischemic Stroke: A Guideline for



For peer review only - http://bmjopen.bmj.com/site/about/guidelines.xhtml

Healthcare Professionals From the American Heart Association/American Stroke Association. *Stroke.* 2018;49(3):e46-e110.

19. Ahmed N, Audebert H, Turc G, et al. Consensus statements and recommendations from the ESO-Karolinska Stroke Update Conference, Stockholm 11–13 November 2018. *European Stroke Journal.*0(0):2396987319863606.

20. Gerstein HC, Mann JF, Yi Q, et al. Albuminuria and risk of cardiovascular events, death, and heart failure in diabetic and nondiabetic individuals. *JAMA.* 2001;286(4):421-426.

21. Hillege HL, Fidler V, Diercks GF, et al. Urinary albumin excretion predicts cardiovascular and noncardiovascular mortality in general population. *Circulation.* 2002;106(14):1777-1782.

22. Yuyun MF, Khaw KT, Luben R, et al. Microalbuminuria, cardiovascular risk factors and cardiovascular morbidity in a British population: the EPIC-Norfolk population-based study. *Eur J Cardiovasc Prev Rehabil.* 2004;11(3):207-213.

23. Strain WD, Shore AC, Melzer D. Albumin:creatinine ratio predicts mortality after stroke: analysis of the Third National Health and Nutrition Examination Survey. *J Am Geriatr Soc.* 2010;58(12):2434-2435.

24. Elyas S, Shore AC, Kingwell H, et al. Microalbuminuria could improve risk stratification in patients with TIA and minor stroke. *Ann Clin Transl Neurol.* 2016;3(9):678-683.

25. Morgenstern LB, Fox AJ, Sharpe BL, Eliasziw M, Barnett HJ, Grotta JC. The risks and benefits of carotid endarterectomy in patients with near occlusion of the carotid artery. North American Symptomatic Carotid Endarterectomy Trial (NASCET) Group. *Neurology.* 1997;48(4):911-915.



For peer review only - http://bmjopen.bmj.com/site/about/guidelines.xhtml

26. Kvam C. Afinion ACR Scientific Poster AACC 2007. Axis-Shield PoC AS. http://www.afinion.net/publications. Published 2007. Accessed.

27. Cupples LA, Heeren T, Schatzkin A, Colton T. Multiple testing of hypotheses in comparing two groups. *Ann Intern Med.* 1984;100(1):122-129.

28. Rothman KJ. No adjustments are needed for multiple comparisons. *Epidemiology.* 1990;1(1):43-46.

29. Gualandro DM, Puelacher C, LuratiBuse G, et al. Comparison of high-sensitivity cardiac troponin I and T for the prediction of cardiac complications after non-cardiac surgery. *Am Heart J.* 2018;203:67-73.

30. Haraldsson B, Jeansson M. Glomerular filtration barrier. *Curr Opin Nephrol Hypertens.* 2009;18(4):331-335.

31. Singh A, Satchell SC. Microalbuminuria: causes and implications. *Pediatr Nephrol.* 2011;26(11):1957-1965.

32. Gansevoort RT, Lambers H, Witte EC. Methodology of screening for albuminuria. *Nephrology Dialysis Transplantation.* 2007;22(8):2109-2111.

33. Barzilay JI, Peterson D, Cushman M, et al. The relationship of cardiovascular risk factors to microalbuminuria in older adults with or without diabetes mellitus or hypertension: the cardiovascular health study. *Am J Kidney Dis.* 2004;44(1):25-34.

34. Valmadrid CT, Klein R, Moss SE, Klein BE. The risk of cardiovascular disease mortality associated with microalbuminuria and gross proteinuria in persons with older-onset diabetes mellitus. *Arch Intern Med.* 2000;160(8):1093-1100.

35. Dinneen SF, Gerstein HC. The association of microalbuminuria and mortality in non-insulin-dependent diabetes mellitus. A systematic overview of the literature. *Arch Intern Med.* 1997;157(13):1413-1418.



For peer review only - http://bmjopen.bmj.com/site/about/guidelines.xhtml

36. Festa A, D'Agostino R, Howard G, Mykkanen L, Tracy RP, Haffner SM. Inflammation and microalbuminuria in nondiabetic and type 2 diabetic subjects: The Insulin Resistance Atherosclerosis Study. *Kidney Int.* 2000;58(4):1703-1710.

37. Asselbergs FW, de Boer RA, Diercks GF, et al. Vascular endothelial growth factor: the link between cardiovascular risk factors and microalbuminuria? *Int J Cardiol.* 2004;93(2-3):211-215.

38. Stehouwer CD, Gall MA, Twisk JW, Knudsen E, Emeis JJ, Parving HH. Increased urinary albumin excretion, endothelial dysfunction, and chronic low-grade inflammation in type 2 diabetes: progressive, interrelated, and independently associated with risk of death. *Diabetes.* 2002;51(4):1157-1165.

39. Ridker PM, Danielson E, Fonseca FA, et al. Rosuvastatin to prevent vascular events in men and women with elevated C-reactive protein. *N Engl J Med.* 2008;359(21):2195-2207.

40. Abe M, Maruyama N, Okada K, Matsumoto S, Matsumoto K, Soma M. Effects of lipid-lowering therapy with rosuvastatin on kidney function and oxidative stress in patients with diabetic nephropathy. *J Atheroscler Thromb.* 2011;18(11):1018-1028.

41. Girardi JM, Farias RE, Ferreira AP, Raposo NR. Rosuvastatin prevents proteinuria and renal inflammation in nitric oxide-deficient rats. *Clinics (Sao Paulo).* 2011;66(8):1457-1462.

42. Coull AJ, Rothwell PM. Underestimation of the early risk of recurrent stroke: evidence of the need for a standard definition. *Stroke; a journal of cerebral circulation.* 2004;35(8):1925-1929.



For peer review only - http://bmjopen.bmj.com/site/about/guidelines.xhtml

43. Graham C, Bailey D, Hart S, et al. Clinical diagnosis of TIA or minor stroke and prognosis in patients with neurological symptoms: A rapid access clinic cohort. *PLoS One.* 2019;14(3):e0210452.

For peer review only

**Table Legends (ProMOTE Study):**

**Table 1**: Sample characteristics of total population and population stratified by occurrence of recurrent major adverse cardiovascular events (MACE) at 90 days

Figures presented as mean (SD) for continuous variable except ACR where geometric mean (Inter-quartile range) presented; % for categorical data

P for continuous data by Student's t-test

P for categorical data by Chi Squared test

BMI = Body Mass Index,

ABCD2 (A=Age, B=BP, C=Clinical features, D= Duration of symptoms + Diabetes),

ACR Albumin Excretion Rate

*Note*: Total Cholesterol was collected from routine clinical practice records therefore is not available for all participants

**Table 2**: APA (Age, Previous Stroke and ACR) Score derivation

**Table 3**: Occurrence of events by APA risk score group (mean ± SD for continuous variable, p for ANOVA; % for categorical data, p for Chi-Squared)

**Table 4:** Occurrence of events by APA risk score group in independent validation population (mean ± SD for continuous variable, p for ANOVA; % for categorical data, p for Chi-Squared)

For peer review only

Table 1: Sample characteristics of total population and population stratified by occurrence of recurrent vascular events or death (MACE) at 90 days.

| Characteristic | Total population | No event by 90 days | Event at 90 days |
|---|---|---|---|
| N | 2202 | 2058 | 144 |
| Age (years) | 70.9 (11.7) | 70.9 (11.6) | 72.2 (12.2) |
| Sex (%male) | 64.8 | 64.7 | 67.9 |
| Height (m) | 1.70 (0.10) | 1.70 (0.10) | 1.71 (0.9) |
| Weight (kg) | 79.8 (17.1) | 79.9 (17.0) | 79.7 (17.7) |
| BMI (kg/m$^2$) | 27.6 (5.2) | 27.6 (5.2) | 27.2 (4.9) |
| Blood pressure | | | |
| Systolic (mmHg) | 144.1 (20.8) | 144.2 (20.9) | 145.7 (21.7) |
| Diastolic (mmHg) | 81.8 (12.6) | 81.7 (12.6) | 83.8 (13.1) |
| Total Cholesterol§ | (n = 1,466) | (n=1,374) | (n=92) |
| (mmol/L) | 5.02 (2.57) 194 | 5.03 (2.63) | 4.75 (1.24) |
| (mg/dl) | 194(99) | 195 (102) | 183 (48) |
| Diabetes (%) | 15.7 | 15.9 | 12.1 |
| Previous Stroke (%) | 4.0 | 3.5 | 10.7* |
| ABCD2 | | | |
| Mean score out of 7 | 4.0 (1.4) | 4.0 (1.4) | 4.1 (1.4) |
| Proportion >4 (%) | 62.5 | 62.3 | 65.0 |
| "High Risk" | | | |
| ACR (mg/mmol)† | 1.79(0.9-2.9) | 1.75(1.69-1.83) | 2.27(1.92-2.68)** |
| ACR (mg/g)† | 15.8(8.0-25.6) | 15.5 (14.9-16.2) | 20.1 (17.0-23.7)** |

§ These were collected from patient records where clinician had requested the test and therefore only available in a subset of the population

† Geometric mean (95% CI)

*P<0.0001 compared to those with no events

** p=0.002 compared to those with no events



For peer review only - http://bmjopen.bmj.com/site/about/guidelines.xhtml

**Table 2: APA (Age, Previous Stroke and ACR) Score derivation**

| | Variable | Value |
|---|---|---|
| **Age** | ≥80 years | 1 |
| **Previous stroke or TIA** | Present | 1 |
| **ACR** | ≥3.5 mg/mmol for women<br>≥2.5 mg/mol for men | 1 |

| **Score** | 0-1 | 2-3 |
|---|---|---|
| **Risk** | Low Risk | High Risk |

ACR: Albumin Creatinine Ratio

For peer review only

Table 3: Occurrence of events by APA risk score group (mean ± SD for continuous variable, p for ANOVA; % for categorical data, p for Chi-Squared)

| | Low Risk | High Risk | p |
|---|---|---|---|
| n | 1,985 | 217 | |
| Sex (% Male) | 64.5 | 68.2 | 0.2 |
| Age (years) | 69.6 (11.3) | 84.0 (5.0) | <0.0001 |
| Blood pressure (mmHg) | | | |
| Systolic | 143 (20.4) | 150.5 (23.3) | <0.0001 |
| Diastolic | 82 (12.3) | 79 (11.6) | 0.0007 |
| Weight (kg) | 80.3 (17.1) | 75.1 (16.2) | <0.0001 |
| Diabetes | 15.3 | 18.9 | 0.2 |
| MACE at 90 days (%) | | | |
| All events | 5.7 | 12.4 | <0.0001 |
| Cerebrovascular event | 3.2 | 8.8 | <0.0001 |
| Cardiovascular Mortality | 0.1 | 1.4 | <0.001 |
| All Cause Mortality | 0.4 | 2.3 | 0.001 |
| MACE at 7 days (%) | 1.46 | 3.23 | 0.05 |

For peer review only



For peer review only - http://bmjopen.bmj.com/site/about/guidelines.xhtml

**Table 4**: Occurrence of events by APA risk score group in independent validation population (mean ± SD for continuous variable, p for ANOVA; % for categorical data, p for Chi-Squared)

| | Low Risk | High Risk | p |
|---|---|---|---|
| n | 101 | 38 | |
| Sex (% Male) | 51.5 | 71.5 | 0.04 |
| Age (years) | 72.6 (10.7) | 81.6 (6.1) | 0.001 |
| Blood pressure (mmHg) | | | |
| Systolic | 142.6 (20.2) | 147.3 (26.6) | 0.3 |
| Diastolic | 77.7 (9.8) | 77.4 (10.8) | 0.9 |
| Weight (kg) | 76.2 (16.4) | 75.0 (15.4) | 0.7 |
| | | | |
| Recurrent event at 90 days (%) | | | |
| MACE event | 5.9 | 18.4 | 0.02 |
| | | | |
| Recurrent event at 7 days (%) | | | |
| MACE events* | 1.9 | 13.2 | 0.04 |

* MACE = Major Adverse Cardiovascular Event – Recurrent stroke or TIA, Myocardial infarction or cardiovascular death.

For peer review only

**Figure 1:** Age and sex adjusted Albumin Creatinine Ratio (Mean +/-95% CI) stratified by the occurrence of a Major Adverse Cardiac Event (MACE) in the first 90 days post initial event

MACE – Major Adverse Cardiovascular event = recurrent cerebrovascular event, myocardial infarction or cardiovascular death

**Figure 2**: Time to first Major Adverse Cardiac Event (MACE) stratified by APA (Age, Previous cerebrovascular event, High ACR) risk score. Event rate in high risk (n=217) 12.44% vs. low risk (n=1985) 5.69%, HR 2.12 (1.38 – 3.25) p<0.001

For peer review only

For peer review only



For peer review only - http://bmjopen.bmj.com/site/about/guidelines.xhtml

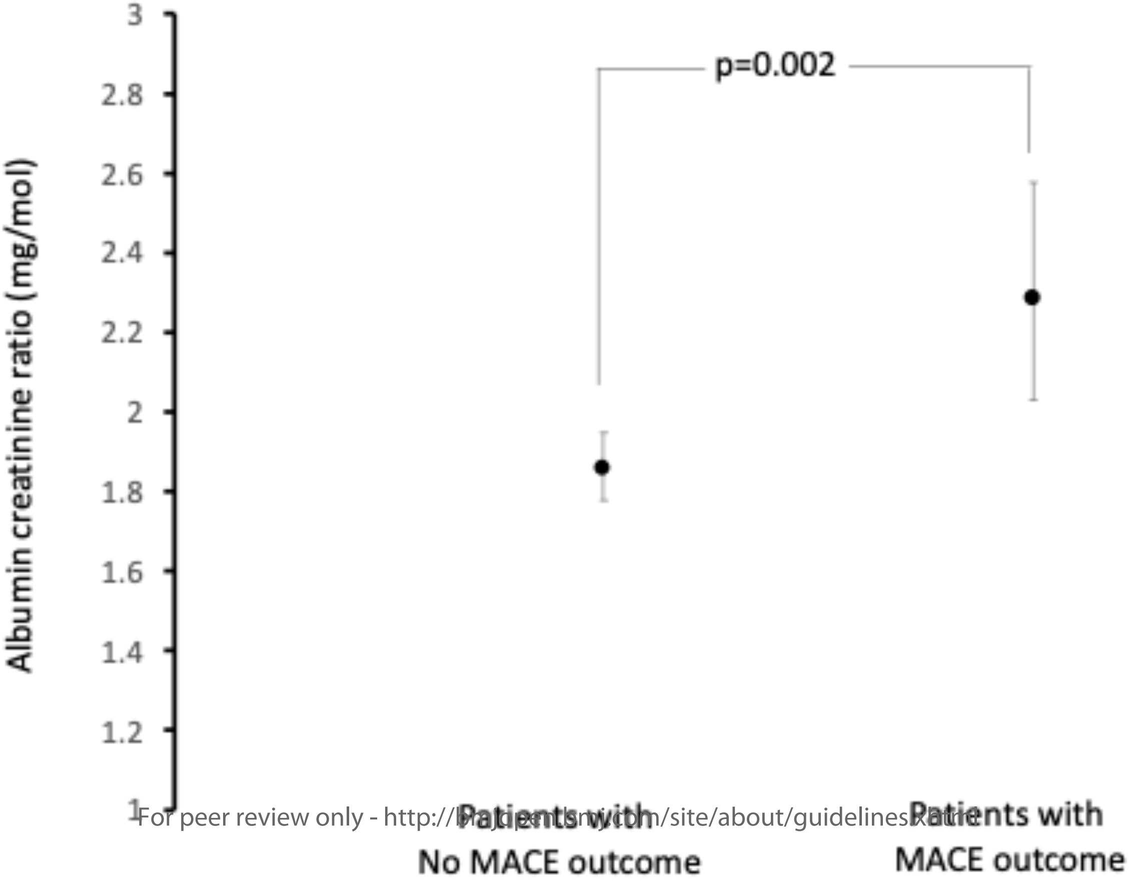


For peer review only - http://bmjopen.bmj.com/site/about/guidelines.xhtml

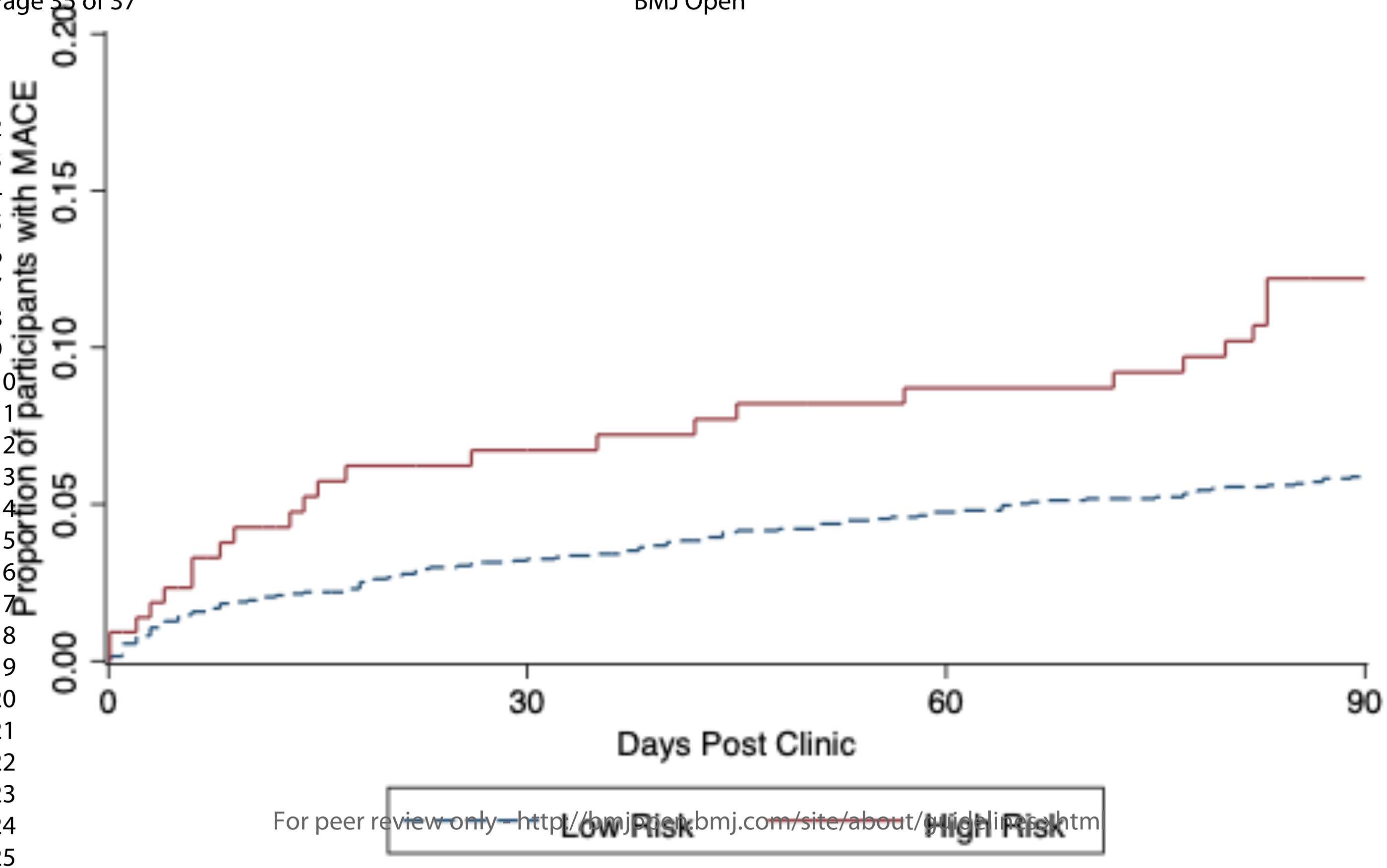
Proportion of participants with MACE
0.00
0.05
0.10
0.15
0.20
0
30
60
90
Days Post Clinic
Low Risk
High Risk

**Supplementary Table 1**: Occurrence of major adverse cardiac events (MACE) by APA risk score group (p for Chi-Squared)

| APA score | N | Occurrence of MACE events (%) |
|---|---|---|
| **0** | 1215 | 4.9 |
| **1** | 770 | 7.0 |
| **2** | 207 | 11.6 |
| **3** | 10 | 30 |
| | | P<0.001 |

For peer review only

Supplementary Table 2
The predictive values of the elements of the APA score compared to the total score in the study and the validation population.

Table 2a The predictive values in the original study population

| | PPV | NPV | Sensitivity | Specificity |
|---|---|---|---|---|
| Aged >80 years | 7.9% | 94.2% | 32.1% | 74.7% |
| Previous Stroke | 11.9% | 93.9% | 7.1% | 96.4% |
| Age >80 years and Previous Stroke | 8.4% | 94.5% | 37.9% | 72.1% |
| APA score ≥2 | 12.4% | 94.6% | 19.3% | 91.2% |

Table 2b The predictive values in the validation population

| | PPV | NPV | Sensitivity | Specificity |
|---|---|---|---|---|
| Aged >80 years | 9.8% | 91.3% | 38.5% | 64.6% |
| Previous Stroke | 18.2% | 93.6% | 46.2% | 79.2% |
| Age >80 years and Previous Stroke | 13.3% | 91.4% | 15.4% | 90.0% |
| APA score ≥2 | 24.4% | 96.9% | 76.9% | 75.4% |

PPV Positive Predictive Value
NPV Negative Predictive Value
APA Composite of Age >80 years, Previous stroke, elevated ACR. A score of 2 or 3 are regarded as high risk

For peer review only

# TRIPOD Checklist: Prediction Model Development

| Section/Topic | Item | Checklist Item | Page |
|---|---|---|---|
| **Title and abstract** | | | |
| Title | 1 | Identify the study as developing and/or validating a multivariable prediction model, the target population, and the outcome to be predicted. | 1 |
| Abstract | 2 | Provide a summary of objectives, study design, setting, participants, sample size, predictors, outcome, statistical analysis, results, and conclusions. | 3 |
| **Introduction** | | | |
| Background and objectives | 3a | Explain the medical context (including whether diagnostic or prognostic) and rationale for developing or validating the multivariable prediction model, including references to existing models. | 6 |
| | 3b | Specify the objectives, including whether the study describes the development or validation of the model or both. | 7 |
| **Methods** | | | |
| Source of data | 4a | Describe the study design or source of data (e.g., randomized trial, cohort, or registry data), separately for the development and validation data sets, if applicable. | 7 |
| | 4b | Specify the key study dates, including start of accrual; end of accrual; and, if applicable, end of follow-up. | 7 |
| Participants | 5a | Specify key elements of the study setting (e.g., primary care, secondary care, general population) including number and location of centres. | 7 |
| | 5b | Describe eligibility criteria for participants. | 7 |
| | 5c | Give details of treatments received, if relevant. | 7-8 |
| Outcome | 6a | Clearly define the outcome that is predicted by the prediction model, including how and when assessed. | 9 |
| | 6b | Report any actions to blind assessment of the outcome to be predicted. | 9 |
| Predictors | 7a | Clearly define all predictors used in developing or validating the multivariable prediction model, including how and when they were measured. | 9-10 |
| | 7b | Report any actions to blind assessment of predictors for the outcome and other predictors. | 8 |
| Sample size | 8 | Explain how the study size was arrived at. | 9 |
| Missing data | 9 | Describe how missing data were handled (e.g., complete-case analysis, single imputation, multiple imputation) with details of any imputation method. | n/a |
| Statistical analysis methods | 10a | Describe how predictors were handled in the analyses. | 9-10 |
| | 10b | Specify type of model, all model-building procedures (including any predictor selection), and method for internal validation. | 9-10 |
| | 10d | Specify all measures used to assess model performance and, if relevant, to compare multiple models. | 9-10 |
| Risk groups | 11 | Provide details on how risk groups were created, if done. | |
| **Results** | | | |
| Participants | 13a | Describe the flow of participants through the study, including the number of participants with and without the outcome and, if applicable, a summary of the follow-up time. A diagram may be helpful. | 10 |
| | 13b | Describe the characteristics of the participants (basic demographics, clinical features, available predictors), including the number of participants with missing data for predictors and outcome. | 10 & t1 |
| Model development | 14a | Specify the number of participants and outcome events in each analysis. | 11 & t1 |
| | 14b | If done, report the unadjusted association between each candidate predictor and outcome. | n/a |
| Model specification | 15a | Present the full prediction model to allow predictions for individuals (i.e., all regression coefficients, and model intercept or baseline survival at a given time point). | 12 |
| | 15b | Explain how to the use the prediction model. | |
| Model performance | 16 | Report performance measures (with CIs) for the prediction model. | 13 |
| **Discussion** | | | |
| Limitations | 18 | Discuss any limitations of the study (such as nonrepresentative sample, few events per predictor, missing data). | 16 |
| Interpretation | 19b | Give an overall interpretation of the results, considering objectives, limitations, and results from similar studies, and other relevant evidence. | 14-15 |
| Implications | 20 | Discuss the potential clinical use of the model and implications for future research. | 15 |
| **Other information** | | | |
| Supplementary information | 21 | Provide information about the availability of supplementary resources, such as study protocol, Web calculator, and data sets. | ST1 |
| Funding | 22 | Give the source of funding and the role of the funders for the present study. | 19 |

We recommend using the TRIPOD Checklist in conjunction with the TRIPOD Explanation and Elaboration document.

For peer review only

BMJ Open

# The evaluation of microalbuminuria as a prognostic indicator after a TIA or minor stroke in an outpatient setting: The Prognostic Role of Microalbuminuria in TIA Evolution (ProMOTE) Study

| | |
|---|---|
| Journal: | *BMJ Open* |
| Manuscript ID | bmjopen-2020-043253.R2 |
| Article Type: | Original research |
| Date Submitted by the Author: | 28-May-2021 |
| Complete List of Authors: | Strain, W; University of Exeter, Diabetes and Vascular Research Centre; Royal Devon and Exeter NHS Foundation Trust, Academic Department of Healthcare for Older People<br>Elyas, Salim; University of Exeter, Diabetes and Vascular Research Centre; Royal Devon and Exeter NHS Foundation Trust, Academic Department of Healthcare for Older People<br>Wedge, Nicola; Royal Devon and Exeter NHS Foundation Trust, Academic Department of Healthcare for Older People<br>Mounce, Luke; University of Exeter, Institute of Health Research<br>Henley, W; University of Exeter Medical School, Institute of Health Research<br>James, Martin; Royal Devon and Exeter NHS Foundation Trust, Academic Department of Healthcare for Older People<br>Shore, Angela; University of Exeter Medical School, NIHR Exeter Clinical Research Facility |
| <b>Primary Subject Heading</b>: | Medical management |
| Secondary Subject Heading: | Diagnostics |
| Keywords: | Stroke medicine < INTERNAL MEDICINE, STROKE MEDICINE, Risk management < HEALTH SERVICES ADMINISTRATION & MANAGEMENT |
| | |

SCHOLARONE™
Manuscripts

*I, the Submitting Author has the right to grant and does grant on behalf of all authors of the Work (as defined in the below author licence), an exclusive licence and/or a non-exclusive licence for contributions from authors who are: i) UK Crown employees; ii) where BMJ has agreed a CC-BY licence shall apply, and/or iii) in accordance with the terms applicable for US Federal Government officers or employees acting as part of their official duties; on a worldwide, perpetual, irrevocable, royalty-free basis to BMJ Publishing Group Ltd ("BMJ") its licensees and where the relevant Journal is co-owned by BMJ to the co-owners of the Journal, to publish the Work in this journal and any other BMJ products and to exploit all rights, as set out in our licence.*

*The Submitting Author accepts and understands that any supply made under these terms is made by BMJ to the Submitting Author unless you are acting as an employee on behalf of your employer or a postgraduate student of an affiliated institution which is paying any applicable article publishing charge ("APC") for Open Access articles. Where the Submitting Author wishes to make the Work available on an Open Access basis (and intends to pay the relevant APC), the terms of reuse of such Open Access shall be governed by a Creative Commons licence – details of these licences and which Creative Commons licence will apply to this Work are set out in our licence referred to above.*

*Other than as permitted in any relevant BMJ Author's Self Archiving Policies, I confirm this Work has not been accepted for publication elsewhere, is not being considered for publication elsewhere and does not duplicate material already published. I confirm all authors consent to publication of this Work and authorise the granting of this licence.*

For peer review only

# The evaluation of microalbuminuria as a prognostic indicator after a TIA or minor stroke in an outpatient setting: The Prognostic Role of Microalbuminuria in TIA Evolution (ProMOTE) Study

Running Title: Predictive role of microalbuminuria after TIA and minor strokes

**W David Strain[1,2,4],**

**Salim Elyas[1,2],**

**Nicola Wedge[2,4],**

**Luke Mounce[3],**

**William Henley[3],**

**Martin A James[2],**

**Angela C Shore[1,4]**

1 Diabetes and Vascular Research Centre, University of Exeter Medical School. Barrack Road, Exeter EX2 5AX

2 Academic Department of Healthcare for Older People, Royal Devon & Exeter Hospital Barrack Road, Exeter EX2 5DW

3 University of Exeter, College of Medicine and Health, Exeter, UK

4 NIHR Exeter Clinical Research Facility

**Address for Correspondence:**

Dr David Strain

Diabetes and Vascular Research Centre

University of Exeter Medical School, Royal Devon & Exeter Hospital

Barrack Road, Exeter. EX2 5AX UK

**Email. d.strain@exeter.ac.uk**

**Tel: +441392 403058**

**Fax: +441392 403027**

For peer review only



For peer review only - http://bmjopen.bmj.com/site/about/guidelines.xhtml

| | | |
|---|---|---|
| **Word count** | Manuscript | 3168 |
| | Abstract | 334 |

**Disclosures**

All authors confirm that there are no conflicts of interest

For peer review only



For peer review only - http://bmjopen.bmj.com/site/about/guidelines.xhtml

**Abstract**:

**Objective**: Transient ischaemic attacks (TIA) and minor strokes are important risk factors for further vascular events. We explored the role of albumin-creatinine ratio (ACR) in improving risk prediction after a first event.

**Setting**: Rapid access stroke clinics in the UK

**Participants**. 2,202 patients attending with TIA or minor stroke diagnosed by the attending stroke physician, able to provide a urine sample to evaluate albumin creatinine ratio (ACR) using a near patient testing device.

**Primary and secondary outcomes**. Primary outcome was Major Adverse Cardiac Events (MACE; recurrent stroke, myocardial infarction, or cardiovascular death) at 90 days. The key secondary outcome was to determine whether urinary ACR could contribute to a risk prediction tool for use in a clinic setting.

**Results**: 151 MACE occurred in 144 participants within 90 days. Participants with a MACE had higher ACR than those without. A composite score awarding a point each for Age >80, Previous stroke/TIA and presence of microalbuminuria identified those at low and high risk. 90% of patients were at low risk (scoring 0 or 1). Their 90-day risk of a MACE was 5.7%. Of the remaining "high risk" population (scoring 2 or 3) 12.4% experienced MACE over 90 days ($p<0.001$ compared to low risk population). The need for acute admission in the first 7 days was two-fold elevated in the high-risk group compared to the low risk (3.23 vs. 1.43%; $p=0.05$). These findings were validated in an independent historic sample.

**Conclusion**: A risk score comprising age, previous stroke/TIA and microalbuminuria predicts future MACE events whilst identifying those at low risk of a recurrent event. This tool shows promise in the risk stratification of patients to avoid the admission of



For peer review only - http://bmjopen.bmj.com/site/about/guidelines.xhtml

low-risk patients whilst assigning intensive interventions such as dual anti-platelet therapy to those at highest risk.

**Keywords**:

Stroke,

TIA,

Microalbuminuria,

ACR,

Risk prediction.

For peer review only

## Article Summary

*Strengths and limitations of this study*

- The pragmatic design of this study, provides good generalisability to clinical practice
- The predictive role of the combination of urinary ACR in combination with very basic demographics would be able to reassure the 90% of the population that there was only approximately a 1 in 20 risk of an event recurring, whereas the higher risk population had a 1 in 8 chance of a MACE outcome
- The study is limited by the availability of data to that which was available to the physicians at the time of the stroke appointment. This limits the ability to determine mechanistic association between ACR and MACE outcome
- Further work is required to determine whether therapies that reduce ACR can modify the risk of subsequent MACE

For peer review only

**Introduction:**

Stroke is the second most common cause of death and a leading cause of disability worldwide[1]. Despite, or possibly because of, recent trends in reducing stroke mortality, the health and social disability burden of stroke is increasing[2]. After advancing age, transient ischaemic attacks (TIA) and minor strokes are the most important risk factors for recurrent stroke and predict long term mortality.[3,4] 23% of patients presenting with stroke have a history of TIA in the 3 months prior to the index event.[5] This is a key population to target for secondary prevention, but these patients represent less than 10% of all those who present to rapid access TIA clinics.[6,7] Notably, half of all completed strokes occur within the first week after TIA or minor stroke.[5]

Accurate identification of those patients presenting to TIA clinics with TIA or minor stroke who are most at risk for future events is important in order to i) intensify treatments such as giving dual anti-platelet therapy[8,9] ii) guide the need for urgent admission to facilitate the detection and urgent surgical correction of severe internal carotid artery (ICA) stenosis[10] iii) reassure patients with lowest risk.

Several risk stratification tools are already utilised in such clinics such as ABCD2[11,12] (awarding points for Age, presenting Blood pressure, Clinical features of unilateral weakness or aphasia, Duration of symptoms and Diabetes), California score[6] and imaging based scoring systems.[10,13] These tools lack optimal sensitivity and specificity.[12,14] Indeed some studies suggest patients with a 'low risk' ABCD2 score (less than 4/7) have similar 90 day stroke risk as patients deemed high risk with an ABCD2 score above 4/7,[15] whilst missing up to 40% of patients with severe ICA stenosis.[16] As such, recent consensus guidelines have advised against their use.[17-19]

For peer review only

Increased urinary albumin excretion rate (AER) has been shown to predict incident stroke and heart failure in people with diabetes. In the general population, AER predicts cardiovascular disease and mortality post-stroke independent of conventional cardiovascular risk factors such as hypertension, diabetes and smoking [20-23]. Urinary albumin creatinine ratio (ACR) is a well recognised proxy for urinary AER, and can now be assessed using simple and inexpensive point of care equipment. In an earlier pilot study of 142 patients with minor stroke/TIA,[24] we identified the potential role of urinary ACR in identifying those at highest risk of a recurrent event. Although statistically significant, this study was not large enough to explore potential confounding. We therefore performed a definitive study to assess whether urinary ACR improved the risk stratification of patients presenting with TIA or minor stroke to UK acute stroke clinics.

**Methods:**

Patients diagnosed with suspected minor stroke/TIA by a consultant stroke physician, attending a rapid access clinics in one of 12 UK hospitals were recruited at the end of their clinic consultation. As these clinics are rapid access, often within the first 24 hours of symptoms, it was impossible to accurately differentiate between TIA (with symptoms less than 24 hours) and minor stroke in all cases. Only individuals with events that had occurred within the previous month were included in the study. Routine clinical care, including urgent revascularisation in those with severe ICA stenosis (> 50% diameter stenosis by the North American Symptomatic Carotid Endarterectomy Trial (NASCET) method,[25] was initiated prior to enrollment. If a cardioembolic source was identified, anticoagulants were initiated according to local protocols. After written,



For peer review only - http://bmjopen.bmj.com/site/about/guidelines.xhtml

informed consent was obtained in the clinic, demographics, including age, sex, height, weight, past medical history and ABCD2 score were recorded. Times from onset of symptoms to assessment and enrollment in the study were documented. A clean specimen of urine was collected from participants in the clinic and tested with a Unistix dipstick and, if there was no indication of urinary tract infection (presence of any two of leucocytes, nitrites and/or protein), ACR was measured using a point of care analyzer (The Afinion™ AS100 Analyzer, Axis Shield, Dundee, UK). This system uses an immunometric membrane flow-through principle for albumin measurement and an enzymatic colorimetric test for creatinine quantification[26] and reports an ACR in approximately 5 minutes within the range 0.1 to 140 mg/mmol with a coefficient of variation of 4.6-6%.[26] Participants with values of less than 0.1 mg/mmol were recorded as having the lowest recordable value of ACR (0.1 mg/mmol).

Participants were followed up by telephone on day 7, 30 and 90, and any history of any further vascular events, hospitalisation or death was obtained. If contact with participants was not possible initially, the presence or absence of further events was verified during additional attempts at contact or during future follow up. If no further contact was possible with the participant (e.g. in the case of significant stroke or death), medical history was collected from the next of kin and verified from the primary care physician and hospital records. All reported clinical events were adjudicated according to standardised diagnostic criteria by a data monitoring committee including two independent stroke physicians, blinded to the ACR, by examination of clinical records if they attended hospital, or their primary care physician, or by verifying their clinical symptoms from the research records if patients did not seek further medical advice. In the event of disagreement, a third independent stroke physician acted as adjudicator.



For peer review only - http://bmjopen.bmj.com/site/about/guidelines.xhtml

The primary outcome was to determine the utility of microalbuminuria in predicting the time to first major adverse cardiovascular event (MACE; recurrent stroke, myocardial infarction or death) within 90 days. Secondary outcomes were time to explore the predictive role of microalbuminuria on recurrent stroke, total mortality (even if these were not the first MACE event), the presence of first MACE within 7 days, and the need for hospitalisation within the first 7 days. All events were adjudicated blind to urinary albumin creatinine ratio by an independent data monitoring committee.

*Ethics Statement*

The study protocol was approved by the national research ethics committee (approval 14/EE/1106). All participants provided written informed consent prior to enrolment and confirmed their willingness to continue participation at each telephone consultation.

*Patient and public involvement*

Patients were involved in the design and conduct of this research. During the feasibility stage, priority of the research question, and methods of recruitment were informed by discussions with patients through a focus group session and three structured interviews. During the trial, two patients joined the independent trial steering committee. Once the trial has been published, participants will be informed of the results through a dedicated newsletter suitable for a non-specialist audience, led by one of the patients on the steering committee.

*Statistical analysis:*


For peer review only - http://bmjopen.bmj.com/site/about/guidelines.xhtml

Data were treated as continuous variables wherever possible to maximize power. All normally distributed data are presented as mean ± standard deviation (SD). Skewed data were appropriately transformed and presented as geometric means (with 95% confidence intervals [CI]). Statistical significance for categorical variables was calculated using the chi-squared test and the Student's t-test for continuous variables. Independence of ACR (as a risk predictor) from diabetes and other measures of the ABCD2 score was assessed using logistic regression. Microalbuminuria was defined using the currently accepted clinical thresholds for microalbuminuria used in people with diabetes 3.5mg/mol for women and 2.5mg/mol for men. Identification of independent predictors of recurrent MACE events was performed by backwards stepwise logistic regression, commencing with all information available for the complete dataset, representing all information that would routinely be available to the clinician in the clinic setting. Where multiple related measures were available (e.g. systolic, diastolic and mean arterial blood pressure) the variable that accounted for the greatest degree of variance in a univariate analysis was included. Fractional polynomial regression modeling was used to identify sex-specific thresholds of ACR to define "microalbuminuria" in the context of stroke risk stratification. Prediction of events were calculated using a Cox proportional hazard ratio with time to first MACE as the primary outcome. In keeping with the recommendations of Cupples[27] and Rothman[28] the measured significance of the variables of interest is reported without adjustment for multiple testing. Statistical significance was considered at $p<0.05$. Statistical analysis was performed using Stata SE 14.2 (Mac version: Statacorp ltd Texas).

**Results:**



For peer review only - http://bmjopen.bmj.com/site/about/guidelines.xhtml

Two thousand, four hundred and eight patients with a diagnosis of definite or probable minor stroke/TIA were recruited; 149 participants were subsequently excluded after the diagnosis was revised to that of a stroke mimic, 8 withdrew consent and a further 49 (2%) were excluded due to intercurrent urinary tract infection rendering the assessment of ACR invalid. No patient was lost to follow-up. Baseline characteristics are presented in table 1. Of the 2,202 included in the final analysis, most were male (64.9%). All patients were commenced on secondary prevention with appropriate antiplatelet or anticoagulant therapy, and an appropriately dosed statin in accordance with contemporaneous national guidelines.

Over 90 days, 151 primary outcome events (MACE) occurred in 144 participants (6.7% of patients) including 8 cardiovascular deaths. All MACE events were atherosclerotics with no haemorrhagic events occuring in the 3 months. There were also 8 non-cardiovascular deaths. Within 7 days there were 38 MACE in 36 participants. Those with MACE were more likely to have had previous stroke/TIA, however, with this exception, there were no significant differences in routinely collected clinic data (Table 1). There was no difference in mean ABCD2 score or the proportion of participants with a 'high risk' score (4/7 or more) between those who did and did not have events by 90 days. When looking exclusively at the 1374 participants identified with a "high risk" ABCD2 score (63% of the study sample; 93% male), a high score did not distinguish those at high risk of MACE event compared to those with an ABCD2 <4 (HR 1.15 (0.82-1.63); p=0.4), nor did a low risk score exclude an event, with only 37.7% of people who did not have experience a MACE outcome scoring below 4. Point-of-care ACR was higher in those participants with a primary outcome event at 90 days compared to those without (2.27 (1.92-2.68) vs. 1.75 (1.68–1.83) mg/mmol


For peer review only - http://bmjopen.bmj.com/site/about/guidelines.xhtml

respectively; p=0.002). This distinction remained after adjustment for age and sex (adjusted ACR 2.40 (2.04-2.83) vs. 1.88 (1.80 – 1.96), p=0.004. Figure 1).

*Evaluation of the prognostic role of microalbuminuria alone*

Fractional polynomial regression modelling identified the sex-specific threshold of risk for ACR aligned with the currently accepted thresholds for microalbuminuria used in people with diabetes of 2.5mg/mmol for men and 3.5mg/mol for women. These thresholds identified 562 participants (25.4% of the study sample; 75% male) with microalbuminuria. These participants were more likely than those with a low ACR to experience a primary outcome event (Hazard Ratio 1.66 (95% CI 1.19 - 2.34); p=0.003) and had a greater than 5-fold increase in 90-day mortality (HR 5.44 (1.86 – 15.90); p=0.0003). Alone, however, microalbuminuria did not have sufficient positive or negative predictive value to justify its clinical utility (Positive predictive value – 9.1%, Specificity 75.4%).

*Generating a composite risk score for 90-day risk stratification*

In a stepwise multivariate model, the only independent predictors of MACE were elevated ACR, age greater than 80 and history of previous stroke or TIA. Similar variance within the model was explained microalbuminuria (i.e. an ACR >2.5 and 3.5mg/mol respectively in men and women) as being older than 80 years and previous history of stroke/TIA, so they were afforded equal weighting. This generated a 4-point scale between 0 and 3 (termed the 'APA' score representing Age, Prior stroke or TIA and elevated ACR), with the lowest risk participants having a score of 0 (Supplementary Table 1). There was a sequential increase in risk with increasing score such that those with a score of 0 having a 90-day risk of 4.86% rising to a 30% risk for



For peer review only - http://bmjopen.bmj.com/site/about/guidelines.xhtml

those with the highest score (supplementary table 2). The population was then divided into a low risk group scoring 0 or 1 or high risk scoring 2 or 3. 1,985 (90.1%) of participants were identified as low risk compared to 217 (9.9%) in the high-risk group. When comparing the positive predictive value (PPV) sensitivity and specificity of the APA score compared to the previously available parameters of age and previous stroke demonstrated a clinically meaningful improvement over each measure on its own (Supplementary table 3a). Compared to using previous stroke alone, APA score had a similar positive predictive value (12.4% for APA score vs 11.9% for previous stroke) and specificity (91.2% vs 96.4%, respectively), however superior sensitivity (19.3% vs 7.1%, respectively). Sensitivity was improved by using a composite of previous stroke and being over the age of 80 years, however this was at the expense of a reduction of positive predictive value to 8.4%.

The high-risk group was older, with higher systolic blood pressure and pulse pressure compared to the low-risk group (table 2). The 90-day risk of a MACE outcome was 5.7% in the low-risk group, versus 12.4% in the high-risk group ($p<0.001$), translating to a hazard ratio of 2.12 (95% CI 1.38 – 3.25) for the high-risk score compared to low-risk group ($p<0.001$; Figure 2). This was predominantly driven by an increase in recurrent stroke/TIA (3.3% vs. 8.8%; HR 2.67(1.58-4.51); $p<0.001$). This difference was apparent within 7 days of the clinic, such that those with a high APA score had a 2.2-fold increased risk of a recurrent event.

*Validation of APA score on a historic sample*

In order to validate the APA score, we applied the tool to the individuals who participated in the pilot study but did not contribute their data to this dataset. Details of


For peer review only - http://bmjopen.bmj.com/site/about/guidelines.xhtml

this pilot study have been published elsewhere [24]. In brief, the pilot study recruited 139 participants over 9 months from one of the centres involved in the definitive study. In this population, there were 13 recurrent events (9.35%) within the 90 days, 9 of which occurred in the first 7 days after attendance to the stroke clinic (Table 3). The APA risk score replicated a similar distribution of number of participants at low risk (73%) and high risk (27%) as in the larger study. Compared to the component parts, the APA score had superior positive predictive value (24.4% vs 18.2% for Previous Stroke or 13.3% for previous stroke and being aged >80 years) and superior sensitivity (76.9% vs 46.2% vs 15.4% respectively: supplementary table 3b). At 90 days, 5.9% of the low APA group had recurrent events compared to 18.4% of the in the high-APA group (p=0.02). Again, within the first 7 days this was apparent with only 1.9% of the low APA group experiencing the primary outcome, compared to 13.2% in the high APA group (p=0.04).

**Discussion:**

In this large UK multicentre prospective study with blinded outcome adjudication, we have demonstrated the utility of an elevated ACR, measured using a simple point-of-care analyser, in combination with simple clinical data (age and previous history of stroke or TIA) in the prediction of major adverse cardiovascular events and death. This differentiates between low and high-risk individuals. Specifically, over 90% of individuals were identified as low risk with less than a 1.5% risk of a major adverse cardiovascular event or death over the next 7 days, increasing to 5.7% by 90 days, whereas 9.9 % of the population were identified at high risk with a 12.4% event rate over 90 days. Although only a small number, our findings were validated in a separate historic population who participated in the pilot study.


For peer review only - http://bmjopen.bmj.com/site/about/guidelines.xhtml

The utility of APA scoring may go beyond simply guiding conversations regarding prognosis in a clinic setting. The POINT[8] and CHANCE[9] studies demonstrated the benefit of dual anti-platelet therapy in the first 21 days after high risk TIA. There was, however, a 0.5% absolute increase in major intracranial haemorrhage. We would propose that an assessment of the APA score could be evaluated as a stratification tool to determine if it predicts those who benefit the most. In those with a low-risk APA score, even the 25% relative risk reduction in the POINT trial would result in only a marginally beneficial risk:benefit ratio, assuming that microalbuminuria is a predictor of thromboembolic disease processes. Those with a high APA score, however, would likely benefit from dual-antiplatelet therapy with a number need to treat of less than 30 in order to prevent an event. This suggestion, however, does require confirmation in an independent study. The APA score also gives additional information that is of use in the more acute setting. Over the first 7 days, a low-risk APA score had a negative predictive value of >98.5% for the risk of a MACE - a number very similar to the predictive effect of troponin when evaluating suspected cardiac chest pain.[29]

When considering the populations identified by the APA score, there are similarities to the ABCD2 score. Indeed, the demographics of the high-risk group were older, with higher blood pressure and a trend towards more diabetes. We would suggest, that microalbuminuria represents vascular susceptibility to the adverse consequences of hypertension and diabetes rather than simply an indication of the presence of hypertension and /or diabetes in general. This would explain why the APA score predicted further MACE and total mortality at 7 and 90 days, whereas in our large sample the ABCD2 score did not.



For peer review only - http://bmjopen.bmj.com/site/about/guidelines.xhtml

Elevated ACR is a recognised marker of generalised endothelial and microvascular dysfunction. The increased filtration of albumin through the renal glomerular filtration barrier is thought to be due to changes in the chemical and physical properties of this endothelial barrier and its glycocalyx.[30-32] The mechanism explaining the association between microalbuminuria and incident cardiovascular events is thought to depend on its role as a marker of systemically increased vascular permeability and altered homeostasis, coagulation and endothelial function.[33-38] However, after acute ischaemic events such as myocardial infarction, or as demonstrated here TIA or minor stroke, it is likely that elevated urinary ACR is, at least in part, dependent on the systemic inflammatory response to the original insult.

Whilst unlikely to be mechanistically involved in the progression of disease, the question as to whether it is a good surrogate of therapeutic effect remains. Improving systemic inflammation, such as through the pleiotropic effect of statins, has been associated with simultaneous improvements in urinary albumin excretion and CV event rates in patients with elevated high sensitivity C-reactive protein (hsCRP).[39-41] Further studies are needed to determine whether microalbuminuria after acute stroke represents a therapeutic target amenable to treatment, and if reductions in microalbuminuria are associated with reductions in the vascular events they predict.

*Limitations and strengths of study*

Recurrent event rate was lower than anticipated from our pilot study and contemporaneous clinical trials such as CHANCE (11.7% in the control arm),[9] although are comparable with the recently-reported randomised controlled POINT trial (6%).[8] In usual practice it is usually 24-48 hours between the index event and clinic attendance.


For peer review only - http://bmjopen.bmj.com/site/about/guidelines.xhtml

Given that data from previous studies has demonstrated the highest risk occurs in the first 48 hours, it is likely that some events will have occurred between the index event and attendance in the clinic in our study.[42] The pragmatic design of the study, however, is also a strength given that the predictive role highlights those that may benefit from dual anti-platelet therapy or hospitalisation when the patient is seen in the acute stroke clinic.

There is no consensus on the gold standard for diagnosing TIA in a rapid access stroke clinic.[43] In order to maintain the generalisability of the study, all attendees of the stroke clinic that were diagnosed with a probable or definite TIA were invited to participate. Only a small number of patients were subsequently withdrawn as being stroke mimics. Outcome events were rigorously adjudicated against published diagnostic criteria to ensure consistency and reliability.

Finally, the use of a single urinary ACR sample obtained during the clinic assessment using a near patient testing kit is not as robust as a sample tested in a central laboratory. Whilst there is significant diurnal variability in urinary AER within individuals, potentially limiting the scientific or mechanistic merit of the study compared to studies using overnight or 24-hour urine collection, the pragmatic design makes this study more applicable to general clinic populations. Further, the relatively low cost of the near patient test kit makes it accessible in most emergency settings, in primary and secondary care.

**Conclusion:**

For peer review only



For peer review only - http://bmjopen.bmj.com/site/about/guidelines.xhtml

We have demonstrated for the first time the potential utility of a single point of care test of urinary ACR in patients presenting with TIA and minor stroke as a prediction tool to assist triaging patients. When used in combination with patient age and previous history of stroke or TIA, this generated a risk stratification score (the APA score: Age>80, Past history of stroke/TIA, and elevated ACR) that could reliably identify the large proportion of patients with a low risk of recurrent vascular event over the next 90 days. The value of ACR that is most predictive is aligned with the conventional definition of microalbuminuria used in diabetes, suggesting that in clinical practice the existing technology for evaluating microalbuminuria including urine dipsticks may be used. A high APA score was associated with a doubling of risk of MACE outcome and a five fold increase in mortality. This APA score was validated in an independent population. We believe the APA score may represent a practical tool for clinicians engaged in the acute ambulatory assessment of TIA and minor stroke, to assist in the assessment of risk:benefit ratio for the use dual anti-platelet therapy or to admit for more urgent investigation. Further work is required to determine whether the increase in ACR represents a therapeutic target or solely a prognostic indicator.

For peer review only



For peer review only - http://bmjopen.bmj.com/site/about/guidelines.xhtml

**Contributorship statement**

WDS & ACS conceived and designed the study, attained funding, were involved in the management of the trial. WDS produced the first draft of the manuscript. SE, NW & MAJ contributed to the design and management of the trial. LM &WH provided the statistical support for the management of the trial and the analysis of the outcomes. All authors have reviewed the final manuscript prior to submission.

**Competing interests**

No authors report any competing interests relevant to this manuscript.

**Funding**

This study was funded by a NIHR Research for Patient Benefit grant (PB-PG-1112-29069) and supported by the NIHR Exeter Clinical Research Facility.

**Data sharing statement**

Anonymised data is available from the corresponding author on reasonable request.

**Acknowledgements**

WDS is supported by the NIHR Exeter Clinical Research Facility. WDS and MAJ is supported by the NIHR SW Peninsula Applied Research Collaboration. This manuscript does not necessarily reflect the views of the NIHR, the Exeter Clinical Research Facility, the NHS or the UK Department of Health.

For peer review only

**References:**


1. Feigin VL, Norrving B, Mensah GA. Global Burden of Stroke. *Circ Res.* 2017;120(3):439-448.
2. Henssge U. National Sentinel Stroke Clinical Audit 2010. In: Royal College of Physicians, ed2011.
3. Burn J, Dennis M, Bamford J, Sandercock P, Wade D, Warlow C. Long-term risk of recurrent stroke after a first-ever stroke. The Oxfordshire Community Stroke Project. *Stroke; a journal of cerebral circulation.* 1994;25(2):333-337.
4. Hankey GJ. Long-term outcome after ischaemic stroke/transient ischaemic attack. *Cerebrovascular diseases (Basel, Switzerland).* 2003;16 Suppl 1:14-19.
5. Rothwell PM, Warlow CP. Timing of TIAs preceding stroke: time window for prevention is very short. *Neurology.* 2005;64(5):817-820.
6. Johnston SC, Gress DR, Browner WS, Sidney S. Short-term prognosis after emergency department diagnosis of TIA. *Jama.* 2000;284(22):2901-2906.
7. Hill MD, Yiannakoulias N, Jeerakathil T, Tu JV, Svenson LW, Schopflocher DP. The high risk of stroke immediately after transient ischemic attack: a population-based study. *Neurology.* 2004;62(11):2015-2020.
8. Johnston SC, Easton JD, Farrant M, et al. Clopidogrel and Aspirin in Acute Ischemic Stroke and High-Risk TIA. *N Engl J Med.* 2018;379(3):215-225.
9. Wang Y, Wang Y, Zhao X, et al. Clopidogrel with aspirin in acute minor stroke or transient ischemic attack. *N Engl J Med.* 2013;369(1):11-19.
10. Kiyohara T, Kamouchi M, Kumai Y, et al. ABCD3 and ABCD3-I scores are superior to ABCD2 score in the prediction of short- and long-term risks of stroke after transient ischemic attack. *Stroke; a journal of cerebral circulation.* 2014;45(2):418-425.


For peer review only

11. Rothwell PM, Giles MF, Flossmann E, et al. A simple score (ABCD) to identify individuals at high early risk of stroke after transient ischaemic attack. *Lancet.* 2005;366(9479):29-36.

12. Bhatt A, Jani V. The ABCD and ABCD2 Scores and the Risk of Stroke following a TIA: A Narrative Review. *ISRN Neurol.* 2011;2011:518621.

13. Ay H, Arsava EM, Johnston SC, et al. Clinical- and imaging-based prediction of stroke risk after transient ischemic attack: the CIP model. *Stroke; a journal of cerebral circulation.* 2009;40(1):181-186.

14. Giles MF, Rothwell PM. Systematic review and pooled analysis of published and unpublished validations of the ABCD and ABCD2 transient ischemic attack risk scores. *Stroke; a journal of cerebral circulation.* 2010;41(4):667-673.

15. Amarenco P, Labreuche J, Lavallée PC. Patients With Transient Ischemic Attack With ABCD2 <4 Can Have Similar 90-Day Stroke Risk as Patients With Transient Ischemic Attack With ABCD2 ≥4. *Stroke; a journal of cerebral circulation.* 2012;43(3):863-865.

16. Amarenco P, Labreuche J, Lavallee PC. Patients with transient ischemic attack with ABCD2 <4 can have similar 90-day stroke risk as patients with transient ischemic attack with ABCD2 >/=4. *Stroke; a journal of cerebral circulation.* 2012;43(3):863-865.

17. National Institute of Health and Care Excellence. Stroke and transient ischaemic attack in over 16s: diagnosis and initial management; NICE guideline [NG128]. National Institute of Health and Care Excellence. Published 2019. Updated May 2019. Accessed 10/11/20, 2019.

18. Powers WJ, Rabinstein AA, Ackerson T, et al. 2018 Guidelines for the Early Management of Patients With Acute Ischemic Stroke: A Guideline for


For peer review only - http://bmjopen.bmj.com/site/about/guidelines.xhtml

Healthcare Professionals From the American Heart Association/American Stroke Association. *Stroke.* 2018;49(3):e46-e110.

19. Ahmed N, Audebert H, Turc G, et al. Consensus statements and recommendations from the ESO-Karolinska Stroke Update Conference, Stockholm 11–13 November 2018. *European Stroke Journal.*0(0):2396987319863606.

20. Gerstein HC, Mann JF, Yi Q, et al. Albuminuria and risk of cardiovascular events, death, and heart failure in diabetic and nondiabetic individuals. *JAMA.* 2001;286(4):421-426.

21. Hillege HL, Fidler V, Diercks GF, et al. Urinary albumin excretion predicts cardiovascular and noncardiovascular mortality in general population. *Circulation.* 2002;106(14):1777-1782.

22. Yuyun MF, Khaw KT, Luben R, et al. Microalbuminuria, cardiovascular risk factors and cardiovascular morbidity in a British population: the EPIC-Norfolk population-based study. *Eur J Cardiovasc Prev Rehabil.* 2004;11(3):207-213.

23. Strain WD, Shore AC, Melzer D. Albumin:creatinine ratio predicts mortality after stroke: analysis of the Third National Health and Nutrition Examination Survey. *J Am Geriatr Soc.* 2010;58(12):2434-2435.

24. Elyas S, Shore AC, Kingwell H, et al. Microalbuminuria could improve risk stratification in patients with TIA and minor stroke. *Ann Clin Transl Neurol.* 2016;3(9):678-683.

25. Morgenstern LB, Fox AJ, Sharpe BL, Eliasziw M, Barnett HJ, Grotta JC. The risks and benefits of carotid endarterectomy in patients with near occlusion of the carotid artery. North American Symptomatic Carotid Endarterectomy Trial (NASCET) Group. *Neurology.* 1997;48(4):911-915.



For peer review only - http://bmjopen.bmj.com/site/about/guidelines.xhtml

26. Kvam C. Afinion ACR Scientific Poster AACC 2007. Axis-Shield PoC AS. http://www.afinion.net/publications. Published 2007. Accessed.

27. Cupples LA, Heeren T, Schatzkin A, Colton T. Multiple testing of hypotheses in comparing two groups. *Ann Intern Med.* 1984;100(1):122-129.

28. Rothman KJ. No adjustments are needed for multiple comparisons. *Epidemiology.* 1990;1(1):43-46.

29. Gualandro DM, Puelacher C, LuratiBuse G, et al. Comparison of high-sensitivity cardiac troponin I and T for the prediction of cardiac complications after non-cardiac surgery. *Am Heart J.* 2018;203:67-73.

30. Haraldsson B, Jeansson M. Glomerular filtration barrier. *Curr Opin Nephrol Hypertens.* 2009;18(4):331-335.

31. Singh A, Satchell SC. Microalbuminuria: causes and implications. *Pediatr Nephrol.* 2011;26(11):1957-1965.

32. Gansevoort RT, Lambers H, Witte EC. Methodology of screening for albuminuria. *Nephrology Dialysis Transplantation.* 2007;22(8):2109-2111.

33. Barzilay JI, Peterson D, Cushman M, et al. The relationship of cardiovascular risk factors to microalbuminuria in older adults with or without diabetes mellitus or hypertension: the cardiovascular health study. *Am J Kidney Dis.* 2004;44(1):25-34.

34. Valmadrid CT, Klein R, Moss SE, Klein BE. The risk of cardiovascular disease mortality associated with microalbuminuria and gross proteinuria in persons with older-onset diabetes mellitus. *Arch Intern Med.* 2000;160(8):1093-1100.

35. Dinneen SF, Gerstein HC. The association of microalbuminuria and mortality in non-insulin-dependent diabetes mellitus. A systematic overview of the literature. *Arch Intern Med.* 1997;157(13):1413-1418.



For peer review only - http://bmjopen.bmj.com/site/about/guidelines.xhtml

36. Festa A, D'Agostino R, Howard G, Mykkanen L, Tracy RP, Haffner SM. Inflammation and microalbuminuria in nondiabetic and type 2 diabetic subjects: The Insulin Resistance Atherosclerosis Study. *Kidney Int.* 2000;58(4):1703-1710.
37. Asselbergs FW, de Boer RA, Diercks GF, et al. Vascular endothelial growth factor: the link between cardiovascular risk factors and microalbuminuria? *Int J Cardiol.* 2004;93(2-3):211-215.
38. Stehouwer CD, Gall MA, Twisk JW, Knudsen E, Emeis JJ, Parving HH. Increased urinary albumin excretion, endothelial dysfunction, and chronic low-grade inflammation in type 2 diabetes: progressive, interrelated, and independently associated with risk of death. *Diabetes.* 2002;51(4):1157-1165.
39. Ridker PM, Danielson E, Fonseca FA, et al. Rosuvastatin to prevent vascular events in men and women with elevated C-reactive protein. *N Engl J Med.* 2008;359(21):2195-2207.
40. Abe M, Maruyama N, Okada K, Matsumoto S, Matsumoto K, Soma M. Effects of lipid-lowering therapy with rosuvastatin on kidney function and oxidative stress in patients with diabetic nephropathy. *J Atheroscler Thromb.* 2011;18(11):1018-1028.
41. Girardi JM, Farias RE, Ferreira AP, Raposo NR. Rosuvastatin prevents proteinuria and renal inflammation in nitric oxide-deficient rats. *Clinics (Sao Paulo).* 2011;66(8):1457-1462.
42. Coull AJ, Rothwell PM. Underestimation of the early risk of recurrent stroke: evidence of the need for a standard definition. *Stroke; a journal of cerebral circulation.* 2004;35(8):1925-1929.

For peer review only

43. Graham C, Bailey D, Hart S, et al. Clinical diagnosis of TIA or minor stroke and prognosis in patients with neurological symptoms: A rapid access clinic cohort. *PLoS One.* 2019;14(3):e0210452.

For peer review only

**Table Legends (ProMOTE Study):**

**Table 1**: Sample characteristics of total population and population stratified by occurrence of recurrent major adverse cardiovascular events (MACE) at 90 days

Figures presented as mean (SD) for continuous variable except ACR where geometric mean (Inter-quartile range) presented; % for categorical data

P for continuous data by Student's t-test

P for categorical data by Chi Squared test

BMI = Body Mass Index,

ABCD2 (A=Age, B=BP, C=Clinical features, D= Duration of symptoms + Diabetes),

ACR Albumin Excretion Rate

*Note*: Total Cholesterol was collected from routine clinical practice records therefore is not available for all participants

**Table 2**: Occurrence of events by APA risk score group (mean ± SD for continuous variable, p for ANOVA; % for categorical data, p for Chi-Squared)

**Table 3:** Occurrence of events by APA risk score group in independent validation population (mean ± SD for continuous variable, p for ANOVA; % for categorical data, p for Chi-Squared)

For peer review only

Table 1: Sample characteristics of total population and population stratified by occurrence of recurrent vascular events or death (MACE) at 90 days.

| Characteristic | Total population | No event by 90 days | Event at 90 days |
|---|---|---|---|
| N | 2202 | 2058 | 144 |
| Age (years) | 70.9 (11.7) | 70.9 (11.6) | 72.2 (12.2) |
| Sex (%male) | 64.8 | 64.7 | 67.9 |
| Height (m) | 1.70 (0.10) | 1.70 (0.10) | 1.71 (0.9) |
| Weight (kg) | 79.8 (17.1) | 79.9 (17.0) | 79.7 (17.7) |
| BMI (kg/m$^2$) | 27.6 (5.2) | 27.6 (5.2) | 27.2 (4.9) |
| Blood pressure | | | |
| Systolic (mmHg) | 144.1 (20.8) | 144.2 (20.9) | 145.7 (21.7) |
| Diastolic (mmHg) | 81.8 (12.6) | 81.7 (12.6) | 83.8 (13.1) |
| Total Cholesterol§ | (n = 1,466) | (n=1,374) | (n=92) |
| (mmol/L) | 5.02 (2.57) 194 | 5.03 (2.63) | 4.75 (1.24) |
| (mg/dl) | 194(99) | 195 (102) | 183 (48) |
| Diabetes (%) | 15.7 | 15.9 | 12.1 |
| Previous Stroke (%) | 4.0 | 3.5 | 10.7* |
| ABCD2 | | | |
| Mean score out of 7 | 4.0 (1.4) | 4.0 (1.4) | 4.1 (1.4) |
| Proportion >4 (%) | 62.5 | 62.3 | 65.0 |
| "High Risk" | | | |
| ACR (mg/mmol)† | 1.79(0.9-2.9) | 1.75(1.69-1.83) | 2.27(1.92-2.68)** |
| ACR (mg/g)† | 15.8(8.0-25.6) | 15.5 (14.9-16.2) | 20.1 (17.0-23.7)** |

§ These were collected from patient records where clinician had requested the test and therefore only available in a subset of the population

† Geometric mean (95% CI)

*P<0.0001 compared to those with no events

** p=0.002 compared to those with no events



For peer review only - http://bmjopen.bmj.com/site/about/guidelines.xhtml

Table 2: Occurrence of events by APA risk score group (mean ± SD for continuous variable, p for ANOVA; % for categorical data, p for Chi-Squared)

| | Low Risk | High Risk | p |
|---|---|---|---|
| n | 1,985 | 217 | |
| Sex (% Male) | 64.5 | 68.2 | 0.2 |
| Age (years) | 69.6 (11.3) | 84.0 (5.0) | <0.0001 |
| Blood pressure (mmHg) | | | |
| Systolic | 143 (20.4) | 150.5 (23.3) | <0.0001 |
| Diastolic | 82 (12.3) | 79 (11.6) | 0.0007 |
| Weight (kg) | 80.3 (17.1) | 75.1 (16.2) | <0.0001 |
| Diabetes | 15.3 | 18.9 | 0.2 |
| MACE at 90 days (%) | | | |
| All events | 5.7 | 12.4 | <0.0001 |
| Cerebrovascular event | 3.2 | 8.8 | <0.0001 |
| Cardiovascular Mortality | 0.1 | 1.4 | <0.001 |
| All Cause Mortality | 0.4 | 2.3 | 0.001 |
| MACE at 7 days (%) | 1.46 | 3.23 | 0.05 |

For peer review only

**Table 3**: Occurrence of events by APA risk score group in independent validation population (mean ± SD for continuous variable, p for ANOVA; % for categorical data, p for Chi-Squared)

| | Low Risk | High Risk | p |
|---|---|---|---|
| n | 101 | 38 | |
| Sex (% Male) | 51.5 | 71.5 | 0.04 |
| Age (years) | 72.6 (10.7) | 81.6 (6.1) | 0.001 |
| Blood pressure (mmHg) | | | |
| Systolic | 142.6 (20.2) | 147.3 (26.6) | 0.3 |
| Diastolic | 77.7 (9.8) | 77.4 (10.8) | 0.9 |
| Weight (kg) | 76.2 (16.4) | 75.0 (15.4) | 0.7 |
| | | | |
| Recurrent event at 90 days (%) | | | |
| MACE event | 5.9 | 18.4 | 0.02 |
| | | | |
| Recurrent event at 7 days (%) | | | |
| MACE events* | 1.9 | 13.2 | 0.04 |

* MACE = Major Adverse Cardiovascular Event – Recurrent stroke or TIA, Myocardial infarction or cardiovascular death.

For peer review only



For peer review only - http://bmjopen.bmj.com/site/about/guidelines.xhtml

**Figure 1:** Age and sex adjusted Albumin Creatinine Ratio (Mean +/-95% CI) stratified by the occurrence of a Major Adverse Cardiac Event (MACE) in the first 90 days post initial event

MACE – Major Adverse Cardiovascular event = recurrent cerebrovascular event, myocardial infarction or cardiovascular death

**Figure 2**: Time to first Major Adverse Cardiac Event (MACE) stratified by APA (Age, Previous cerebrovascular event, High ACR) risk score. Event rate in high risk (n=217) 12.44% vs. low risk (n=1985) 5.69%, HR 2.12 (1.38 – 3.25) p<0.001

For peer review only

For peer review only



For peer review only - http://bmjopen.bmj.com/site/about/guidelines.xhtml

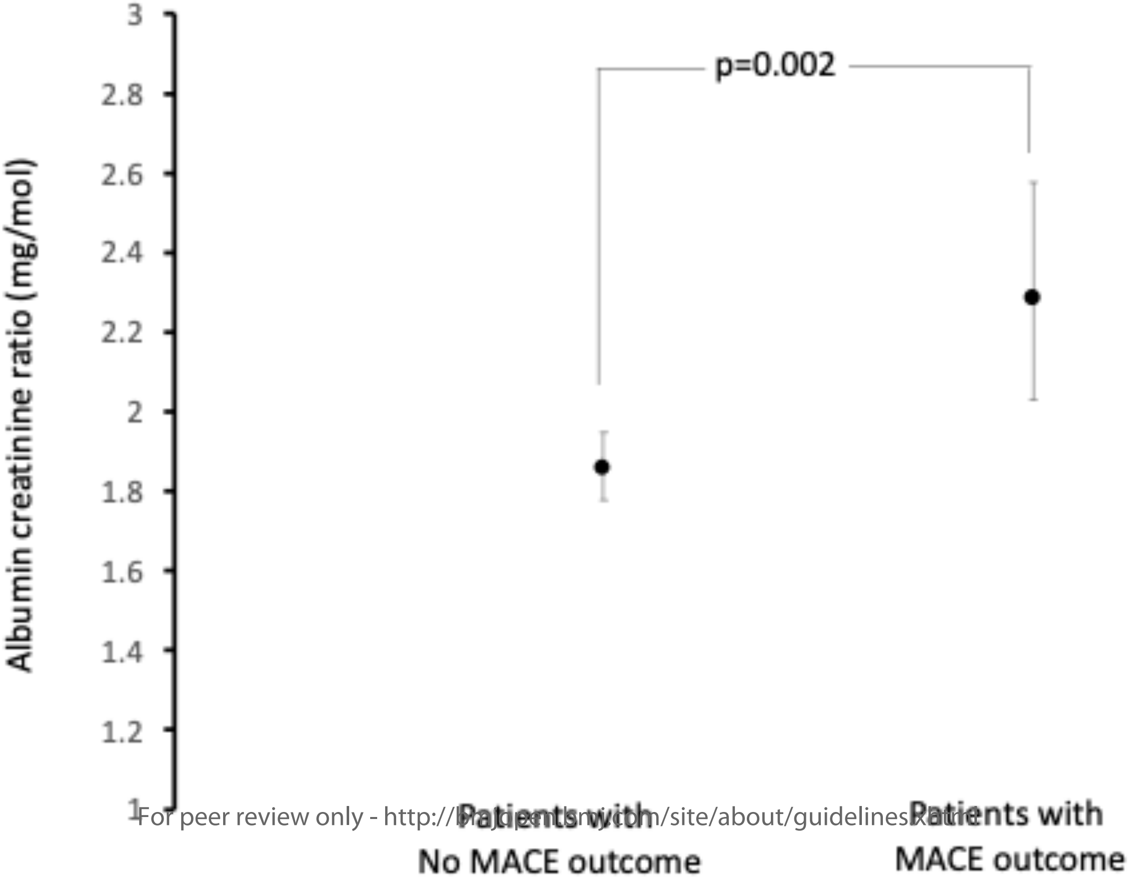


For peer review only - http://bmjopen.bmj.com/site/about/guidelines.xhtml

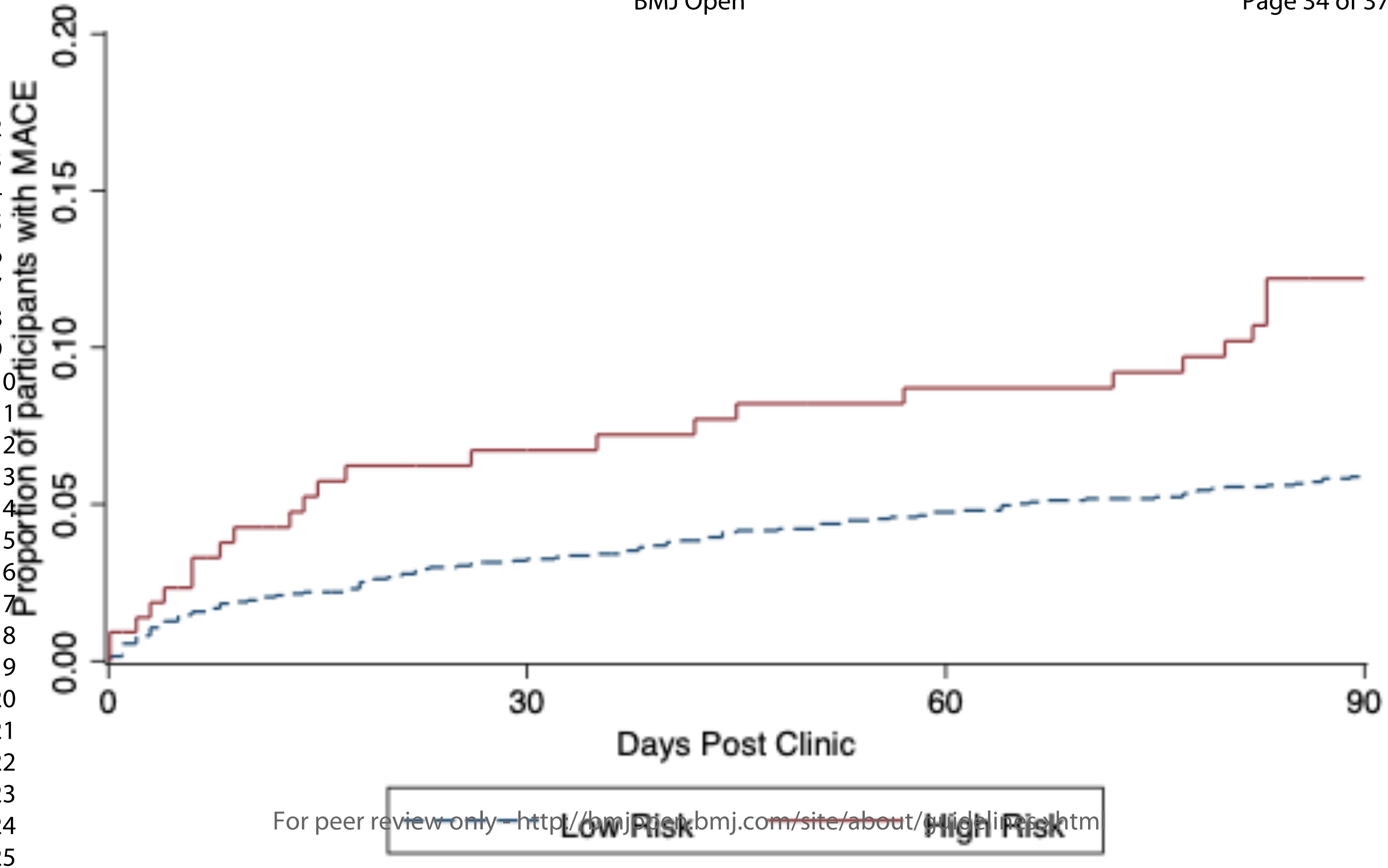


For peer review only - http://bmjopen.bmj.com/site/about/guidelines.xhtml

**Supplementary Table 1: APA (Age, Previous Stroke and ACR) Score derivation**

| | Variable | Value |
|---|---|---|
| **Age** | ≥80 years | 1 |
| **Previous stroke or TIA** | Present | 1 |
| **ACR** | ≥3.5 mg/mmol for women<br>≥2.5 mg/mol for men | 1 |

| | | |
|---|---|---|
| **Score** | 0-1 | 2-3 |
| **Risk** | Low Risk | High Risk |

ACR: Albumin Creatinine Ratio

For peer review only

**Supplementary Table 2**: Occurrence of major adverse cardiac events (MACE) by APA risk score group (p for Chi-Squared)

| APA score | N | Occurrence of MACE events (%) |
|---|---|---|
| **0** | 1215 | 4.9 |
| **1** | 770 | 7.0 |
| **2** | 207 | 11.6 |
| **3** | 10 | 30 |
| | | P<0.001 |

For peer review only

Supplementary Table 3
The predictive values of the elements of the APA score compared to the total score in the study and the validation population.

Table 3a The predictive values in the original study population

| | PPV | NPV | Sensitivity | Specificity |
|---|---|---|---|---|
| Aged >80 years | 7.9% | 94.2% | 32.1% | 74.7% |
| Previous Stroke | 11.9% | 93.9% | 7.1% | 96.4% |
| Age >80 years and Previous Stroke | 8.4% | 94.5% | 37.9% | 72.1% |
| APA score ≥2 | 12.4% | 94.6% | 19.3% | 91.2% |

Table 3b The predictive values in the validation population

| | PPV | NPV | Sensitivity | Specificity |
|---|---|---|---|---|
| Aged >80 years | 9.8% | 91.3% | 38.5% | 64.6% |
| Previous Stroke | 18.2% | 93.6% | 46.2% | 79.2% |
| Age >80 years and Previous Stroke | 13.3% | 91.4% | 15.4% | 90.0% |
| APA score ≥2 | 24.4% | 96.9% | 76.9% | 75.4% |

PPV Positive Predictive Value
NPV Negative Predictive Value
APA Composite of Age >80 years, Previous stroke, elevated ACR. A score of 2 or 3 are regarded as high risk

For peer review only

# TRIPOD Checklist: Prediction Model Development

| Section/Topic | Item | Checklist Item | Page |
|---|---|---|---|
| **Title and abstract** | | | |
| Title | 1 | Identify the study as developing and/or validating a multivariable prediction model, the target population, and the outcome to be predicted. | 1 |
| Abstract | 2 | Provide a summary of objectives, study design, setting, participants, sample size, predictors, outcome, statistical analysis, results, and conclusions. | 3 |
| **Introduction** | | | |
| Background and objectives | 3a | Explain the medical context (including whether diagnostic or prognostic) and rationale for developing or validating the multivariable prediction model, including references to existing models. | 6 |
| | 3b | Specify the objectives, including whether the study describes the development or validation of the model or both. | 7 |
| **Methods** | | | |
| Source of data | 4a | Describe the study design or source of data (e.g., randomized trial, cohort, or registry data), separately for the development and validation data sets, if applicable. | 7 |
| | 4b | Specify the key study dates, including start of accrual; end of accrual; and, if applicable, end of follow-up. | 7 |
| Participants | 5a | Specify key elements of the study setting (e.g., primary care, secondary care, general population) including number and location of centres. | 7 |
| | 5b | Describe eligibility criteria for participants. | 7 |
| | 5c | Give details of treatments received, if relevant. | 7-8 |
| Outcome | 6a | Clearly define the outcome that is predicted by the prediction model, including how and when assessed. | 9 |
| | 6b | Report any actions to blind assessment of the outcome to be predicted. | 9 |
| Predictors | 7a | Clearly define all predictors used in developing or validating the multivariable prediction model, including how and when they were measured. | 9-10 |
| | 7b | Report any actions to blind assessment of predictors for the outcome and other predictors. | 8 |
| Sample size | 8 | Explain how the study size was arrived at. | 9 |
| Missing data | 9 | Describe how missing data were handled (e.g., complete-case analysis, single imputation, multiple imputation) with details of any imputation method. | n/a |
| Statistical analysis methods | 10a | Describe how predictors were handled in the analyses. | 9-10 |
| | 10b | Specify type of model, all model-building procedures (including any predictor selection), and method for internal validation. | 9-10 |
| | 10d | Specify all measures used to assess model performance and, if relevant, to compare multiple models. | 9-10 |
| Risk groups | 11 | Provide details on how risk groups were created, if done. | |
| **Results** | | | |
| Participants | 13a | Describe the flow of participants through the study, including the number of participants with and without the outcome and, if applicable, a summary of the follow-up time. A diagram may be helpful. | 10 |
| | 13b | Describe the characteristics of the participants (basic demographics, clinical features, available predictors), including the number of participants with missing data for predictors and outcome. | 10 & t1 |
| Model development | 14a | Specify the number of participants and outcome events in each analysis. | 11 & t1 |
| | 14b | If done, report the unadjusted association between each candidate predictor and outcome. | n/a |
| Model specification | 15a | Present the full prediction model to allow predictions for individuals (i.e., all regression coefficients, and model intercept or baseline survival at a given time point). | 12 |
| | 15b | Explain how to the use the prediction model. | |
| Model performance | 16 | Report performance measures (with CIs) for the prediction model. | 13 |
| **Discussion** | | | |
| Limitations | 18 | Discuss any limitations of the study (such as nonrepresentative sample, few events per predictor, missing data). | 16 |
| Interpretation | 19b | Give an overall interpretation of the results, considering objectives, limitations, and results from similar studies, and other relevant evidence. | 14-15 |
| Implications | 20 | Discuss the potential clinical use of the model and implications for future research. | 15 |
| **Other information** | | | |
| Supplementary information | 21 | Provide information about the availability of supplementary resources, such as study protocol, Web calculator, and data sets. | ST1 |
| Funding | 22 | Give the source of funding and the role of the funders for the present study. | 19 |

We recommend using the TRIPOD Checklist in conjunction with the TRIPOD Explanation and Elaboration document.

For peer review only

BMJ Open

# The evaluation of microalbuminuria as a prognostic indicator after a TIA or minor stroke in an outpatient setting: The Prognostic Role of Microalbuminuria in TIA Evolution (ProMOTE) Study

| | |
|---|---|
| Journal: | *BMJ Open* |
| Manuscript ID | bmjopen-2020-043253.R3 |
| Article Type: | Original research |
| Date Submitted by the Author: | 22-Jun-2021 |
| Complete List of Authors: | Strain, W; University of Exeter, Diabetes and Vascular Research Centre; Royal Devon and Exeter NHS Foundation Trust, Academic Department of Healthcare for Older People<br>Elyas, Salim; University of Exeter, Diabetes and Vascular Research Centre; Royal Devon and Exeter NHS Foundation Trust, Academic Department of Healthcare for Older People<br>Wedge, Nicola; Royal Devon and Exeter NHS Foundation Trust, Academic Department of Healthcare for Older People<br>Mounce, Luke; University of Exeter, Institute of Health Research<br>Henley, W; University of Exeter Medical School, Institute of Health Research<br>James, Martin; Royal Devon and Exeter NHS Foundation Trust, Academic Department of Healthcare for Older People<br>Shore, Angela; University of Exeter Medical School, NIHR Exeter Clinical Research Facility |
| <b>Primary Subject Heading</b>: | Medical management |
| Secondary Subject Heading: | Diagnostics |
| Keywords: | Stroke medicine < INTERNAL MEDICINE, STROKE MEDICINE, Risk management < HEALTH SERVICES ADMINISTRATION & MANAGEMENT |
| | |

SCHOLARONE™
Manuscripts

*I, the Submitting Author has the right to grant and does grant on behalf of all authors of the Work (as defined in the below author licence), an exclusive licence and/or a non-exclusive licence for contributions from authors who are: i) UK Crown employees; ii) where BMJ has agreed a CC-BY licence shall apply, and/or iii) in accordance with the terms applicable for US Federal Government officers or employees acting as part of their official duties; on a worldwide, perpetual, irrevocable, royalty-free basis to BMJ Publishing Group Ltd ("BMJ") its licensees and where the relevant Journal is co-owned by BMJ to the co-owners of the Journal, to publish the Work in this journal and any other BMJ products and to exploit all rights, as set out in our licence.*

*The Submitting Author accepts and understands that any supply made under these terms is made by BMJ to the Submitting Author unless you are acting as an employee on behalf of your employer or a postgraduate student of an affiliated institution which is paying any applicable article publishing charge ("APC") for Open Access articles. Where the Submitting Author wishes to make the Work available on an Open Access basis (and intends to pay the relevant APC), the terms of reuse of such Open Access shall be governed by a Creative Commons licence – details of these licences and which Creative Commons licence will apply to this Work are set out in our licence referred to above.*

*Other than as permitted in any relevant BMJ Author's Self Archiving Policies, I confirm this Work has not been accepted for publication elsewhere, is not being considered for publication elsewhere and does not duplicate material already published. I confirm all authors consent to publication of this Work and authorise the granting of this licence.*

For peer review only

# The evaluation of microalbuminuria as a prognostic indicator after a TIA or minor stroke in an outpatient setting: The Prognostic Role of Microalbuminuria in TIA Evolution (ProMOTE) Study

Running Title: Predictive role of microalbuminuria after TIA and minor strokes

**W David Strain[1,2,4],**

**Salim Elyas[1,2],**

**Nicola Wedge[2,4],**

**Luke Mounce[3],**

**William Henley[3],**

**Martin A James[2],**

**Angela C Shore[1,4]**

1 Diabetes and Vascular Research Centre, University of Exeter Medical School. Barrack Road, Exeter EX2 5AX

2 Academic Department of Healthcare for Older People, Royal Devon & Exeter Hospital Barrack Road, Exeter EX2 5DW

3 University of Exeter, College of Medicine and Health, Exeter, UK

4 NIHR Exeter Clinical Research Facility

**Address for Correspondence:**

Dr David Strain

Diabetes and Vascular Research Centre

University of Exeter Medical School, Royal Devon & Exeter Hospital

Barrack Road, Exeter. EX2 5AX UK

**Email. d.strain@exeter.ac.uk**

**Tel: +441392 403058**

**Fax: +441392 403027**

For peer review only

| | | |
|---|---|---|
| **Word count** | Manuscript | 3168 |
| | Abstract | 334 |

**Disclosures**

All authors confirm that there are no conflicts of interest

For peer review only


For peer review only - http://bmjopen.bmj.com/site/about/guidelines.xhtml

**Abstract**:

**Objective**: Transient ischaemic attacks (TIA) and minor strokes are important risk factors for further vascular events. We explored the role of albumin-creatinine ratio (ACR) in improving risk prediction after a first event.

**Setting**: Rapid access stroke clinics in the UK

**Participants**. 2,202 patients attending with TIA or minor stroke diagnosed by the attending stroke physician, able to provide a urine sample to evaluate albumin creatinine ratio (ACR) using a near patient testing device.

**Primary and secondary outcomes**. Primary outcome was Major Adverse Cardiac Events (MACE; recurrent stroke, myocardial infarction, or cardiovascular death) at 90 days. The key secondary outcome was to determine whether urinary ACR could contribute to a risk prediction tool for use in a clinic setting.

**Results**: 151 MACE occurred in 144 participants within 90 days. Participants with a MACE had higher ACR than those without. A composite score awarding a point each for Age >80, Previous stroke/TIA and presence of microalbuminuria identified those at low and high risk. 90% of patients were at low risk (scoring 0 or 1). Their 90-day risk of a MACE was 5.7%. Of the remaining "high risk" population (scoring 2 or 3) 12.4% experienced MACE over 90 days ($p<0.001$ compared to low risk population). The need for acute admission in the first 7 days was two-fold elevated in the high-risk group compared to the low risk (3.23 vs. 1.43%; $p=0.05$). These findings were validated in an independent historic sample.

**Conclusion**: A risk score comprising age, previous stroke/TIA and microalbuminuria predicts future MACE events whilst identifying those at low risk of a recurrent event. This tool shows promise in the risk stratification of patients to avoid the admission of low-risk patients.



For peer review only - http://bmjopen.bmj.com/site/about/guidelines.xhtml

**Keywords**:

Stroke,

TIA,

Microalbuminuria,

ACR,

Risk prediction.

For peer review only

## Article Summary

*Strengths and limitations of this study*

- The pragmatic design of this study, provides good generalisability to clinical practice
- The predictive role of the combination of urinary ACR in combination with very basic demographics would be able to reassure the 90% of the population that there was only approximately a 1 in 20 risk of an event recurring, whereas the higher risk population had a 1 in 8 chance of a MACE outcome
- The study is limited by the availability of data to that which was available to the physicians at the time of the stroke appointment. This limits the ability to determine mechanistic association between ACR and MACE outcome
- Further work is required to determine whether therapies that reduce ACR can modify the risk of subsequent MACE

For peer review only

**Introduction:**

Stroke is the second most common cause of death and a leading cause of disability worldwide[1]. Despite, or possibly because of, recent trends in reducing stroke mortality, the health and social disability burden of stroke is increasing[2]. After advancing age, transient ischaemic attacks (TIA) and minor strokes are the most important risk factors for recurrent stroke and predict long term mortality.[3,4] 23% of patients presenting with stroke have a history of TIA in the 3 months prior to the index event.[5] This is a key population to target for secondary prevention, but these patients represent less than 10% of all those who present to rapid access TIA clinics.[6,7] Notably, half of all completed strokes occur within the first week after TIA or minor stroke.[5]

Accurate identification of those patients presenting to TIA clinics with TIA or minor stroke who are most at risk for future events is important in order to i) intensify treatments such as giving dual anti-platelet therapy[8,9] ii) guide the need for urgent admission to facilitate the detection and urgent surgical correction of severe internal carotid artery (ICA) stenosis[10] iii) reassure patients with lowest risk.

Several risk stratification tools are already utilised in such clinics such as ABCD2[11,12] (awarding points for Age, presenting Blood pressure, Clinical features of unilateral weakness or aphasia, Duration of symptoms and Diabetes), California score[6] and imaging based scoring systems.[10,13] These tools lack optimal sensitivity and specificity.[12,14] Indeed some studies suggest patients with a 'low risk' ABCD2 score (less than 4/7) have similar 90 day stroke risk as patients deemed high risk with an ABCD2 score above 4/7,[15] whilst missing up to 40% of patients with severe ICA stenosis.[16] As such, recent consensus guidelines have advised against their use.[17-19]

For peer review only

Increased urinary albumin excretion rate (AER) has been shown to predict incident stroke and heart failure in people with diabetes. In the general population, AER predicts cardiovascular disease and mortality post-stroke independent of conventional cardiovascular risk factors such as hypertension, diabetes and smoking [20-23]. Urinary albumin creatinine ratio (ACR) is a well recognised proxy for urinary AER, and can now be assessed using simple and inexpensive point of care equipment. In an earlier pilot study of 142 patients with minor stroke/TIA,[24] we identified the potential role of urinary ACR in identifying those at highest risk of a recurrent event. Although statistically significant, this study was not large enough to explore potential confounding. We therefore performed a definitive study to assess whether urinary ACR improved the risk stratification of patients presenting with TIA or minor stroke to UK acute stroke clinics.

**Methods:**

Patients diagnosed with suspected minor stroke/TIA by a consultant stroke physician, attending a rapid access clinics in one of 12 UK hospitals were recruited at the end of their clinic consultation. As these clinics are rapid access, often within the first 24 hours of symptoms, it was impossible to accurately differentiate between TIA (with symptoms less than 24 hours) and minor stroke in all cases. Only individuals with events that had occurred within the previous month were included in the study. Routine clinical care, including urgent revascularisation in those with severe ICA stenosis (> 50% diameter stenosis by the North American Symptomatic Carotid Endarterectomy Trial (NASCET) method,[25] was initiated prior to enrollment. If a cardioembolic source was identified, anticoagulants were initiated according to local protocols. After written,



For peer review only - http://bmjopen.bmj.com/site/about/guidelines.xhtml

informed consent was obtained in the clinic, demographics, including age, sex, height, weight, past medical history and ABCD2 score were recorded. Times from onset of symptoms to assessment and enrollment in the study were documented. A clean specimen of urine was collected from participants in the clinic and tested with a Unistix dipstick and, if there was no indication of urinary tract infection (presence of any two of leucocytes, nitrites and/or protein), ACR was measured using a point of care analyzer (The Afinion™ AS100 Analyzer, Axis Shield, Dundee, UK). This system uses an immunometric membrane flow-through principle for albumin measurement and an enzymatic colorimetric test for creatinine quantification[26] and reports an ACR in approximately 5 minutes within the range 0.1 to 140 mg/mmol with a coefficient of variation of 4.6-6%.[26] Participants with values of less than 0.1 mg/mmol were recorded as having the lowest recordable value of ACR (0.1 mg/mmol).

Participants were followed up by telephone on day 7, 30 and 90, and any history of any further vascular events, hospitalisation or death was obtained. If contact with participants was not possible initially, the presence or absence of further events was verified during additional attempts at contact or during future follow up. If no further contact was possible with the participant (e.g. in the case of significant stroke or death), medical history was collected from the next of kin and verified from the primary care physician and hospital records. All reported clinical events were adjudicated according to standardised diagnostic criteria by a data monitoring committee including two independent stroke physicians, blinded to the ACR, by examination of clinical records if they attended hospital, or their primary care physician, or by verifying their clinical symptoms from the research records if patients did not seek further medical advice. In the event of disagreement, a third independent stroke physician acted as adjudicator.



For peer review only - http://bmjopen.bmj.com/site/about/guidelines.xhtml

The primary outcome was to determine the utility of microalbuminuria in predicting the time to first major adverse cardiovascular event (MACE; recurrent stroke, myocardial infarction or death) within 90 days. Secondary outcomes were time to explore the predictive role of microalbuminuria on recurrent stroke, total mortality (even if these were not the first MACE event), the presence of first MACE within 7 days, and the need for hospitalisation within the first 7 days. All events were adjudicated blind to urinary albumin creatinine ratio by an independent data monitoring committee.

*Ethics Statement*

The study protocol was approved by the national research ethics committee (approval 14/EE/1106). All participants provided written informed consent prior to enrolment and confirmed their willingness to continue participation at each telephone consultation.

*Patient and public involvement*

Patients were involved in the design and conduct of this research. During the feasibility stage, priority of the research question, and methods of recruitment were informed by discussions with patients through a focus group session and three structured interviews. During the trial, two patients joined the independent trial steering committee. Once the trial has been published, participants will be informed of the results through a dedicated newsletter suitable for a non-specialist audience, led by one of the patients on the steering committee.

*Statistical analysis:*



For peer review only - http://bmjopen.bmj.com/site/about/guidelines.xhtml

Data were treated as continuous variables wherever possible to maximize power. All normally distributed data are presented as mean ± standard deviation (SD). Skewed data were appropriately transformed and presented as geometric means (with 95% confidence intervals [CI]). Statistical significance for categorical variables was calculated using the chi-squared test and the Student's t-test for continuous variables. Time to event was measured from the clinic consultation rather than the index event, in keeping with the pragmatic nature of the study. Independence of ACR (as a risk predictor) from diabetes and other measures of the ABCD2 score was assessed using logistic regression. Microalbuminuria was defined using the currently accepted clinical thresholds for microalbuminuria used in people with diabetes 3.5mg/mol for women and 2.5mg/mol for men. Identification of independent predictors of recurrent MACE events was performed by backwards stepwise logistic regression, commencing with all information available for the complete dataset, representing all information that would routinely be available to the clinician in the clinic setting. Where multiple related measures were available (e.g. systolic, diastolic and mean arterial blood pressure) the variable that accounted for the greatest degree of variance in a univariate analysis was included. Fractional polynomial regression modeling was used to identify sex-specific thresholds of ACR to define "microalbuminuria" in the context of stroke risk stratification. Prediction of events were calculated using a Cox proportional hazard ratio with time to first MACE as the primary outcome. In keeping with the recommendations of Cupples[27] and Rothman[28] the measured significance of the variables of interest is reported without adjustment for multiple testing. Statistical significance was considered at $p<0.05$. Statistical analysis was performed using Stata SE 14.2 (Mac version: Statacorp ltd Texas).



For peer review only - http://bmjopen.bmj.com/site/about/guidelines.xhtml

**Results:**

Two thousand, four hundred and eight patients with a diagnosis of definite or probable minor stroke/TIA were recruited; 149 participants were subsequently excluded after the diagnosis was revised to that of a stroke mimic, 8 withdrew consent and a further 49 (2%) were excluded due to intercurrent urinary tract infection rendering the assessment of ACR invalid. No patient was lost to follow-up. Baseline characteristics are presented in table 1. Of the 2,202 included in the final analysis, most were male (64.9%). All patients were commenced on secondary prevention with appropriate antiplatelet or anticoagulant therapy, and an appropriately dosed statin in accordance with contemporaneous national guidelines.

Over 90 days, 151 primary outcome events (MACE) occurred in 144 participants (6.7% of patients) including 8 cardiovascular deaths. All MACE events were atherosclerotics with no haemorrhagic events occuring in the 3 months. There were also 8 non-cardiovascular deaths. Within 7 days there were 38 MACE in 36 participants. Those with MACE were more likely to have had previous stroke/TIA, however, with this exception, there were no significant differences in routinely collected clinic data (Table 1). There was no difference in mean ABCD2 score or the proportion of participants with a 'high risk' score (4/7 or more) between those who did and did not have events by 90 days. When looking exclusively at the 1374 participants identified with a "high risk" ABCD2 score (63% of the study sample; 93% male), a high score did not distinguish those at high risk of MACE event compared to those with an ABCD2 <4 (HR 1.15 (0.82-1.63); p=0.4), nor did a low risk score exclude an event, with only 37.7% of people who did not have experience a MACE outcome scoring below 4. Point-of-care ACR was higher in those participants with a primary outcome event at 90 days



For peer review only - http://bmjopen.bmj.com/site/about/guidelines.xhtml

compared to those without (2.27 (1.92-2.68) vs. 1.75 (1.68–1.83) mg/mmol respectively; p=0.002). This distinction remained after adjustment for age and sex (adjusted ACR 2.40 (2.04-2.83) vs. 1.88 (1.80 – 1.96), p=0.004. Figure 1).

*Evaluation of the prognostic role of microalbuminuria alone*

Fractional polynomial regression modelling identified the sex-specific threshold of risk for ACR aligned with the currently accepted thresholds for microalbuminuria used in people with diabetes of 2.5mg/mmol for men and 3.5mg/mol for women. These thresholds identified 562 participants (25.4% of the study sample; 75% male) with microalbuminuria. These participants were more likely than those with a low ACR to experience a primary outcome event (Hazard Ratio 1.66 (95% CI 1.19 - 2.34); p=0.003) and had a greater than 5-fold increase in 90-day mortality (HR 5.44 (1.86 – 15.90); p=0.0003). Alone, however, microalbuminuria did not have sufficient positive or negative predictive value to justify its clinical utility (Positive predictive value – 9.1%, Specificity 75.4%).

*Generating a composite risk score for 90-day risk stratification*

In a stepwise multivariate model, the only independent predictors of MACE were elevated ACR, age greater than 80 and history of previous stroke or TIA. Similar variance within the model was explained microalbuminuria (i.e. an ACR >2.5 and 3.5mg/mol respectively in men and women) as being older than 80 years and previous history of stroke/TIA, so they were afforded equal weighting. This generated a 4-point scale between 0 and 3 (termed the 'APA' score representing Age, Prior stroke or TIA and elevated ACR), with the lowest risk participants having a score of 0 (Supplementary Table 1). There was a sequential increase in risk with increasing score



For peer review only - http://bmjopen.bmj.com/site/about/guidelines.xhtml

such that those with a score of 0 having a 90-day risk of 4.86% rising to a 30% risk for those with the highest score (supplementary table 2). The population was then divided into a low risk group scoring 0 or 1 or high risk scoring 2 or 3.  1,985 (90.1%) of participants were identified as low risk compared to 217 (9.9%) in the high-risk group. When comparing the positive predictive value (PPV) sensitivity and specificity of the APA score compared to the previously available parameters of age and previous stroke demonstrated a clinically meaningful improvement over each measure on its own (Supplementary table 3a). Compared to using previous stroke alone, APA score had a similar positive predictive value (12.4% for APA score vs 11.9% for previous stroke) and specificity (91.2% vs 96.4%, respectively), however superior sensitivity (19.3% vs 7.1%, respectively). Sensitivity was improved by using a composite of previous stroke and being over the age of 80 years, however this was at the expense of a reduction of positive predictive value to 8.4%.

The high-risk group was older, with higher systolic blood pressure and pulse pressure compared to the low-risk group (table 2).  The 90-day risk of a MACE outcome was 5.7% in the low-risk group, versus 12.4% in the high-risk group ($p<0.001$), translating to a hazard ratio of 2.12 (95% CI 1.38 – 3.25) for the high-risk score compared to low-risk group ($p<0.001$; Figure 2). This was predominantly driven by an increase in recurrent stroke/TIA (3.3% vs. 8.8%; HR 2.67(1.58-4.51); $p<0.001$). This difference was apparent within 7 days of the clinic, such that those with a high APA score had a 2.2-fold increased risk of a recurrent event.

One thousand and forty-nine participants were reviewed within 48 hours of their index event. For these individuals, the predictive role of the APA score was numerically


For peer review only - http://bmjopen.bmj.com/site/about/guidelines.xhtml

superior (HR 2.84, 95% CI 1.32-6.14; p=0.008) than those who were seen with a longer delay. There was no significant interaction between time from event to appointment and predictive role of the APA score.

*Validation of APA score on a historic sample*

In order to validate the APA score, we applied the tool to the individuals who participated in the pilot study but did not contribute their data to this dataset. Details of this pilot study have been published elsewhere [24]. In brief, the pilot study recruited 139 participants over 9 months from one of the centres involved in the definitive study. In this population, there were 13 recurrent events (9.35%) within the 90 days, 9 of which occurred in the first 7 days after attendance to the stroke clinic (Table 3). The APA risk score replicated a similar distribution of number of participants at low risk (73%) and high risk (27%) as in the larger study. Compared to the component parts, the APA score had superior positive predictive value (24.4% vs 18.2% for Previous Stroke or 13.3% for previous stroke and being aged >80 years) and superior sensitivity (76.9% vs 46.2% vs 15.4% respectively: supplementary table 3b). At 90 days, 5.9% of the low APA group had recurrent events compared to 18.4% of the in the high-APA group (p=0.02). Again, within the first 7 days this was apparent with only 1.9% of the low APA group experiencing the primary outcome, compared to 13.2% in the high APA group (p=0.04).

**Discussion:**

In this large UK multicentre prospective study with blinded outcome adjudication, we have demonstrated the utility of an elevated ACR, measured using a simple point-of-care analyser, in combination with simple clinical data (age and previous history of stroke or TIA) in the prediction of major adverse cardiovascular events and death. This


For peer review only - http://bmjopen.bmj.com/site/about/guidelines.xhtml

differentiates between low and high-risk individuals. Specifically, over 90% of individuals were identified as low risk with less than a 1.5% risk of a major adverse cardiovascular event or death over the next 7 days, increasing to 5.7% by 90 days, whereas 9.9 % of the population were identified at high risk with a 12.4% event rate over 90 days. Although only a small number, our findings were validated in a separate historic population who participated in the pilot study.

The utility of APA scoring may go beyond simply guiding conversations regarding prognosis in a clinic setting. The POINT[8] and CHANCE[9] studies demonstrated the benefit of dual anti-platelet therapy in the first 21 days after high risk TIA. There was, however, a 0.5% absolute increase in major intracranial haemorrhage. We would propose that an assessment of the APA score could be evaluated as a stratification tool to determine if it predicts those who benefit the most. In those with a low-risk APA score, even the 25% relative risk reduction in the POINT trial would result in only a marginally beneficial risk:benefit ratio, assuming that microalbuminuria is a predictor of thromboembolic disease processes. Those with a high APA score, however, would likely benefit from dual-antiplatelet therapy with a number need to treat of less than 30 in order to prevent an event. This suggestion, however, does require confirmation in an independent study. The APA score also gives additional information that is of use in the more acute setting. Over the first 7 days, a low-risk APA score had a negative predictive value of >98.5% for the risk of a MACE - a number very similar to the predictive effect of troponin when evaluating suspected cardiac chest pain.[29]

When considering the populations identified by the APA score, there are similarities to the ABCD2 score. Indeed, the demographics of the high-risk group were older, with



For peer review only - http://bmjopen.bmj.com/site/about/guidelines.xhtml

higher blood pressure and a trend towards more diabetes. We would suggest, that microalbuminuria represents vascular susceptibility to the adverse consequences of hypertension and diabetes rather than simply an indication of the presence of hypertension and /or diabetes in general. This would explain why the APA score predicted further MACE and total mortality at 7 and 90 days, whereas in our large sample the ABCD2 score did not.

Elevated ACR is a recognised marker of generalised endothelial and microvascular dysfunction. The increased filtration of albumin through the renal glomerular filtration barrier is thought to be due to changes in the chemical and physical properties of this endothelial barrier and its glycocalyx.[30-32] The mechanism explaining the association between microalbuminuria and incident cardiovascular events is thought to depend on its role as a marker of systemically increased vascular permeability and altered homeostasis, coagulation and endothelial function.[33-38] However, after acute ischaemic events such as myocardial infarction, or as demonstrated here TIA or minor stroke, it is likely that elevated urinary ACR is, at least in part, dependent on the systemic inflammatory response to the original insult.

Whilst unlikely to be mechanistically involved in the progression of disease, the question as to whether it is a good surrogate of therapeutic effect remains. Improving systemic inflammation, such as through the pleiotropic effect of statins, has been associated with simultaneous improvements in urinary albumin excretion and CV event rates in patients with elevated high sensitivity C-reactive protein (hsCRP).[39-41] Further studies are needed to determine whether microalbuminuria after acute stroke represents

For peer review only


For peer review only - http://bmjopen.bmj.com/site/about/guidelines.xhtml

a therapeutic target amenable to treatment, and if reductions in microalbuminuria are associated with reductions in the vascular events they predict.

*Limitations and strengths of study*

Recurrent event rate was lower than anticipated from our pilot study and contemporaneous clinical trials such as CHANCE (11.7% in the control arm),[9] although are comparable with the recently-reported randomised controlled POINT trial (6%).[8] In usual practice it is usually 24-48 hours between the index event and clinic attendance. Given that data from previous studies has demonstrated the highest risk occurs in the first 48 hours, it is likely that some events will have occurred between the index event and attendance in the clinic in our study.[42] The pragmatic design of the study, however, is also a strength given that the predictive role highlights those that may benefit from dual anti-platelet therapy or hospitalisation when the patient is seen in the acute stroke clinic.

There is no consensus on the gold standard for diagnosing TIA in a rapid access stroke clinic.[43] In order to maintain the generalisability of the study, all attendees of the stroke clinic that were diagnosed with a probable or definite TIA were invited to participate. Only a small number of patients were subsequently withdrawn as being stroke mimics. Outcome events were rigorously adjudicated against published diagnostic criteria to ensure consistency and reliability.

Finally, the use of a single urinary ACR sample obtained during the clinic assessment using a near patient testing kit is not as robust as a sample tested in a central laboratory. Whilst there is significant diurnal variability in urinary AER within individuals,



For peer review only - http://bmjopen.bmj.com/site/about/guidelines.xhtml

potentially limiting the scientific or mechanistic merit of the study compared to studies using overnight or 24-hour urine collection, the pragmatic design makes this study more applicable to general clinic populations. Further, the relatively low cost of the near patient test kit makes it accessible in most emergency settings, in primary and secondary care.

**Conclusion:**

We have demonstrated for the first time the potential utility of a single point of care test of urinary ACR in patients presenting with TIA and minor stroke as a prediction tool to assist triaging patients. When used in combination with patient age and previous history of stroke or TIA, this generated a risk stratification score (the APA score: Age>80, Past history of stroke/TIA, and elevated ACR) that could reliably identify the large proportion of patients with a low risk of recurrent vascular event over the next 90 days. The value of ACR that is most predictive is aligned with the conventional definition of microalbuminuria used in diabetes, suggesting that in clinical practice the existing technology for evaluating microalbuminuria including urine dipsticks may be used. A high APA score was associated with a doubling of risk of MACE outcome and a five fold increase in mortality. This APA score was validated in an independent population. We believe the APA score may represent a practical tool for clinicians engaged in the acute ambulatory assessment of TIA and minor stroke, to assist in the assessment of risk:benefit ratio for the use dual anti-platelet therapy or to admit for more urgent investigation. Further work is required to determine whether the increase in ACR represents a therapeutic target or solely a prognostic indicator.

For peer review only



For peer review only - http://bmjopen.bmj.com/site/about/guidelines.xhtml

**Contributorship statement**

WDS & ACS conceived and designed the study, attained funding, were involved in the management of the trial. WDS produced the first draft of the manuscript. SE, NW & MAJ contributed to the design and management of the trial. LM &WH provided the statistical support for the management of the trial and the analysis of the outcomes. All authors have reviewed the final manuscript prior to submission.

**Competing interests**

No authors report any competing interests relevant to this manuscript.

**Funding**

This study was funded by a NIHR Research for Patient Benefit grant (PB-PG-1112-29069) and supported by the NIHR Exeter Clinical Research Facility.

**Data sharing statement**

Anonymised data is available from the corresponding author on reasonable request.

**Acknowledgements**

WDS is supported by the NIHR Exeter Clinical Research Facility. WDS and MAJ is supported by the NIHR SW Peninsula Applied Research Collaboration. This manuscript does not necessarily reflect the views of the NIHR, the Exeter Clinical Research Facility, the NHS or the UK Department of Health.

For peer review only

**References:**


1. Feigin VL, Norrving B, Mensah GA. Global Burden of Stroke. *Circ Res.* 2017;120(3):439-448.
2. Henssge U. National Sentinel Stroke Clinical Audit 2010. In: Royal College of Physicians, ed2011.
3. Burn J, Dennis M, Bamford J, Sandercock P, Wade D, Warlow C. Long-term risk of recurrent stroke after a first-ever stroke. The Oxfordshire Community Stroke Project. *Stroke; a journal of cerebral circulation.* 1994;25(2):333-337.
4. Hankey GJ. Long-term outcome after ischaemic stroke/transient ischaemic attack. *Cerebrovascular diseases (Basel, Switzerland).* 2003;16 Suppl 1:14-19.
5. Rothwell PM, Warlow CP. Timing of TIAs preceding stroke: time window for prevention is very short. *Neurology.* 2005;64(5):817-820.
6. Johnston SC, Gress DR, Browner WS, Sidney S. Short-term prognosis after emergency department diagnosis of TIA. *Jama.* 2000;284(22):2901-2906.
7. Hill MD, Yiannakoulias N, Jeerakathil T, Tu JV, Svenson LW, Schopflocher DP. The high risk of stroke immediately after transient ischemic attack: a population-based study. *Neurology.* 2004;62(11):2015-2020.
8. Johnston SC, Easton JD, Farrant M, et al. Clopidogrel and Aspirin in Acute Ischemic Stroke and High-Risk TIA. *N Engl J Med.* 2018;379(3):215-225.
9. Wang Y, Wang Y, Zhao X, et al. Clopidogrel with aspirin in acute minor stroke or transient ischemic attack. *N Engl J Med.* 2013;369(1):11-19.
10. Kiyohara T, Kamouchi M, Kumai Y, et al. ABCD3 and ABCD3-I scores are superior to ABCD2 score in the prediction of short- and long-term risks of stroke after transient ischemic attack. *Stroke; a journal of cerebral circulation.* 2014;45(2):418-425.


For peer review only



For peer review only - http://bmjopen.bmj.com/site/about/guidelines.xhtml

11. Rothwell PM, Giles MF, Flossmann E, et al. A simple score (ABCD) to identify individuals at high early risk of stroke after transient ischaemic attack. *Lancet.* 2005;366(9479):29-36.

12. Bhatt A, Jani V. The ABCD and ABCD2 Scores and the Risk of Stroke following a TIA: A Narrative Review. *ISRN Neurol.* 2011;2011:518621.

13. Ay H, Arsava EM, Johnston SC, et al. Clinical- and imaging-based prediction of stroke risk after transient ischemic attack: the CIP model. *Stroke; a journal of cerebral circulation.* 2009;40(1):181-186.

14. Giles MF, Rothwell PM. Systematic review and pooled analysis of published and unpublished validations of the ABCD and ABCD2 transient ischemic attack risk scores. *Stroke; a journal of cerebral circulation.* 2010;41(4):667-673.

15. Amarenco P, Labreuche J, Lavallée PC. Patients With Transient Ischemic Attack With ABCD2 <4 Can Have Similar 90-Day Stroke Risk as Patients With Transient Ischemic Attack With ABCD2 ≥4. *Stroke; a journal of cerebral circulation.* 2012;43(3):863-865.

16. Amarenco P, Labreuche J, Lavallee PC. Patients with transient ischemic attack with ABCD2 <4 can have similar 90-day stroke risk as patients with transient ischemic attack with ABCD2 >/=4. *Stroke; a journal of cerebral circulation.* 2012;43(3):863-865.

17. National Institute of Health and Care Excellence. Stroke and transient ischaemic attack in over 16s: diagnosis and initial management; NICE guideline [NG128]. National Institute of Health and Care Excellence. Published 2019. Updated May 2019. Accessed 10/11/20, 2019.

18. Powers WJ, Rabinstein AA, Ackerson T, et al. 2018 Guidelines for the Early Management of Patients With Acute Ischemic Stroke: A Guideline for



For peer review only - http://bmjopen.bmj.com/site/about/guidelines.xhtml

Healthcare Professionals From the American Heart Association/American Stroke Association. *Stroke.* 2018;49(3):e46-e110.

19. Ahmed N, Audebert H, Turc G, et al. Consensus statements and recommendations from the ESO-Karolinska Stroke Update Conference, Stockholm 11–13 November 2018. *European Stroke Journal.*0(0):2396987319863606.

20. Gerstein HC, Mann JF, Yi Q, et al. Albuminuria and risk of cardiovascular events, death, and heart failure in diabetic and nondiabetic individuals. *JAMA.* 2001;286(4):421-426.

21. Hillege HL, Fidler V, Diercks GF, et al. Urinary albumin excretion predicts cardiovascular and noncardiovascular mortality in general population. *Circulation.* 2002;106(14):1777-1782.

22. Yuyun MF, Khaw KT, Luben R, et al. Microalbuminuria, cardiovascular risk factors and cardiovascular morbidity in a British population: the EPIC-Norfolk population-based study. *Eur J Cardiovasc Prev Rehabil.* 2004;11(3):207-213.

23. Strain WD, Shore AC, Melzer D. Albumin:creatinine ratio predicts mortality after stroke: analysis of the Third National Health and Nutrition Examination Survey. *J Am Geriatr Soc.* 2010;58(12):2434-2435.

24. Elyas S, Shore AC, Kingwell H, et al. Microalbuminuria could improve risk stratification in patients with TIA and minor stroke. *Ann Clin Transl Neurol.* 2016;3(9):678-683.

25. Morgenstern LB, Fox AJ, Sharpe BL, Eliasziw M, Barnett HJ, Grotta JC. The risks and benefits of carotid endarterectomy in patients with near occlusion of the carotid artery. North American Symptomatic Carotid Endarterectomy Trial (NASCET) Group. *Neurology.* 1997;48(4):911-915.



For peer review only - http://bmjopen.bmj.com/site/about/guidelines.xhtml

26. Kvam C. Afinion ACR Scientific Poster AACC 2007. Axis-Shield PoC AS. http://www.afinion.net/publications. Published 2007. Accessed.

27. Cupples LA, Heeren T, Schatzkin A, Colton T. Multiple testing of hypotheses in comparing two groups. *Ann Intern Med.* 1984;100(1):122-129.

28. Rothman KJ. No adjustments are needed for multiple comparisons. *Epidemiology.* 1990;1(1):43-46.

29. Gualandro DM, Puelacher C, LuratiBuse G, et al. Comparison of high-sensitivity cardiac troponin I and T for the prediction of cardiac complications after non-cardiac surgery. *Am Heart J.* 2018;203:67-73.

30. Haraldsson B, Jeansson M. Glomerular filtration barrier. *Curr Opin Nephrol Hypertens.* 2009;18(4):331-335.

31. Singh A, Satchell SC. Microalbuminuria: causes and implications. *Pediatr Nephrol.* 2011;26(11):1957-1965.

32. Gansevoort RT, Lambers H, Witte EC. Methodology of screening for albuminuria. *Nephrology Dialysis Transplantation.* 2007;22(8):2109-2111.

33. Barzilay JI, Peterson D, Cushman M, et al. The relationship of cardiovascular risk factors to microalbuminuria in older adults with or without diabetes mellitus or hypertension: the cardiovascular health study. *Am J Kidney Dis.* 2004;44(1):25-34.

34. Valmadrid CT, Klein R, Moss SE, Klein BE. The risk of cardiovascular disease mortality associated with microalbuminuria and gross proteinuria in persons with older-onset diabetes mellitus. *Arch Intern Med.* 2000;160(8):1093-1100.

35. Dinneen SF, Gerstein HC. The association of microalbuminuria and mortality in non-insulin-dependent diabetes mellitus. A systematic overview of the literature. *Arch Intern Med.* 1997;157(13):1413-1418.



For peer review only - http://bmjopen.bmj.com/site/about/guidelines.xhtml

36. Festa A, D'Agostino R, Howard G, Mykkanen L, Tracy RP, Haffner SM. Inflammation and microalbuminuria in nondiabetic and type 2 diabetic subjects: The Insulin Resistance Atherosclerosis Study. *Kidney Int.* 2000;58(4):1703-1710.

37. Asselbergs FW, de Boer RA, Diercks GF, et al. Vascular endothelial growth factor: the link between cardiovascular risk factors and microalbuminuria? *Int J Cardiol.* 2004;93(2-3):211-215.

38. Stehouwer CD, Gall MA, Twisk JW, Knudsen E, Emeis JJ, Parving HH. Increased urinary albumin excretion, endothelial dysfunction, and chronic low-grade inflammation in type 2 diabetes: progressive, interrelated, and independently associated with risk of death. *Diabetes.* 2002;51(4):1157-1165.

39. Ridker PM, Danielson E, Fonseca FA, et al. Rosuvastatin to prevent vascular events in men and women with elevated C-reactive protein. *N Engl J Med.* 2008;359(21):2195-2207.

40. Abe M, Maruyama N, Okada K, Matsumoto S, Matsumoto K, Soma M. Effects of lipid-lowering therapy with rosuvastatin on kidney function and oxidative stress in patients with diabetic nephropathy. *J Atheroscler Thromb.* 2011;18(11):1018-1028.

41. Girardi JM, Farias RE, Ferreira AP, Raposo NR. Rosuvastatin prevents proteinuria and renal inflammation in nitric oxide-deficient rats. *Clinics (Sao Paulo).* 2011;66(8):1457-1462.

42. Coull AJ, Rothwell PM. Underestimation of the early risk of recurrent stroke: evidence of the need for a standard definition. *Stroke; a journal of cerebral circulation.* 2004;35(8):1925-1929.



For peer review only - http://bmjopen.bmj.com/site/about/guidelines.xhtml

43. Graham C, Bailey D, Hart S, et al. Clinical diagnosis of TIA or minor stroke and prognosis in patients with neurological symptoms: A rapid access clinic cohort. *PLoS One*. 2019;14(3):e0210452.

For peer review only

**Table Legends (ProMOTE Study):**

**Table 1**: Sample characteristics of total population and population stratified by occurrence of recurrent major adverse cardiovascular events (MACE) at 90 days Figures presented as mean (SD) for continuous variable except ACR where geometric mean (Inter-quartile range) presented; % for categorical data

P for continuous data by Student's t-test

P for categorical data by Chi Squared test

BMI = Body Mass Index,

ABCD2 (A=Age, B=BP, C=Clinical features, D= Duration of symptoms + Diabetes),

ACR Albumin Excretion Rate

*Note*: Total Cholesterol was collected from routine clinical practice records therefore is not available for all participants

**Table 2**: Occurrence of events by APA risk score group (mean ± SD for continuous variable, p for ANOVA; % for categorical data, p for Chi-Squared)

**Table 3:** Occurrence of events by APA risk score group in independent validation population (mean ± SD for continuous variable, p for ANOVA; % for categorical data, p for Chi-Squared)

For peer review only

Table 1: Sample characteristics of total population and population stratified by occurrence of recurrent vascular events or death (MACE) at 90 days.

| Characteristic | Total population | No event by 90 days | Event at 90 days |
|---|---|---|---|
| N | 2202 | 2058 | 144 |
| Days from event to clinic† | 3 (1-6) | 3 (1-6) | 2 (1-4) |
| Age (years) | 70.9 (11.7) | 70.9 (11.6) | 72.2 (12.2) |
| Sex (%male) | 64.8 | 64.7 | 67.9 |
| Height (m) | 1.70 (0.10) | 1.70 (0.10) | 1.71 (0.9) |
| Weight (kg) | 79.8 (17.1) | 79.9 (17.0) | 79.7 (17.7) |
| BMI (kg/m$^2$) | 27.6 (5.2) | 27.6 (5.2) | 27.2 (4.9) |
| Blood pressure | | | |
| Systolic (mmHg) | 144.1 (20.8) | 144.2 (20.9) | 145.7 (21.7) |
| Diastolic (mmHg) | 81.8 (12.6) | 81.7 (12.6) | 83.8 (13.1) |
| Total Cholesterol§ | (n = 1,466) | (n=1,374) | (n=92) |
| (mmol/L) | 5.02 (2.57) 194 | 5.03 (2.63) | 4.75 (1.24) |
| (mg/dl) | 194(99) | 195 (102) | 183 (48) |
| Diabetes (%) | 15.7 | 15.9 | 12.1 |
| Previous Stroke (%) | 4.0 | 3.5 | 10.7* |
| ABCD2 | | | |
| Mean score out of 7 | 4.0 (1.4) | 4.0 (1.4) | 4.1 (1.4) |
| Proportion >4 (%) | 62.5 | 62.3 | 65.0 |
| "High Risk" | | | |
| ACR (mg/mmol)‡ | 1.79(0.9-2.9) | 1.75(1.69-1.83) | 2.27(1.92-2.68)** |
| ACR (mg/g)‡ | 15.8(8.0-25.6) | 15.5 (14.9-16.2) | 20.1 (17.0-23.7)** |

§ These were collected from patient records where clinician had requested the test and therefore only available in a subset of the population
† Median (InterQuartile Range)
‡ Geometric mean (95% CI)
* p<0.0001 compared to those with no events
** p=0.002 compared to those with no events



For peer review only - http://bmjopen.bmj.com/site/about/guidelines.xhtml

Table 2: Occurrence of events by APA risk score group (mean ± SD for continuous variable, p for ANOVA; % for categorical data, p for Chi-Squared)

| | Low Risk | High Risk | p |
|---|---|---|---|
| n | 1,985 | 217 | |
| Sex (% Male) | 64.5 | 68.2 | 0.2 |
| Age (years) | 69.6 (11.3) | 84.0 (5.0) | <0.0001 |
| Blood pressure (mmHg) | | | |
| Systolic | 143 (20.4) | 150.5 (23.3) | <0.0001 |
| Diastolic | 82 (12.3) | 79 (11.6) | 0.0007 |
| Weight (kg) | 80.3 (17.1) | 75.1 (16.2) | <0.0001 |
| Diabetes | 15.3 | 18.9 | 0.2 |
| MACE at 90 days (%) | | | |
| All events | 5.7 | 12.4 | <0.0001 |
| Cerebrovascular event | 3.2 | 8.8 | <0.0001 |
| Cardiovascular Mortality | 0.1 | 1.4 | <0.001 |
| All Cause Mortality | 0.4 | 2.3 | 0.001 |
| MACE at 7 days (%) | 1.46 | 3.23 | 0.05 |

For peer review only



For peer review only - http://bmjopen.bmj.com/site/about/guidelines.xhtml

**Table 3**: Occurrence of events by APA risk score group in independent validation population (mean ± SD for continuous variable, p for ANOVA; % for categorical data, p for Chi-Squared)

| | Low Risk | High Risk | p |
|---|---|---|---|
| n | 101 | 38 | |
| Sex (% Male) | 51.5 | 71.5 | 0.04 |
| Age (years) | 72.6 (10.7) | 81.6 (6.1) | 0.001 |
| Blood pressure (mmHg) | | | |
| Systolic | 142.6 (20.2) | 147.3 (26.6) | 0.3 |
| Diastolic | 77.7 (9.8) | 77.4 (10.8) | 0.9 |
| Weight (kg) | 76.2 (16.4) | 75.0 (15.4) | 0.7 |
| Recurrent event at 90 days (%) | | | |
| MACE event | 5.9 | 18.4 | 0.02 |
| Recurrent event at 7 days (%) | | | |
| MACE events* | 1.9 | 13.2 | 0.04 |

* MACE = Major Adverse Cardiovascular Event – Recurrent stroke or TIA, Myocardial infarction or cardiovascular death.

For peer review only

**Figure 1:** Age and sex adjusted Albumin Creatinine Ratio (Mean +/-95% CI) stratified by the occurrence of a Major Adverse Cardiac Event (MACE) in the first 90 days post initial event

MACE – Major Adverse Cardiovascular event = recurrent cerebrovascular event, myocardial infarction or cardiovascular death

**Figure 2**: Time to first Major Adverse Cardiac Event (MACE) stratified by APA (Age, Previous cerebrovascular event, High ACR) risk score. Event rate in high risk (n=217) 12.44% vs. low risk (n=1985) 5.69%, HR 2.12 (1.38 – 3.25) p<0.001

For peer review only



For peer review only - http://bmjopen.bmj.com/site/about/guidelines.xhtml

For peer review only



For peer review only - http://bmjopen.bmj.com/site/about/guidelines.xhtml

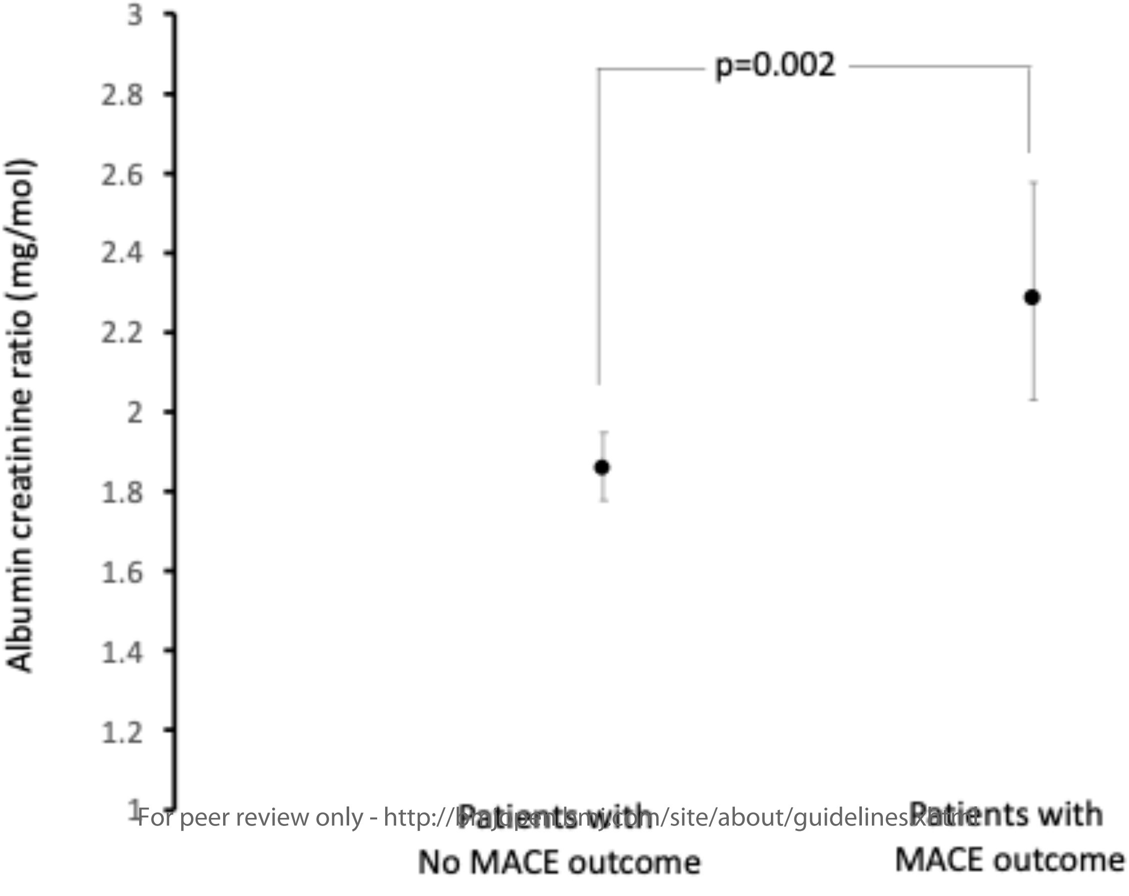

Albumin creatinine ratio (mg/mol)
3
2.8
2.6
2.4
2.2
2
1.8
1.6
1.4
1.2
1
p=0.002
Patients with No MACE outcome
Patients with MACE outcome

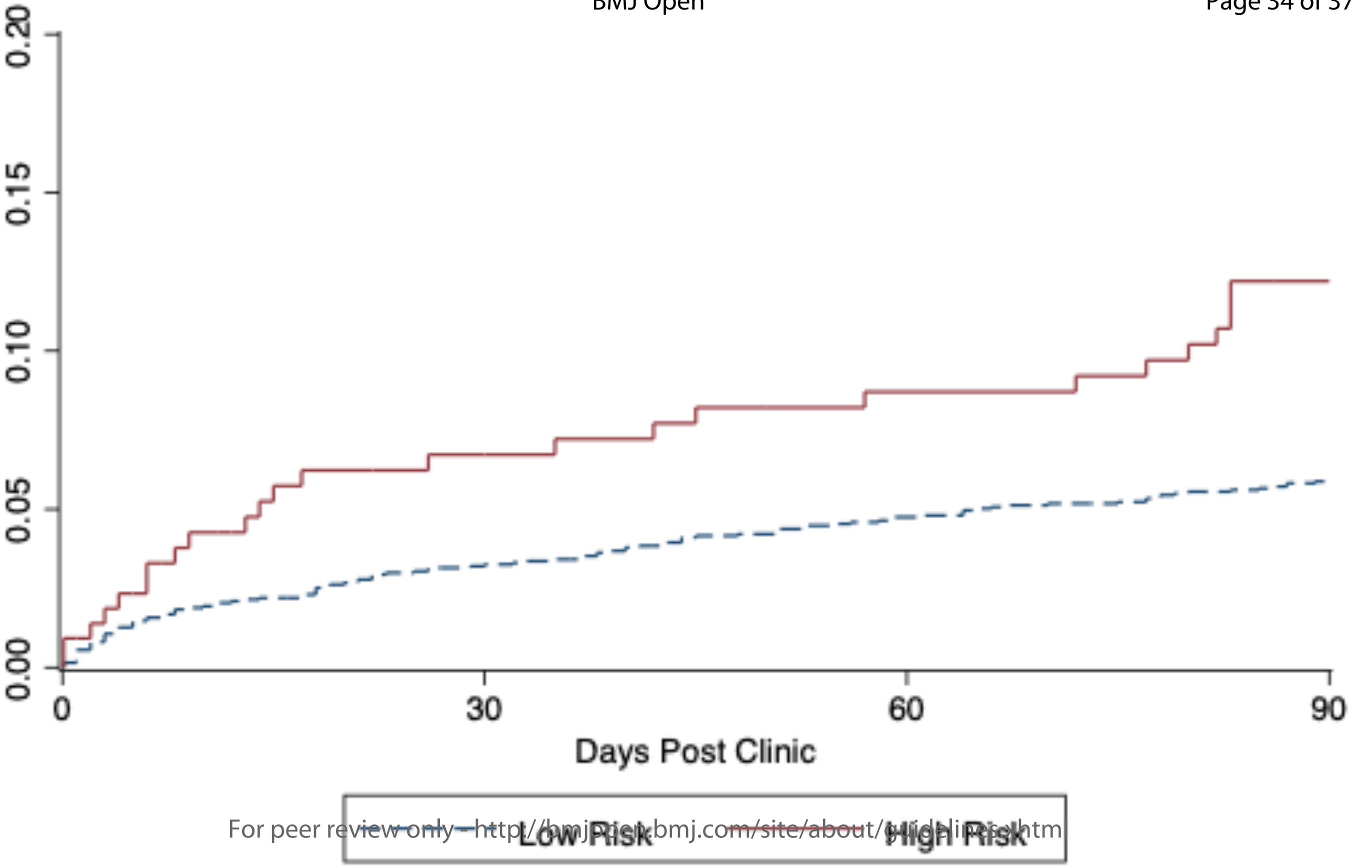


For peer review only - http://bmjopen.bmj.com/site/about/guidelines.xhtml

## Supplementary Table 1: APA (Age, Previous Stroke and ACR) Score derivation

| | Variable | Value |
|---|---|---|
| **Age** | ≥80 years | 1 |
| **Previous stroke or TIA** | Present | 1 |
| **ACR** | ≥3.5 mg/mmol for women<br>≥2.5 mg/mol for men | 1 |

| | | |
|---|---|---|
| **Score** | 0-1 | 2-3 |
| **Risk** | Low Risk | High Risk |

ACR: Albumin Creatinine Ratio

For peer review only

**Supplementary Table 2**: Occurrence of major adverse cardiac events (MACE) by APA risk score group (p for Chi-Squared)

| APA score | N | Occurrence of MACE events (%) |
|---|---|---|
| **0** | 1215 | 4.9 |
| **1** | 770 | 7.0 |
| **2** | 207 | 11.6 |
| **3** | 10 | 30 |
| | | P<0.001 |

For peer review only

Supplementary Table 3
The predictive values of the elements of the APA score compared to the total score in the study and the validation population.

Table 3a The predictive values in the original study population

| | PPV | NPV | Sensitivity | Specificity |
|---|---|---|---|---|
| Aged >80 years | 7.9% | 94.2% | 32.1% | 74.7% |
| Previous Stroke | 11.9% | 93.9% | 7.1% | 96.4% |
| Age >80 years and Previous Stroke | 8.4% | 94.5% | 37.9% | 72.1% |
| APA score ≥2 | 12.4% | 94.6% | 19.3% | 91.2% |

Table 3b The predictive values in the validation population

| | PPV | NPV | Sensitivity | Specificity |
|---|---|---|---|---|
| Aged >80 years | 9.8% | 91.3% | 38.5% | 64.6% |
| Previous Stroke | 18.2% | 93.6% | 46.2% | 79.2% |
| Age >80 years and Previous Stroke | 13.3% | 91.4% | 15.4% | 90.0% |
| APA score ≥2 | 24.4% | 96.9% | 76.9% | 75.4% |

PPV Positive Predictive Value
NPV Negative Predictive Value
APA Composite of Age >80 years, Previous stroke, elevated ACR. A score of 2 or 3 are regarded as high risk

For peer review only

TRIPOD Checklist: Prediction Model Development

| Section/Topic | Item | Checklist Item | Page |
|---|---|---|---|
| **Title and abstract** | | | |
| Title | 1 | Identify the study as developing and/or validating a multivariable prediction model, the target population, and the outcome to be predicted. | 1 |
| Abstract | 2 | Provide a summary of objectives, study design, setting, participants, sample size, predictors, outcome, statistical analysis, results, and conclusions. | 3 |
| **Introduction** | | | |
| Background and objectives | 3a | Explain the medical context (including whether diagnostic or prognostic) and rationale for developing or validating the multivariable prediction model, including references to existing models. | 6 |
| | 3b | Specify the objectives, including whether the study describes the development or validation of the model or both. | 7 |
| **Methods** | | | |
| Source of data | 4a | Describe the study design or source of data (e.g., randomized trial, cohort, or registry data), separately for the development and validation data sets, if applicable. | 7 |
| | 4b | Specify the key study dates, including start of accrual; end of accrual; and, if applicable, end of follow-up. | 7 |
| Participants | 5a | Specify key elements of the study setting (e.g., primary care, secondary care, general population) including number and location of centres. | 7 |
| | 5b | Describe eligibility criteria for participants. | 7 |
| | 5c | Give details of treatments received, if relevant. | 7-8 |
| Outcome | 6a | Clearly define the outcome that is predicted by the prediction model, including how and when assessed. | 9 |
| | 6b | Report any actions to blind assessment of the outcome to be predicted. | 9 |
| Predictors | 7a | Clearly define all predictors used in developing or validating the multivariable prediction model, including how and when they were measured. | 9-10 |
| | 7b | Report any actions to blind assessment of predictors for the outcome and other predictors. | 8 |
| Sample size | 8 | Explain how the study size was arrived at. | 9 |
| Missing data | 9 | Describe how missing data were handled (e.g., complete-case analysis, single imputation, multiple imputation) with details of any imputation method. | n/a |
| Statistical analysis methods | 10a | Describe how predictors were handled in the analyses. | 9-10 |
| | 10b | Specify type of model, all model-building procedures (including any predictor selection), and method for internal validation. | 9-10 |
| | 10d | Specify all measures used to assess model performance and, if relevant, to compare multiple models. | 9-10 |
| Risk groups | 11 | Provide details on how risk groups were created, if done. | |
| **Results** | | | |
| Participants | 13a | Describe the flow of participants through the study, including the number of participants with and without the outcome and, if applicable, a summary of the follow-up time. A diagram may be helpful. | 10 |
| | 13b | Describe the characteristics of the participants (basic demographics, clinical features, available predictors), including the number of participants with missing data for predictors and outcome. | 10 & t1 |
| Model development | 14a | Specify the number of participants and outcome events in each analysis. | 11 & t1 |
| | 14b | If done, report the unadjusted association between each candidate predictor and outcome. | n/a |
| Model specification | 15a | Present the full prediction model to allow predictions for individuals (i.e., all regression coefficients, and model intercept or baseline survival at a given time point). | 12 |
| | 15b | Explain how to the use the prediction model. | |
| Model performance | 16 | Report performance measures (with CIs) for the prediction model. | 13 |
| **Discussion** | | | |
| Limitations | 18 | Discuss any limitations of the study (such as nonrepresentative sample, few events per predictor, missing data). | 16 |
| Interpretation | 19b | Give an overall interpretation of the results, considering objectives, limitations, and results from similar studies, and other relevant evidence. | 14-15 |
| Implications | 20 | Discuss the potential clinical use of the model and implications for future research. | 15 |
| **Other information** | | | |
| Supplementary information | 21 | Provide information about the availability of supplementary resources, such as study protocol, Web calculator, and data sets. | ST1 |
| Funding | 22 | Give the source of funding and the role of the funders for the present study. | 19 |

We recommend using the TRIPOD Checklist in conjunction with the TRIPOD Explanation and Elaboration document.

For peer review only
